# Supplementary material for: Hybrid Models and Biological Model Reduction with PyDSTool
Source: PLoS Comput Biol. 2012 Aug 9;8(8):e1002628. doi: 10.1371/journal.pcbi.1002628 (PMC3415397; doi:10.1371/journal.pcbi.1002628)
Supplement: Text S4 — Complete source code for the PyDSTool package (version 0.88.120504). Includes API documentation and help files linking to web pages. This file is identical to the current public release on Sourceforge.net. (ZIP) [file pcbi.1002628.s004.zip › PyDSTool/html/toc-everything.html]

xml version="1.0" encoding="ascii"?


Everything


# Everything

---

## All Classes

PyDSTool.Events.Event  
 PyDSTool.Events.EventStruct  
 PyDSTool.Events.HighLevelEvent  
 PyDSTool.Events.LowLevelEvent  
 PyDSTool.Events.MatlabEvent  
 PyDSTool.FuncSpec'.ExpFuncSpec  
 PyDSTool.FuncSpec'.FuncSpec  
 PyDSTool.FuncSpec'.ImpFuncSpec  
 PyDSTool.FuncSpec'.RHSfuncSpec  
 PyDSTool.Generator.ADMC\_ODEsystem'.ADMC\_ODEsystem  
 PyDSTool.Generator.DDEsystem.DDEsystem  
 PyDSTool.Generator.Dopri\_ODEsystem'.Dopri\_ODEsystem  
 PyDSTool.Generator.Dopri\_ODEsystem'.dopri  
 PyDSTool.Generator.EmbeddedSysGen'.EmbeddedSysGen  
 PyDSTool.Generator.Euler\_ODEsystem'.Euler\_ODEsystem  
 PyDSTool.Generator.Euler\_ODEsystem'.euler\_solver  
 PyDSTool.Generator.ExplicitFnGen'.ExplicitFnGen  
 PyDSTool.Generator.ExtrapolateTable'.ExtrapolateTable  
 PyDSTool.Generator.ImplicitFnGen'.ImplicitFnGen  
 PyDSTool.Generator.InterpolateTable'.InterpolateTable  
 PyDSTool.Generator.LookupTable'.LookupTable  
 PyDSTool.Generator.MapSystem'.MapSystem  
 PyDSTool.Generator.ODEsystem'.ODEsystem  
 PyDSTool.Generator.Radau\_ODEsystem'.Radau\_ODEsystem  
 PyDSTool.Generator.Radau\_ODEsystem'.radau  
 PyDSTool.Generator.Vode\_ODEsystem'.Vode\_ODEsystem  

PyDSTool.Generator.baseclasses.GenSpecHelper

PyDSTool.Generator.baseclasses.GenSpecInfoObj

PyDSTool.Generator.baseclasses.Generator  
 PyDSTool.Generator.baseclasses.auxfn\_container  
 PyDSTool.Generator.baseclasses.ctsGen  
 PyDSTool.Generator.baseclasses.discGen  

PyDSTool.Generator.baseclasses.genDBClass

PyDSTool.Generator.baseclasses.ixmap

PyDSTool.Interval'.Interval  

PyDSTool.Interval'.IntervalMembership

PyDSTool.MProject.GenTransform  
 PyDSTool.MProject.GeneratorInterface  

PyDSTool.MProject.MReg

PyDSTool.MProject.ModelInterface  
 PyDSTool.MProject.ModelLibrary  
 PyDSTool.MProject.ModelManager  
 PyDSTool.MProject.ModelTransform  
 PyDSTool.MProject.always\_feature  
 PyDSTool.MProject.binary\_feature  
 PyDSTool.MProject.condition  
 PyDSTool.MProject.context  

PyDSTool.MProject.dsInterface

PyDSTool.MProject.extModelInterface  
 PyDSTool.MProject.feature  

PyDSTool.MProject.feature\_leaf

PyDSTool.MProject.feature\_node

PyDSTool.MProject.intModelInterface  
 PyDSTool.MProject.ql\_feature\_leaf  
 PyDSTool.MProject.ql\_feature\_node  
 PyDSTool.MProject.qt\_feature\_leaf  
 PyDSTool.MProject.qt\_feature\_node  
 PyDSTool.Model.HybridModel  
 PyDSTool.Model.Model  
 PyDSTool.Model.NonHybridModel  
 PyDSTool.Model.boundary\_containment  
 PyDSTool.Model.boundary\_containment\_by\_event  
 PyDSTool.Model.boundary\_containment\_by\_postproc  
 PyDSTool.Model.domain\_test  

PyDSTool.ModelConstructor'.Descriptor

PyDSTool.ModelConstructor'.EvMapping  
 PyDSTool.ModelConstructor'.GDescriptor  
 PyDSTool.ModelConstructor'.GeneratorConstructor  
 PyDSTool.ModelConstructor'.MDescriptor  
 PyDSTool.ModelConstructor'.ModelConstructor  
 PyDSTool.ModelSpec'.Component  
 PyDSTool.ModelSpec'.LeafComponent  
 PyDSTool.ModelSpec'.ModelSpec  
 PyDSTool.ModelSpec'.nameResolverClass  

PyDSTool.ModelSpec'.regObject

PyDSTool.ModelSpec'.typeCounter

PyDSTool.Points.Point  
 PyDSTool.Points.PointInfo  
 PyDSTool.Points.Pointset  
 PyDSTool.Points.VarCaller  
 PyDSTool.PyCont.BifPoint.BPoint  
 PyDSTool.PyCont.BifPoint.BTPoint  
 PyDSTool.PyCont.BifPoint.BifPoint  
 PyDSTool.PyCont.BifPoint.BranchPoint  
 PyDSTool.PyCont.BifPoint.CPPoint  
 PyDSTool.PyCont.BifPoint.DHPoint  
 PyDSTool.PyCont.BifPoint.FoldPoint  
 PyDSTool.PyCont.BifPoint.GHPoint  
 PyDSTool.PyCont.BifPoint.HopfPoint  
 PyDSTool.PyCont.BifPoint.LPCPoint  
 PyDSTool.PyCont.BifPoint.NSPoint  
 PyDSTool.PyCont.BifPoint.PDPoint  
 PyDSTool.PyCont.BifPoint.SPoint  
 PyDSTool.PyCont.BifPoint.ZHPoint  
 PyDSTool.PyCont.ContClass'.ContClass  
 PyDSTool.PyCont.Continuation.Continuation  
 PyDSTool.PyCont.Continuation.EquilibriumCurve  
 PyDSTool.PyCont.Continuation.FixedPointCurve  
 PyDSTool.PyCont.Continuation.FoldCurve  
 PyDSTool.PyCont.Continuation.HopfCurveOne  
 PyDSTool.PyCont.Continuation.HopfCurveTwo  
 PyDSTool.PyCont.Continuation.LimitCycleCurve  
 PyDSTool.PyCont.Continuation.UserDefinedCurve  
 PyDSTool.PyCont.Plotting.KeyEvent  
 PyDSTool.PyCont.Plotting.pargs  
 PyDSTool.PyCont.TestFunc.AddTestFunction  
 PyDSTool.PyCont.TestFunc.BT\_Fold  
 PyDSTool.PyCont.TestFunc.BT\_Hopf  
 PyDSTool.PyCont.TestFunc.BT\_Hopf\_One  
 PyDSTool.PyCont.TestFunc.B\_Check  
 PyDSTool.PyCont.TestFunc.BiAltMethod  
 PyDSTool.PyCont.TestFunc.BorderMethod  
 PyDSTool.PyCont.TestFunc.Branch\_Bor  
 PyDSTool.PyCont.TestFunc.Branch\_Det  
 PyDSTool.PyCont.TestFunc.CP\_Fold  
 PyDSTool.PyCont.TestFunc.DH\_Hopf  
 PyDSTool.PyCont.TestFunc.DiscreteMap  
 PyDSTool.PyCont.TestFunc.FixedPointMap  
 PyDSTool.PyCont.TestFunc.Fold\_Bor  
 PyDSTool.PyCont.TestFunc.Fold\_Det  
 PyDSTool.PyCont.TestFunc.Fold\_Tan  
 PyDSTool.PyCont.TestFunc.Function  
 PyDSTool.PyCont.TestFunc.GH\_Hopf  
 PyDSTool.PyCont.TestFunc.GH\_Hopf\_One  
 PyDSTool.PyCont.TestFunc.Hopf\_Bor  
 PyDSTool.PyCont.TestFunc.Hopf\_Det  
 PyDSTool.PyCont.TestFunc.Hopf\_Double\_Bor\_One  
 PyDSTool.PyCont.TestFunc.Hopf\_Double\_Bor\_Two  
 PyDSTool.PyCont.TestFunc.Hopf\_Eig  
 PyDSTool.PyCont.TestFunc.LPC\_Det  
 PyDSTool.PyCont.TestFunc.NS\_Det  
 PyDSTool.PyCont.TestFunc.PD\_Det  
 PyDSTool.PyCont.TestFunc.ParTestFunc  
 PyDSTool.PyCont.TestFunc.TestFunc  
 PyDSTool.PyCont.TestFunc.UserDefinedTestFunc  
 PyDSTool.PyCont.misc.IterationError  
 PyDSTool.PyCont.misc.Struct  
 PyDSTool.Redirector.Redirector  
 PyDSTool.Symbolic.Fun  
 PyDSTool.Symbolic.Input  
 PyDSTool.Symbolic.Par  
 PyDSTool.Symbolic.QuantSpec  
 PyDSTool.Symbolic.Quantity  
 PyDSTool.Symbolic.Var  

PyDSTool.Symbolic.\_mathobj

PyDSTool.Toolbox.DSSRT\_tools.DSSRT\_info  
 PyDSTool.Toolbox.FR.cool  
 PyDSTool.Toolbox.FR.edge  
 PyDSTool.Toolbox.FR.vertex  
 PyDSTool.Toolbox.FSM.ExceptionFSM  
 PyDSTool.Toolbox.FSM.FSM  
 PyDSTool.Toolbox.FSM.ObjFSM  
 PyDSTool.Toolbox.ModelEst.ModelEst  
 PyDSTool.Toolbox.ModelHelper.SpiffyODEModel  
 PyDSTool.Toolbox.NineML.NineMLModel  
 PyDSTool.Toolbox.ParamEst.BoundMin  
 PyDSTool.Toolbox.ParamEst.ConstraintFail  
 PyDSTool.Toolbox.ParamEst.Converged  
 PyDSTool.Toolbox.ParamEst.L2\_feature  
 PyDSTool.Toolbox.ParamEst.L2\_feature\_1D  
 PyDSTool.Toolbox.ParamEst.LMpest  
 PyDSTool.Toolbox.ParamEst.ParamEst  
 PyDSTool.Toolbox.ParamEst.residual\_fn\_context  
 PyDSTool.Toolbox.ParamEst.residual\_fn\_context\_1D  
 PyDSTool.Toolbox.data\_analysis.data\_bins  
 PyDSTool.Toolbox.dataanalysis.data\_bins  
 PyDSTool.Toolbox.dssrt.EpochSeqScorer  
 PyDSTool.Toolbox.dssrt.Scorer  
 PyDSTool.Toolbox.dssrt.VarAlphabet  
 PyDSTool.Toolbox.dssrt.become\_most\_dominant  
 PyDSTool.Toolbox.dssrt.domscales  
 PyDSTool.Toolbox.dssrt.dssrt\_assistant  
 PyDSTool.Toolbox.dssrt.epoch  
 PyDSTool.Toolbox.dssrt.is\_active  
 PyDSTool.Toolbox.dssrt.is\_fast  
 PyDSTool.Toolbox.dssrt.is\_inactive  
 PyDSTool.Toolbox.dssrt.is\_modulatory  
 PyDSTool.Toolbox.dssrt.is\_most\_dominant  
 PyDSTool.Toolbox.dssrt.is\_order1  
 PyDSTool.Toolbox.dssrt.is\_slow  
 PyDSTool.Toolbox.dssrt.join\_actives  
 PyDSTool.Toolbox.dssrt.join\_fast  
 PyDSTool.Toolbox.dssrt.join\_slow  
 PyDSTool.Toolbox.dssrt.leave\_actives  
 PyDSTool.Toolbox.dssrt.leave\_fast  
 PyDSTool.Toolbox.dssrt.leave\_slow  
 PyDSTool.Toolbox.dssrt.regime  

PyDSTool.Toolbox.event\_driven\_simulator.FIFOqueue\_uniquenode

PyDSTool.Toolbox.event\_driven\_simulator.composed\_map1D  
 PyDSTool.Toolbox.event\_driven\_simulator.connection  
 PyDSTool.Toolbox.event\_driven\_simulator.delay\_map  
 PyDSTool.Toolbox.event\_driven\_simulator.identity\_map  
 PyDSTool.Toolbox.event\_driven\_simulator.map  
 PyDSTool.Toolbox.event\_driven\_simulator.map1D  
 PyDSTool.Toolbox.event\_driven\_simulator.map2D  
 PyDSTool.Toolbox.event\_driven\_simulator.node  
 PyDSTool.Toolbox.event\_driven\_simulator.simulator  
 PyDSTool.Toolbox.makeSloppyModel.sloppyModel  
 PyDSTool.Toolbox.neuralcomp.channel  
 PyDSTool.Toolbox.neuralcomp.channel\_off  
 PyDSTool.Toolbox.neuralcomp.channel\_on  
 PyDSTool.Toolbox.neuralcomp.compartment  
 PyDSTool.Toolbox.neuralcomp.compatODEComponent  
 PyDSTool.Toolbox.neuralcomp.compatODELeafComponent  
 PyDSTool.Toolbox.neuralcomp.dendr\_compartment  
 PyDSTool.Toolbox.neuralcomp.exc\_synapse  
 PyDSTool.Toolbox.neuralcomp.inh\_synapse  
 PyDSTool.Toolbox.neuralcomp.network  
 PyDSTool.Toolbox.neuralcomp.neurite\_compartment  
 PyDSTool.Toolbox.neuralcomp.neuron  
 PyDSTool.Toolbox.neuralcomp.pnnetwork  
 PyDSTool.Toolbox.neuralcomp.soma  
 PyDSTool.Toolbox.neuralcomp.synapse  
 PyDSTool.Toolbox.neuro\_data.burst\_feature  
 PyDSTool.Toolbox.neuro\_data.estimate\_spiking  
 PyDSTool.Toolbox.neuro\_data.geom\_feature  
 PyDSTool.Toolbox.neuro\_data.get\_burst\_active\_phase  
 PyDSTool.Toolbox.neuro\_data.get\_burst\_dc\_offset  
 PyDSTool.Toolbox.neuro\_data.get\_burst\_downsweep  
 PyDSTool.Toolbox.neuro\_data.get\_burst\_duration  
 PyDSTool.Toolbox.neuro\_data.get\_burst\_isi\_env  
 PyDSTool.Toolbox.neuro\_data.get\_burst\_num\_spikes  
 PyDSTool.Toolbox.neuro\_data.get\_burst\_passive\_extent  
 PyDSTool.Toolbox.neuro\_data.get\_burst\_peak\_env  
 PyDSTool.Toolbox.neuro\_data.get\_burst\_period\_info  
 PyDSTool.Toolbox.neuro\_data.get\_burst\_spikes  
 PyDSTool.Toolbox.neuro\_data.get\_burst\_trough\_env  
 PyDSTool.Toolbox.neuro\_data.get\_burst\_upsweep  
 PyDSTool.Toolbox.neuro\_data.get\_spike\_data  
 PyDSTool.Toolbox.neuro\_data.get\_spike\_model  
 PyDSTool.Toolbox.neuro\_data.spike\_envelope  
 PyDSTool.Toolbox.neuro\_data.spike\_feature  
 PyDSTool.Toolbox.neuro\_data.spike\_metric  

PyDSTool.Toolbox.optimizers.criterion.composite\_criteria.AndComposition

PyDSTool.Toolbox.optimizers.criterion.composite\_criteria.OrComposition

PyDSTool.Toolbox.optimizers.criterion.criteria.AbsoluteParametersCriterion

PyDSTool.Toolbox.optimizers.criterion.criteria.AbsoluteValueCriterion

PyDSTool.Toolbox.optimizers.criterion.criteria.GradientCriterion

PyDSTool.Toolbox.optimizers.criterion.criteria.IterationCriterion

PyDSTool.Toolbox.optimizers.criterion.criteria.MonotonyCriterion

PyDSTool.Toolbox.optimizers.criterion.criteria.RelativeParametersCriterion

PyDSTool.Toolbox.optimizers.criterion.criteria.RelativeValueCriterion

PyDSTool.Toolbox.optimizers.criterion.information\_criteria.AICCriterion

PyDSTool.Toolbox.optimizers.criterion.information\_criteria.ModifiedAICCriterion

PyDSTool.Toolbox.optimizers.helpers.finite\_difference.CenteredFiniteDifferences

PyDSTool.Toolbox.optimizers.helpers.finite\_difference.FiniteDifferencesCache

PyDSTool.Toolbox.optimizers.helpers.finite\_difference.FiniteDifferencesFunction

PyDSTool.Toolbox.optimizers.helpers.finite\_difference.ForwardFiniteDifferences

PyDSTool.Toolbox.optimizers.helpers.finite\_difference.ForwardFiniteDifferencesCache

PyDSTool.Toolbox.optimizers.helpers.levenberg\_marquardt.LMQuadratic  

PyDSTool.Toolbox.optimizers.helpers.quadratic.Quadratic

PyDSTool.Toolbox.optimizers.line\_search.adaptive\_last\_step\_modifier.AdaptiveLastStepModifier

PyDSTool.Toolbox.optimizers.line\_search.backtracking\_search.BacktrackingSearch

PyDSTool.Toolbox.optimizers.line\_search.cubic\_interpolation.CubicInterpolationSearch

PyDSTool.Toolbox.optimizers.line\_search.damped\_line\_search.DampedLineSearch

PyDSTool.Toolbox.optimizers.line\_search.fibonacci\_section.FibonacciSectionSearch

PyDSTool.Toolbox.optimizers.line\_search.fixed\_last\_step\_modifier.FixedLastStepModifier

PyDSTool.Toolbox.optimizers.line\_search.golden\_section.GoldenSectionSearch

PyDSTool.Toolbox.optimizers.line\_search.goldstein\_rule.GoldsteinRule

PyDSTool.Toolbox.optimizers.line\_search.hyperbolic\_line\_search.HyperbolicLineSearch

PyDSTool.Toolbox.optimizers.line\_search.quadratic\_interpolation.QuadraticInterpolationSearch

PyDSTool.Toolbox.optimizers.line\_search.scaled\_line\_search.ScaledLineSearch

PyDSTool.Toolbox.optimizers.line\_search.simple\_line\_search.SimpleLineSearch

PyDSTool.Toolbox.optimizers.line\_search.strong\_wolfe\_powell\_rule.StrongWolfePowellRule

PyDSTool.Toolbox.optimizers.line\_search.wolfe\_powell\_rule.WolfePowellRule

PyDSTool.Toolbox.optimizers.optimizer.optimizer.Optimizer

PyDSTool.Toolbox.optimizers.optimizer.standard\_optimizer.StandardOptimizer

PyDSTool.Toolbox.optimizers.optimizer.standard\_optimizer\_modifying.StandardOptimizerModifying

PyDSTool.Toolbox.optimizers.step.conjugate\_gradient\_step.ConjugateGradientStep

PyDSTool.Toolbox.optimizers.step.goldfeld\_step.GoldfeldStep

PyDSTool.Toolbox.optimizers.step.goldstein\_price\_step.GoldsteinPriceStep

PyDSTool.Toolbox.optimizers.step.gradient\_step.GradientStep

PyDSTool.Toolbox.optimizers.step.local\_brute\_force\_1dstep.LocalBruteForce1DStep

PyDSTool.Toolbox.optimizers.step.marquardt\_step.MarquardtStep

PyDSTool.Toolbox.optimizers.step.newton\_step.NewtonStep

PyDSTool.Toolbox.optimizers.step.partial\_step.PartialStep

PyDSTool.Toolbox.optimizers.step.quasi\_newton\_step.DFPNewtonStep

PyDSTool.Toolbox.optimizers.step.restart\_conjugate\_gradient.RestartNotOrthogonalConjugateGradientStep

PyDSTool.Toolbox.optimizers.step.restart\_conjugate\_gradient.RestartPeriodicallyConjugateGradientStep

PyDSTool.Toolbox.optimizers.tests.test\_powell.Powell  
 PyDSTool.Toolbox.optimizers.tests.test\_powell.test\_Powell  
 PyDSTool.Toolbox.optimizers.tests.test\_quadratic.Quadratic  
 PyDSTool.Toolbox.optimizers.tests.test\_quadratic.test\_Quadratic  
 PyDSTool.Toolbox.optimizers.tests.test\_rosenbrock.Rosenbrock  
 PyDSTool.Toolbox.optimizers.tests.test\_rosenbrock.test\_Rosenbrock  
 PyDSTool.Toolbox.phaseplane.Point2D  

PyDSTool.Toolbox.phaseplane.base\_n\_counter

PyDSTool.Toolbox.phaseplane.distance\_to\_pointset  
 PyDSTool.Toolbox.phaseplane.dx\_scaled\_2D  
 PyDSTool.Toolbox.phaseplane.fixedpoint\_2D  
 PyDSTool.Toolbox.phaseplane.fixedpoint\_nD  

PyDSTool.Toolbox.phaseplane.fixedpoint\_zone

PyDSTool.Toolbox.phaseplane.inflection\_zone\_leaf  
 PyDSTool.Toolbox.phaseplane.inflection\_zone\_node  
 PyDSTool.Toolbox.phaseplane.max\_curvature\_zone\_leaf  
 PyDSTool.Toolbox.phaseplane.max\_curvature\_zone\_node  
 PyDSTool.Toolbox.phaseplane.mesh\_patch\_2D  

PyDSTool.Toolbox.phaseplane.min\_curvature\_zone

PyDSTool.Toolbox.phaseplane.nullcline  

PyDSTool.Toolbox.phaseplane.nullcline\_zone\_leaf

PyDSTool.Toolbox.phaseplane.nullcline\_zone\_node

PyDSTool.Toolbox.phaseplane.phaseplane  
 PyDSTool.Toolbox.phaseplane.plotter\_2D  

PyDSTool.Toolbox.phaseplane.zone\_leaf

PyDSTool.Toolbox.phaseplane.zone\_node

PyDSTool.Toolbox.test\_protocols.virtual\_experiment  
 PyDSTool.Trajectory'.HybridTrajectory  
 PyDSTool.Trajectory'.Trajectory  
 PyDSTool.Variable'.HybridVariable  
 PyDSTool.Variable'.OutputFn  

PyDSTool.Variable'.VarDiagnostics

PyDSTool.Variable'.Variable  

PyDSTool.common.API\_class

PyDSTool.common.BarycentricInterpolator  
 PyDSTool.common.DefaultDict  
 PyDSTool.common.Diagnostics  

PyDSTool.common.DomainType

PyDSTool.common.KroghInterpolator  
 PyDSTool.common.PiecewisePolynomial  
 PyDSTool.common.Struct  
 PyDSTool.common.Utility  
 PyDSTool.common.Verbose  
 PyDSTool.common.and\_op  
 PyDSTool.common.args  
 PyDSTool.common.fit\_cubic  
 PyDSTool.common.fit\_diff\_of\_exp  
 PyDSTool.common.fit\_exponential  

PyDSTool.common.fit\_function

PyDSTool.common.fit\_linear  
 PyDSTool.common.fit\_quadratic  
 PyDSTool.common.fit\_quadratic\_at\_vertex  
 PyDSTool.common.interp0d  
 PyDSTool.common.interp1d  
 PyDSTool.common.interpclass  
 PyDSTool.common.metric  
 PyDSTool.common.metric\_L2  
 PyDSTool.common.metric\_L2\_1D  
 PyDSTool.common.metric\_float  
 PyDSTool.common.metric\_float\_1D  
 PyDSTool.common.metric\_weighted\_L2  
 PyDSTool.common.metric\_weighted\_deadzone\_L2  
 PyDSTool.common.not\_op  

PyDSTool.common.null\_predicate\_class

PyDSTool.common.or\_op  
 PyDSTool.common.predicate  

PyDSTool.common.predicate\_op

PyDSTool.errors.PyDSTool\_AttributeError  
 PyDSTool.errors.PyDSTool\_BoundsError  
 PyDSTool.errors.PyDSTool\_ClearError  
 PyDSTool.errors.PyDSTool\_ContError  
 PyDSTool.errors.PyDSTool\_Error  
 PyDSTool.errors.PyDSTool\_ExistError  
 PyDSTool.errors.PyDSTool\_InitError  
 PyDSTool.errors.PyDSTool\_KeyError  
 PyDSTool.errors.PyDSTool\_TypeError  
 PyDSTool.errors.PyDSTool\_UncertainValueError  
 PyDSTool.errors.PyDSTool\_UndefinedError  
 PyDSTool.errors.PyDSTool\_ValueError  
 PyDSTool.fixedpickle.PickleError  
 PyDSTool.fixedpickle.Pickler  
 PyDSTool.fixedpickle.PicklingError  
 PyDSTool.fixedpickle.Unpickler  
 PyDSTool.fixedpickle.UnpicklingError  

PyDSTool.fixedpickle.\_EmptyClass

PyDSTool.fixedpickle.\_Stop

PyDSTool.integrator'.integrator  
 PyDSTool.parseUtils.auxfnDBclass  
 PyDSTool.parseUtils.parserObject  
 PyDSTool.parseUtils.symbolMapClass  

PyDSTool.scipy\_ode.IntegratorBase

PyDSTool.scipy\_ode.ode  

PyDSTool.scipy\_ode.vode

cPickle.BadPickleGet  
 cPickle.PickleError  
 cPickle.PicklingError  
 cPickle.UnpickleableError  
 cPickle.UnpicklingError  

## All Functions

PyDSTool.Events.\_active

PyDSTool.Events.\_highlevel

PyDSTool.Events.\_lowlevel

PyDSTool.Events.\_nonterm

PyDSTool.Events.\_notactive

PyDSTool.Events.\_notprecise

PyDSTool.Events.\_notvarlinked

PyDSTool.Events.\_precise

PyDSTool.Events.\_term

PyDSTool.Events.\_varlinked

PyDSTool.Events.collectReused

PyDSTool.Events.findpreciseroot

PyDSTool.Events.makePythonStateZeroCrossEvent  
 PyDSTool.Events.makeZeroCrossEvent  

PyDSTool.Events.processReusedC

PyDSTool.Events.processReusedMatlab

PyDSTool.Events.processReusedPy

PyDSTool.FuncSpec'.\_processReused

PyDSTool.FuncSpec'.getSpecFromFile  
 PyDSTool.FuncSpec'.makePartialJac  
 PyDSTool.FuncSpec'.resolveClashingAuxFnPars  
 PyDSTool.Generator.Dopri\_ODEsystem'.distutil\_destination  

PyDSTool.Generator.Euler\_ODEsystem'.\_dummy\_userfunc

PyDSTool.Generator.Radau\_ODEsystem'.distutil\_destination  
 PyDSTool.Generator.baseclasses.\_pollInputs  
 PyDSTool.Generator.findGenSubClasses  
 PyDSTool.Interval'.isinterval  
 PyDSTool.Interval'.issingleton  
 PyDSTool.MProject.extract\_from\_model  

PyDSTool.MProject.process\_raw\_residual

PyDSTool.Model.findTrajInitiator

PyDSTool.Model.getAuxVars

PyDSTool.ModelConstructor'.embed  
 PyDSTool.ModelConstructor'.makeEvMapping  
 PyDSTool.ModelConstructor'.makeModelInfo  
 PyDSTool.ModelConstructor'.makeModelInfoEntry  

PyDSTool.ModelConstructor'.processReused

PyDSTool.ModelConstructor'.validateTransitionName

PyDSTool.ModelSpec'.accrueCompTypes

PyDSTool.ModelSpec'.allCompTypes

PyDSTool.ModelSpec'.matchSubName  
 PyDSTool.ModelSpec'.processMacro  
 PyDSTool.ModelSpec'.resolveMacroTargets  
 PyDSTool.ModelSpec'.searchModelSpec  

PyDSTool.Points.\_pretty\_print\_label

PyDSTool.Points.arrayToPointset  
 PyDSTool.Points.comparePointCoords  
 PyDSTool.Points.exportPointset  
 PyDSTool.Points.export\_pointset\_to\_CSV  
 PyDSTool.Points.importPointset  
 PyDSTool.Points.isparameterized  
 PyDSTool.Points.makeNonParameterized  
 PyDSTool.Points.mergePointsets  
 PyDSTool.Points.padPointset  
 PyDSTool.Points.pointsToPointset  

PyDSTool.PyCont.ContClass'.distutil\_destination

PyDSTool.PyCont.Plotting.initializeDisplay  
 PyDSTool.PyCont.misc.CheckHopf  
 PyDSTool.PyCont.misc.bialttoeig  
 PyDSTool.PyCont.misc.bilinearform  

PyDSTool.PyCont.misc.evec1\_standard

PyDSTool.PyCont.misc.firstlyapunov  
 PyDSTool.PyCont.misc.getFlowJac  
 PyDSTool.PyCont.misc.getFlowMaps  
 PyDSTool.PyCont.misc.getLeftEvecs  
 PyDSTool.PyCont.misc.hess  
 PyDSTool.PyCont.misc.hess3  
 PyDSTool.PyCont.misc.ijtoind  
 PyDSTool.PyCont.misc.indtoij  
 PyDSTool.PyCont.misc.invwedge  
 PyDSTool.PyCont.misc.isnotzero  
 PyDSTool.PyCont.misc.iszero  
 PyDSTool.PyCont.misc.jac  
 PyDSTool.PyCont.misc.monotone  

PyDSTool.PyCont.misc.negate

PyDSTool.PyCont.misc.partition  
 PyDSTool.PyCont.misc.testindij  
 PyDSTool.PyCont.misc.tocoords  
 PyDSTool.PyCont.misc.todict  
 PyDSTool.PyCont.misc.trilinearform  
 PyDSTool.PyCont.misc.unique  
 PyDSTool.PyCont.misc.wedge  
 PyDSTool.Symbolic.Diff  
 PyDSTool.Symbolic.DiffStr  

PyDSTool.Symbolic.\_eval

PyDSTool.Symbolic.\_generate\_subderivatives

PyDSTool.Symbolic.\_join\_sublist

PyDSTool.Symbolic.\_parse\_func\_deriv

PyDSTool.Symbolic.\_propagate\_str\_eval

PyDSTool.Symbolic.checkbraces  

PyDSTool.Symbolic.dofun

PyDSTool.Symbolic.ensureQlist  
 PyDSTool.Symbolic.ensureStrArgDict  
 PyDSTool.Symbolic.evalMultiRefToken  
 PyDSTool.Symbolic.expr2fun  
 PyDSTool.Symbolic.findMultiRefs  

PyDSTool.Symbolic.getdim

PyDSTool.Symbolic.getlftrtD

PyDSTool.Symbolic.isMultiDef  
 PyDSTool.Symbolic.isMultiDefClash  
 PyDSTool.Symbolic.isMultiRef  
 PyDSTool.Symbolic.prepJacobian  

PyDSTool.Symbolic.processMultiDef

PyDSTool.Symbolic.processMultiRef  

PyDSTool.Symbolic.resolveSpecTypeCombos

PyDSTool.Symbolic.strIfSeq

PyDSTool.Symbolic.subs  

PyDSTool.Symbolic.whoQ

PyDSTool.Toolbox.ActivationFuncs.Beta  
 PyDSTool.Toolbox.ActivationFuncs.Gamma  
 PyDSTool.Toolbox.ActivationFuncs.Sigma  
 PyDSTool.Toolbox.ActivationFuncs.Sigma2  
 PyDSTool.Toolbox.ActivationFuncs.SigmaNeg  
 PyDSTool.Toolbox.ActivationFuncs.SigmaV  
 PyDSTool.Toolbox.DSSRT\_tools.plotNetworkGraph  
 PyDSTool.Toolbox.FR.FR  
 PyDSTool.Toolbox.FR.cool\_simple  
 PyDSTool.Toolbox.FR.in\_degree  
 PyDSTool.Toolbox.FR.itercool  
 PyDSTool.Toolbox.FR.out\_degree  
 PyDSTool.Toolbox.InputProfile.makeInputTable  
 PyDSTool.Toolbox.InputProfile.makeSpikeProfile  
 PyDSTool.Toolbox.InputProfile.plotProfile  
 PyDSTool.Toolbox.ModelHelper.makeSpiffyODEModel  
 PyDSTool.Toolbox.NineML.get\_nineml\_model  

PyDSTool.Toolbox.NineML.get\_regime\_model

PyDSTool.Toolbox.PRCtools.\_default\_pert

PyDSTool.Toolbox.PRCtools.compare\_pert  
 PyDSTool.Toolbox.PRCtools.finitePRC  
 PyDSTool.Toolbox.PRCtools.fix\_PRC  
 PyDSTool.Toolbox.PRCtools.one\_period\_traj  

PyDSTool.Toolbox.ParamEst.\_makeUnique

PyDSTool.Toolbox.ParamEst.\_present\_and\_sensitive

PyDSTool.Toolbox.ParamEst.compare\_data\_from\_events  
 PyDSTool.Toolbox.ParamEst.ctn\_residual\_info  
 PyDSTool.Toolbox.ParamEst.do\_2Ddirn  
 PyDSTool.Toolbox.ParamEst.do\_2Dstep  
 PyDSTool.Toolbox.ParamEst.filter\_feats  
 PyDSTool.Toolbox.ParamEst.filter\_iface  
 PyDSTool.Toolbox.ParamEst.filter\_pars  
 PyDSTool.Toolbox.ParamEst.get\_extrema  
 PyDSTool.Toolbox.ParamEst.get\_extrema\_from\_events  
 PyDSTool.Toolbox.ParamEst.get\_slope\_info  
 PyDSTool.Toolbox.ParamEst.grad\_from\_psens  
 PyDSTool.Toolbox.ParamEst.make\_opt  
 PyDSTool.Toolbox.ParamEst.norm\_D\_sum  
 PyDSTool.Toolbox.ParamEst.organize\_feature\_sens  

PyDSTool.Toolbox.ParamEst.pp

PyDSTool.Toolbox.ParamEst.restrict\_opt  
 PyDSTool.Toolbox.ParamEst.select\_pars\_for\_features  
 PyDSTool.Toolbox.ParamEst.sweep1D  
 PyDSTool.Toolbox.PySCes\_SBML.get\_pysces\_model  

PyDSTool.Toolbox.PySCes\_SBML.make\_varspecs

PyDSTool.Toolbox.adjointPRC.adjointPRC  

PyDSTool.Toolbox.adjointPRC.find\_zero\_phase

PyDSTool.Toolbox.adjointPRC.rotate\_phase  

PyDSTool.Toolbox.data\_analysis.PCANode

PyDSTool.Toolbox.data\_analysis.\_adjust\_ix

PyDSTool.Toolbox.data\_analysis.colormap

PyDSTool.Toolbox.data\_analysis.dist\_between\_datasets  
 PyDSTool.Toolbox.data\_analysis.doPCA  

PyDSTool.Toolbox.data\_analysis.dump\_progress

PyDSTool.Toolbox.data\_analysis.filtfilt  
 PyDSTool.Toolbox.data\_analysis.find\_central\_point  
 PyDSTool.Toolbox.data\_analysis.find\_closest\_val  
 PyDSTool.Toolbox.data\_analysis.find\_diameter  
 PyDSTool.Toolbox.data\_analysis.find\_from\_sorted  
 PyDSTool.Toolbox.data\_analysis.find\_knees  
 PyDSTool.Toolbox.data\_analysis.find\_nearby\_annulus  
 PyDSTool.Toolbox.data\_analysis.find\_nearby\_ball  
 PyDSTool.Toolbox.data\_analysis.find\_recurrences  
 PyDSTool.Toolbox.data\_analysis.fitline  
 PyDSTool.Toolbox.data\_analysis.get\_evec  
 PyDSTool.Toolbox.data\_analysis.get\_linear\_regression\_residual  
 PyDSTool.Toolbox.data\_analysis.get\_residual  
 PyDSTool.Toolbox.data\_analysis.lfilter\_zi  
 PyDSTool.Toolbox.data\_analysis.log\_distances  
 PyDSTool.Toolbox.data\_analysis.log\_distances\_with\_D  
 PyDSTool.Toolbox.data\_analysis.out\_of\_seq  
 PyDSTool.Toolbox.data\_analysis.pca\_dim  
 PyDSTool.Toolbox.data\_analysis.plot\_PCA\_residuals  
 PyDSTool.Toolbox.data\_analysis.plot\_PCA\_spectrum  
 PyDSTool.Toolbox.data\_analysis.rectify  

PyDSTool.Toolbox.data\_analysis.recurrence\_times

PyDSTool.Toolbox.data\_analysis.second\_diff

PyDSTool.Toolbox.data\_analysis.whiten  

PyDSTool.Toolbox.dataanalysis.\_adjust\_ix

PyDSTool.Toolbox.dataanalysis.colormap

PyDSTool.Toolbox.dataanalysis.dist\_between\_datasets  
 PyDSTool.Toolbox.dataanalysis.doPCA  

PyDSTool.Toolbox.dataanalysis.dump\_progress

PyDSTool.Toolbox.dataanalysis.find\_central\_point  
 PyDSTool.Toolbox.dataanalysis.find\_closest\_val  
 PyDSTool.Toolbox.dataanalysis.find\_diameter  
 PyDSTool.Toolbox.dataanalysis.find\_from\_sorted  
 PyDSTool.Toolbox.dataanalysis.find\_knees  
 PyDSTool.Toolbox.dataanalysis.find\_nearby\_annulus  
 PyDSTool.Toolbox.dataanalysis.find\_nearby\_ball  
 PyDSTool.Toolbox.dataanalysis.find\_recurrences  
 PyDSTool.Toolbox.dataanalysis.fitline  
 PyDSTool.Toolbox.dataanalysis.get\_evec  
 PyDSTool.Toolbox.dataanalysis.get\_linear\_regression\_residual  
 PyDSTool.Toolbox.dataanalysis.get\_residual  
 PyDSTool.Toolbox.dataanalysis.log\_distances  
 PyDSTool.Toolbox.dataanalysis.log\_distances\_with\_D  
 PyDSTool.Toolbox.dataanalysis.out\_of\_seq  
 PyDSTool.Toolbox.dataanalysis.pca\_dim  
 PyDSTool.Toolbox.dataanalysis.plot\_PCA\_residuals  
 PyDSTool.Toolbox.dataanalysis.plot\_PCA\_spectrum  

PyDSTool.Toolbox.dataanalysis.recurrence\_times

PyDSTool.Toolbox.dataanalysis.second\_diff

PyDSTool.Toolbox.dataanalysis.whiten  

PyDSTool.Toolbox.dssrt.\_comp

PyDSTool.Toolbox.dssrt.as\_absolute\_ratios

PyDSTool.Toolbox.dssrt.as\_relative\_ratios

PyDSTool.Toolbox.dssrt.check\_opts

PyDSTool.Toolbox.dssrt.comp\_seqs  
 PyDSTool.Toolbox.dssrt.define\_psi\_events  
 PyDSTool.Toolbox.dssrt.define\_tau\_events  
 PyDSTool.Toolbox.dssrt.editdist\_edits  
 PyDSTool.Toolbox.dssrt.find\_ep\_ix  
 PyDSTool.Toolbox.dssrt.find\_epoch\_period  
 PyDSTool.Toolbox.dssrt.find\_regime\_transition  
 PyDSTool.Toolbox.dssrt.get\_infs  
 PyDSTool.Toolbox.dssrt.get\_symbol\_sequence  
 PyDSTool.Toolbox.dssrt.get\_taus  
 PyDSTool.Toolbox.dssrt.indent  
 PyDSTool.Toolbox.dssrt.jaro  
 PyDSTool.Toolbox.dssrt.normalized\_psis  

PyDSTool.Toolbox.dssrt.only\_dynamic

PyDSTool.Toolbox.dssrt.pad

PyDSTool.Toolbox.dssrt.partition\_range

PyDSTool.Toolbox.dssrt.plot\_psis  
 PyDSTool.Toolbox.dssrt.show\_epochs  

PyDSTool.Toolbox.dssrt.spectral\_gaps\_absolute

PyDSTool.Toolbox.dssrt.spectral\_gaps\_relative

PyDSTool.Toolbox.dssrt.split

PyDSTool.Toolbox.dssrt.split\_pts  

PyDSTool.Toolbox.dssrt.strip\_speed

PyDSTool.Toolbox.dssrt.swdist  
 PyDSTool.Toolbox.dssrt.tabulate\_epoch\_seqs  
 PyDSTool.Toolbox.dssrt.transition\_psi  
 PyDSTool.Toolbox.dssrt.transition\_tau  
 PyDSTool.Toolbox.event\_driven\_simulator.extract\_digraph  

PyDSTool.Toolbox.event\_driven\_simulator.head

PyDSTool.Toolbox.event\_driven\_simulator.sequences\_to\_eventlist

PyDSTool.Toolbox.event\_driven\_simulator.tail

PyDSTool.Toolbox.fracdim.PD\_E  
 PyDSTool.Toolbox.fracdim.corr\_dim  
 PyDSTool.Toolbox.fracdim.corr\_dim\_with\_progress  
 PyDSTool.Toolbox.fracdim.do\_stats  
 PyDSTool.Toolbox.fracdim.filter\_by\_radius  
 PyDSTool.Toolbox.fracdim.find\_cover  

PyDSTool.Toolbox.fracdim.find\_from\_sorted

PyDSTool.Toolbox.fracdim.find\_nhd  
 PyDSTool.Toolbox.fracdim.find\_outliers  
 PyDSTool.Toolbox.fracdim.get\_cover\_radii  
 PyDSTool.Toolbox.fracdim.get\_filtered\_ixs  
 PyDSTool.Toolbox.fracdim.get\_rV\_curves  

PyDSTool.Toolbox.fracdim.make\_secant\_fig\_dict

PyDSTool.Toolbox.fracdim.nhd  
 PyDSTool.Toolbox.fracdim.plot\_dim\_by\_thickness  
 PyDSTool.Toolbox.fracdim.plot\_radius\_distribution  
 PyDSTool.Toolbox.fracdim.plot\_thickness\_by\_dim  
 PyDSTool.Toolbox.fracdim.prep\_secant\_figure  
 PyDSTool.Toolbox.fracdim.rescatter  
 PyDSTool.Toolbox.fracdim.scatter\_histo  
 PyDSTool.Toolbox.fracdim.scatterplot\_slopes  
 PyDSTool.Toolbox.fracdim.slope\_range  
 PyDSTool.Toolbox.fracdim.sorted\_by\_radius  
 PyDSTool.Toolbox.fracdim.sorted\_by\_slope  
 PyDSTool.Toolbox.fracdim.timeseq  
 PyDSTool.Toolbox.makeSloppyModel.makeSloppyModel  
 PyDSTool.Toolbox.mechmatlib.augment\_3\_vector  
 PyDSTool.Toolbox.mechmatlib.augment\_3x3\_matrix  
 PyDSTool.Toolbox.mechmatlib.cross  
 PyDSTool.Toolbox.mechmatlib.dot  
 PyDSTool.Toolbox.mechmatlib.make\_T  
 PyDSTool.Toolbox.mechmatlib.make\_disp  
 PyDSTool.Toolbox.mechmatlib.make\_rot  
 PyDSTool.Toolbox.neuralcomp.connectWithSynapse  
 PyDSTool.Toolbox.neuralcomp.disconnectSynapse  
 PyDSTool.Toolbox.neuralcomp.makeAdaptingSynapse  
 PyDSTool.Toolbox.neuralcomp.makeBiasChannel  
 PyDSTool.Toolbox.neuralcomp.makeChannel\_halfact  
 PyDSTool.Toolbox.neuralcomp.makeChannel\_rates  
 PyDSTool.Toolbox.neuralcomp.makeDendrite  
 PyDSTool.Toolbox.neuralcomp.makeExtInputConductanceChannel  
 PyDSTool.Toolbox.neuralcomp.makeExtInputCurrentChannel  
 PyDSTool.Toolbox.neuralcomp.makeFun  
 PyDSTool.Toolbox.neuralcomp.makeNeurite  
 PyDSTool.Toolbox.neuralcomp.makeNeuronNetwork  
 PyDSTool.Toolbox.neuralcomp.makePar  
 PyDSTool.Toolbox.neuralcomp.makePointNeuron  
 PyDSTool.Toolbox.neuralcomp.makePointNeuronNetwork  
 PyDSTool.Toolbox.neuralcomp.makePowerSpec  
 PyDSTool.Toolbox.neuralcomp.makeSoma  
 PyDSTool.Toolbox.neuralcomp.makeSynapse  
 PyDSTool.Toolbox.neuralcomp.makeSynapseChannel  
 PyDSTool.Toolbox.neuro\_data.find\_internal\_extrema  

PyDSTool.Toolbox.optimizers.criterion.composite\_criteria.all

PyDSTool.Toolbox.optimizers.criterion.composite\_criteria.any

PyDSTool.Toolbox.optimizers.criterion.facilities.criterion

PyDSTool.Toolbox.optimizers.line\_search.barzilai\_borwein\_non\_monotone\_search.BarzilaiBorwein\_nonmonotone  
 PyDSTool.Toolbox.optimizers.line\_search.barzilai\_borwein\_search.BarzilaiBorwein  

PyDSTool.Toolbox.optimizers.mod\_path

PyDSTool.Toolbox.optimizers.step.conjugate\_gradient\_step.CWConjugateGradientStep

PyDSTool.Toolbox.optimizers.step.conjugate\_gradient\_step.DConjugateGradientStep

PyDSTool.Toolbox.optimizers.step.conjugate\_gradient\_step.DYConjugateGradientStep

PyDSTool.Toolbox.optimizers.step.conjugate\_gradient\_step.FRConjugateGradientStep

PyDSTool.Toolbox.optimizers.step.conjugate\_gradient\_step.FRPRPConjugateGradientStep

PyDSTool.Toolbox.optimizers.step.conjugate\_gradient\_step.HZConjugateGradientStep

PyDSTool.Toolbox.optimizers.step.conjugate\_gradient\_step.PRPConjugateGradientStep

PyDSTool.Toolbox.optimizers.test

PyDSTool.Toolbox.optimizers.testall

PyDSTool.Toolbox.phaseplane.\_filter\_consecutive

PyDSTool.Toolbox.phaseplane.\_find\_min\_pt

PyDSTool.Toolbox.phaseplane.\_newton\_step

PyDSTool.Toolbox.phaseplane.\_sample\_array\_interior

PyDSTool.Toolbox.phaseplane.\_xinf\_1D

PyDSTool.Toolbox.phaseplane.\_xinf\_ND

PyDSTool.Toolbox.phaseplane.angle\_to\_vertical  

PyDSTool.Toolbox.phaseplane.bisection

PyDSTool.Toolbox.phaseplane.check\_bounds

PyDSTool.Toolbox.phaseplane.closest\_perp\_distance\_between\_sample\_points  
 PyDSTool.Toolbox.phaseplane.closest\_perp\_distance\_between\_splines  
 PyDSTool.Toolbox.phaseplane.closest\_perp\_distance\_on\_spline  

PyDSTool.Toolbox.phaseplane.create\_test\_fn

PyDSTool.Toolbox.phaseplane.create\_test\_fn\_with\_events

PyDSTool.Toolbox.phaseplane.crop\_2D  

PyDSTool.Toolbox.phaseplane.determine\_if\_intersect

PyDSTool.Toolbox.phaseplane.filter\_NaN  

PyDSTool.Toolbox.phaseplane.filter\_close\_points

PyDSTool.Toolbox.phaseplane.find\_fixedpoints  
 PyDSTool.Toolbox.phaseplane.find\_nearest\_sample\_points\_by\_angle  
 PyDSTool.Toolbox.phaseplane.find\_nullclines  
 PyDSTool.Toolbox.phaseplane.find\_period  
 PyDSTool.Toolbox.phaseplane.find\_saddle\_manifolds  
 PyDSTool.Toolbox.phaseplane.get\_PP  
 PyDSTool.Toolbox.phaseplane.get\_orthonormal  
 PyDSTool.Toolbox.phaseplane.get\_perp  
 PyDSTool.Toolbox.phaseplane.get\_rotated  

PyDSTool.Toolbox.phaseplane.is\_counter\_clockwise

PyDSTool.Toolbox.phaseplane.is\_min\_bracket  
 PyDSTool.Toolbox.phaseplane.line\_intersection  
 PyDSTool.Toolbox.phaseplane.make\_distance\_to\_known\_line\_auxfn  
 PyDSTool.Toolbox.phaseplane.make\_distance\_to\_line\_auxfn  
 PyDSTool.Toolbox.phaseplane.make\_flow\_normal\_event  

PyDSTool.Toolbox.phaseplane.make\_vec\_at\_A\_face\_B

PyDSTool.Toolbox.phaseplane.plot\_PP\_fps  
 PyDSTool.Toolbox.phaseplane.plot\_PP\_vf  
 PyDSTool.Toolbox.phaseplane.show\_PPs  
 PyDSTool.Toolbox.prep\_boxplot.plot\_markers  
 PyDSTool.Toolbox.prep\_boxplot.prep\_boxplots  

PyDSTool.Toolbox.synthetic\_data.generate\_ball

PyDSTool.Toolbox.synthetic\_data.generate\_discspiral  
 PyDSTool.Toolbox.synthetic\_data.generate\_hypercube  
 PyDSTool.Toolbox.synthetic\_data.generate\_spiral  
 PyDSTool.Toolbox.synthetic\_data.generate\_swirl  
 PyDSTool.Toolbox.synthetic\_data.inf\_norm  
 PyDSTool.Toolbox.synthetic\_data.max\_euclidean\_distance  
 PyDSTool.Toolbox.synthetic\_data.maxs  
 PyDSTool.Toolbox.synthetic\_data.mins  
 PyDSTool.Toolbox.synthetic\_data.sup\_norm  

PyDSTool.Toolbox.syntheticdata.generate\_ball

PyDSTool.Toolbox.syntheticdata.generate\_discspiral  
 PyDSTool.Toolbox.syntheticdata.generate\_hypercube  
 PyDSTool.Toolbox.syntheticdata.generate\_spiral  
 PyDSTool.Toolbox.syntheticdata.generate\_swirl  
 PyDSTool.Toolbox.syntheticdata.inf\_norm  
 PyDSTool.Toolbox.syntheticdata.max\_euclidean\_distance  
 PyDSTool.Toolbox.syntheticdata.maxs  
 PyDSTool.Toolbox.syntheticdata.mins  
 PyDSTool.Toolbox.syntheticdata.sup\_norm  
 PyDSTool.Trajectory'.convert\_ptlabel\_events  
 PyDSTool.Trajectory'.findApproxPeriod  
 PyDSTool.Trajectory'.numeric\_to\_traj  
 PyDSTool.Trajectory'.pointset\_to\_traj  
 PyDSTool.Variable'.iscontinuous  
 PyDSTool.Variable'.isdiscrete  
 PyDSTool.Variable'.isinputcts  
 PyDSTool.Variable'.isinputdiscrete  
 PyDSTool.Variable'.isoutputcts  
 PyDSTool.Variable'.isoutputdiscrete  
 PyDSTool.Variable'.numeric\_to\_vars  
 PyDSTool.Variable'.pointset\_to\_vars  

PyDSTool.common.\_scalar\_diff

PyDSTool.common.array\_bounds\_check  
 PyDSTool.common.arraymax  
 PyDSTool.common.className  
 PyDSTool.common.compareBaseClass  
 PyDSTool.common.compareClassAndBases  
 PyDSTool.common.compareNumTypes  
 PyDSTool.common.concatStrDict  
 PyDSTool.common.copyVarDict  
 PyDSTool.common.diff  
 PyDSTool.common.diff2  
 PyDSTool.common.ensurefloat  
 PyDSTool.common.extent  
 PyDSTool.common.filteredDict  
 PyDSTool.common.getSuperClasses  
 PyDSTool.common.get\_opt  
 PyDSTool.common.idfn  
 PyDSTool.common.insertInOrder  

PyDSTool.common.intersect

PyDSTool.common.invertMap  
 PyDSTool.common.isUniqueSeq  
 PyDSTool.common.isincreasing  
 PyDSTool.common.ismonotonic  
 PyDSTool.common.linearInterp  
 PyDSTool.common.listid  
 PyDSTool.common.makeArrayIxMap  
 PyDSTool.common.makeMultilinearRegrFn  
 PyDSTool.common.makeSeqUnique  
 PyDSTool.common.makeUniqueFn  
 PyDSTool.common.make\_poly\_interpolated\_curve  
 PyDSTool.common.n\_sigdigs\_str  
 PyDSTool.common.nearest\_2n\_indices  
 PyDSTool.common.noneFn  
 PyDSTool.common.object2str  

PyDSTool.common.remain

PyDSTool.common.simple\_bisection  
 PyDSTool.common.simplifyMatrixRepr  
 PyDSTool.common.smooth\_pts  
 PyDSTool.common.sortedDictItems  
 PyDSTool.common.sortedDictKeys  
 PyDSTool.common.sortedDictLists  
 PyDSTool.common.sortedDictValues  
 PyDSTool.common.timestamp  
 PyDSTool.common.uniquePoints  
 PyDSTool.common.verify\_intbool  
 PyDSTool.common.verify\_nonneg  
 PyDSTool.common.verify\_pos  
 PyDSTool.common.verify\_values  

PyDSTool.fixedpickle.\_keep\_alive

PyDSTool.fixedpickle.\_test

PyDSTool.fixedpickle.decode\_long

PyDSTool.fixedpickle.dump  
 PyDSTool.fixedpickle.dumps  

PyDSTool.fixedpickle.encode\_long

PyDSTool.fixedpickle.load  
 PyDSTool.fixedpickle.loads  

PyDSTool.fixedpickle.whichmodule

PyDSTool.loadSession  
 PyDSTool.matplotlib\_import.plot  
 PyDSTool.matplotlib\_import.save\_fig  
 PyDSTool.parseUtils.addArgToCalls  
 PyDSTool.parseUtils.ast2shortlist  
 PyDSTool.parseUtils.ast2string  

PyDSTool.parseUtils.collect\_numbers

PyDSTool.parseUtils.convertPowers  
 PyDSTool.parseUtils.count\_sep  
 PyDSTool.parseUtils.doadd  
 PyDSTool.parseUtils.dodiv  
 PyDSTool.parseUtils.domul  
 PyDSTool.parseUtils.doneg  
 PyDSTool.parseUtils.dopower  
 PyDSTool.parseUtils.dosub  
 PyDSTool.parseUtils.ensurebare  
 PyDSTool.parseUtils.ensuredecimalconst  

PyDSTool.parseUtils.ensureints

PyDSTool.parseUtils.ensureparen  
 PyDSTool.parseUtils.ensureparen\_div  
 PyDSTool.parseUtils.findEndBrace  
 PyDSTool.parseUtils.findNumTailPos  
 PyDSTool.parseUtils.isHierarchicalName  
 PyDSTool.parseUtils.isIntegerToken  
 PyDSTool.parseUtils.isNameToken  
 PyDSTool.parseUtils.isNumericToken  
 PyDSTool.parseUtils.isToken  

PyDSTool.parseUtils.isVectorClause

PyDSTool.parseUtils.joinAsStrs  
 PyDSTool.parseUtils.joinStrs  
 PyDSTool.parseUtils.makeParList  
 PyDSTool.parseUtils.mapNames  
 PyDSTool.parseUtils.mapPowStr  
 PyDSTool.parseUtils.parseMatrixStrToDictStr  
 PyDSTool.parseUtils.proper\_match  
 PyDSTool.parseUtils.readArgs  
 PyDSTool.parseUtils.remove\_indices\_from\_range  
 PyDSTool.parseUtils.replaceCallsWithDummies  
 PyDSTool.parseUtils.replaceSep  
 PyDSTool.parseUtils.replaceSepInv  
 PyDSTool.parseUtils.replaceSepList  
 PyDSTool.parseUtils.replaceSepListInv  

PyDSTool.parseUtils.replaceSepQSpec

PyDSTool.parseUtils.replaceSepStr

PyDSTool.parseUtils.simplify  
 PyDSTool.parseUtils.simplify\_str  
 PyDSTool.parseUtils.splitargs  
 PyDSTool.parseUtils.splitastLR  
 PyDSTool.parseUtils.string2ast  
 PyDSTool.parseUtils.sym2name  

PyDSTool.parseUtils.temp\_macro\_names

PyDSTool.parseUtils.temp\_macro\_names\_inv

PyDSTool.parseUtils.toCircumflexSyntax

PyDSTool.parseUtils.toDoubleStarSyntax

PyDSTool.parseUtils.toPowSyntax  
 PyDSTool.parseUtils.trysimple  
 PyDSTool.parseUtils.wrapArgInCall  
 PyDSTool.restart  
 PyDSTool.saveSession  

PyDSTool.scipy\_ode.f1

PyDSTool.scipy\_ode.f2

PyDSTool.scipy\_ode.find\_integrator

PyDSTool.scipy\_ode.jac

PyDSTool.scipy\_ode.noneFn

PyDSTool.scipy\_ode.test1

PyDSTool.scipy\_ode.test2

PyDSTool.utils.cartesianProduct  
 PyDSTool.utils.compareList  
 PyDSTool.utils.find  
 PyDSTool.utils.findClosestArray  
 PyDSTool.utils.findClosestPointIndex  
 PyDSTool.utils.info  
 PyDSTool.utils.intersect  
 PyDSTool.utils.loadObjects  
 PyDSTool.utils.makeDataDict  
 PyDSTool.utils.makeImplicitFunc  
 PyDSTool.utils.makeMfileFunction  
 PyDSTool.utils.make\_Jac\_wrap  
 PyDSTool.utils.make\_RHS\_wrap  
 PyDSTool.utils.orderEventData  
 PyDSTool.utils.remain  
 PyDSTool.utils.saveObjects  
 PyDSTool.utils.union  
 PyDSTool.who  

## All Variables

PyDSTool.ALLOW\_THREADS  
 PyDSTool.Abs  
 PyDSTool.Acos  
 PyDSTool.Asin  
 PyDSTool.Atan  
 PyDSTool.Atan2  
 PyDSTool.BUFSIZE  
 PyDSTool.Betavariate  
 PyDSTool.CLIP  
 PyDSTool.Ceil  
 PyDSTool.Choice  
 PyDSTool.Cos  
 PyDSTool.Cosh  
 PyDSTool.Degrees  
 PyDSTool.E  
 PyDSTool.ERR\_CALL  
 PyDSTool.ERR\_DEFAULT  
 PyDSTool.ERR\_DEFAULT2  
 PyDSTool.ERR\_IGNORE  
 PyDSTool.ERR\_LOG  
 PyDSTool.ERR\_PRINT  
 PyDSTool.ERR\_RAISE  
 PyDSTool.ERR\_WARN  

PyDSTool.Events.\_1DimplicitSolveMethods

PyDSTool.Events.\_all\_complex

PyDSTool.Events.\_all\_float

PyDSTool.Events.\_all\_int

PyDSTool.Events.\_all\_numpy\_complex

PyDSTool.Events.\_all\_numpy\_float

PyDSTool.Events.\_all\_numpy\_int

PyDSTool.Events.\_complex\_types

PyDSTool.Events.\_float\_types

PyDSTool.Events.\_implicitSolveMethods

PyDSTool.Events.\_indentstr

PyDSTool.Events.\_int\_types

PyDSTool.Events.\_num\_equivtype

PyDSTool.Events.\_num\_maxmin

PyDSTool.Events.\_num\_name2equivtypes

PyDSTool.Events.\_num\_name2type

PyDSTool.Events.\_num\_type2name

PyDSTool.Events.\_num\_types

PyDSTool.Events.\_pytypefromtype

PyDSTool.Events.\_real\_types

PyDSTool.Events.\_seq\_types

PyDSTool.Exp  
 PyDSTool.Expovariate  
 PyDSTool.FLOATING\_POINT\_SUPPORT  
 PyDSTool.FPE\_DIVIDEBYZERO  
 PyDSTool.FPE\_INVALID  
 PyDSTool.FPE\_OVERFLOW  
 PyDSTool.FPE\_UNDERFLOW  
 PyDSTool.Fabs  
 PyDSTool.False\_  
 PyDSTool.Floor  
 PyDSTool.Fmod  
 PyDSTool.Frexp  

PyDSTool.FuncSpec'.API

PyDSTool.FuncSpec'.Continuous

PyDSTool.FuncSpec'.Discrete

PyDSTool.FuncSpec'.LargestInt32

PyDSTool.FuncSpec'.NAMESEP

PyDSTool.FuncSpec'.ONES

PyDSTool.FuncSpec'.ZEROS

PyDSTool.FuncSpec'.\_1DimplicitSolveMethods

PyDSTool.FuncSpec'.\_all\_complex

PyDSTool.FuncSpec'.\_all\_float

PyDSTool.FuncSpec'.\_all\_int

PyDSTool.FuncSpec'.\_all\_numpy\_complex

PyDSTool.FuncSpec'.\_all\_numpy\_float

PyDSTool.FuncSpec'.\_all\_numpy\_int

PyDSTool.FuncSpec'.\_complex\_types

PyDSTool.FuncSpec'.\_float\_types

PyDSTool.FuncSpec'.\_implicitSolveMethods

PyDSTool.FuncSpec'.\_indentstr

PyDSTool.FuncSpec'.\_int\_types

PyDSTool.FuncSpec'.\_num\_equivtype

PyDSTool.FuncSpec'.\_num\_maxmin

PyDSTool.FuncSpec'.\_num\_name2equivtypes

PyDSTool.FuncSpec'.\_num\_name2type

PyDSTool.FuncSpec'.\_num\_type2name

PyDSTool.FuncSpec'.\_num\_types

PyDSTool.FuncSpec'.\_pytypefromtype

PyDSTool.FuncSpec'.\_real\_types

PyDSTool.FuncSpec'.\_seq\_types

PyDSTool.FuncSpec'.builtinFnSigInfo

PyDSTool.FuncSpec'.builtin\_auxnames

PyDSTool.FuncSpec'.name\_chars\_RE

PyDSTool.FuncSpec'.null\_predicate

PyDSTool.FuncSpec'.num\_chars

PyDSTool.FuncSpec'.protected\_allnames

PyDSTool.FuncSpec'.protected\_auxnamesDB

PyDSTool.FuncSpec'.protected\_macronames

PyDSTool.FuncSpec'.protected\_mathnames

PyDSTool.FuncSpec'.protected\_randomnames

PyDSTool.FuncSpec'.protected\_scipynames

PyDSTool.FuncSpec'.protected\_specialfns

PyDSTool.FuncSpec'.syms

PyDSTool.FuncSpec'.targetLangs

PyDSTool.Gammavariate  
 PyDSTool.Gauss  
 PyDSTool.Generator.ADMC\_ODEsystem'.API  
 PyDSTool.Generator.ADMC\_ODEsystem'.Continuous  
 PyDSTool.Generator.ADMC\_ODEsystem'.Discrete  
 PyDSTool.Generator.ADMC\_ODEsystem'.E\_COMPUTFAIL  
 PyDSTool.Generator.ADMC\_ODEsystem'.E\_NONUNIQUETERM  
 PyDSTool.Generator.ADMC\_ODEsystem'.Inf  
 PyDSTool.Generator.ADMC\_ODEsystem'.LargestInt32  
 PyDSTool.Generator.ADMC\_ODEsystem'.NaN  
 PyDSTool.Generator.ADMC\_ODEsystem'.W\_BISECTLIMIT  
 PyDSTool.Generator.ADMC\_ODEsystem'.W\_NONTERMEVENT  
 PyDSTool.Generator.ADMC\_ODEsystem'.W\_NONTERMSTATEBD  
 PyDSTool.Generator.ADMC\_ODEsystem'.W\_TERMEVENT  
 PyDSTool.Generator.ADMC\_ODEsystem'.W\_TERMSTATEBD  
 PyDSTool.Generator.ADMC\_ODEsystem'.W\_UNCERTVAL  

PyDSTool.Generator.ADMC\_ODEsystem'.\_1DimplicitSolveMethods

PyDSTool.Generator.ADMC\_ODEsystem'.\_all\_complex

PyDSTool.Generator.ADMC\_ODEsystem'.\_all\_float

PyDSTool.Generator.ADMC\_ODEsystem'.\_all\_int

PyDSTool.Generator.ADMC\_ODEsystem'.\_all\_numpy\_complex

PyDSTool.Generator.ADMC\_ODEsystem'.\_all\_numpy\_float

PyDSTool.Generator.ADMC\_ODEsystem'.\_all\_numpy\_int

PyDSTool.Generator.ADMC\_ODEsystem'.\_complex\_types

PyDSTool.Generator.ADMC\_ODEsystem'.\_float\_types

PyDSTool.Generator.ADMC\_ODEsystem'.\_implicitSolveMethods

PyDSTool.Generator.ADMC\_ODEsystem'.\_int\_types

PyDSTool.Generator.ADMC\_ODEsystem'.\_num\_equivtype

PyDSTool.Generator.ADMC\_ODEsystem'.\_num\_maxmin

PyDSTool.Generator.ADMC\_ODEsystem'.\_num\_name2equivtypes

PyDSTool.Generator.ADMC\_ODEsystem'.\_num\_name2type

PyDSTool.Generator.ADMC\_ODEsystem'.\_num\_type2name

PyDSTool.Generator.ADMC\_ODEsystem'.\_num\_types

PyDSTool.Generator.ADMC\_ODEsystem'.\_pydstool\_path

PyDSTool.Generator.ADMC\_ODEsystem'.\_pytypefromtype

PyDSTool.Generator.ADMC\_ODEsystem'.\_real\_types

PyDSTool.Generator.ADMC\_ODEsystem'.\_seq\_types

PyDSTool.Generator.ADMC\_ODEsystem'.contained  
 PyDSTool.Generator.ADMC\_ODEsystem'.e  
 PyDSTool.Generator.ADMC\_ODEsystem'.errmessages  
 PyDSTool.Generator.ADMC\_ODEsystem'.errorfields  
 PyDSTool.Generator.ADMC\_ODEsystem'.isfinite  
 PyDSTool.Generator.ADMC\_ODEsystem'.isnan  
 PyDSTool.Generator.ADMC\_ODEsystem'.notcontained  
 PyDSTool.Generator.ADMC\_ODEsystem'.null\_predicate  
 PyDSTool.Generator.ADMC\_ODEsystem'.pi  
 PyDSTool.Generator.ADMC\_ODEsystem'.symbolMapDict  
 PyDSTool.Generator.ADMC\_ODEsystem'.targetLangs  
 PyDSTool.Generator.ADMC\_ODEsystem'.theGenSpecHelper  
 PyDSTool.Generator.ADMC\_ODEsystem'.uncertain  
 PyDSTool.Generator.ADMC\_ODEsystem'.warnfields  
 PyDSTool.Generator.ADMC\_ODEsystem'.warnmessages  
 PyDSTool.Generator.DDEsystem.HAVE\_PSYCO  

PyDSTool.Generator.DDEsystem.\_1DimplicitSolveMethods

PyDSTool.Generator.DDEsystem.\_all\_complex

PyDSTool.Generator.DDEsystem.\_all\_float

PyDSTool.Generator.DDEsystem.\_all\_int

PyDSTool.Generator.DDEsystem.\_all\_numpy\_complex

PyDSTool.Generator.DDEsystem.\_all\_numpy\_float

PyDSTool.Generator.DDEsystem.\_all\_numpy\_int

PyDSTool.Generator.DDEsystem.\_complex\_types

PyDSTool.Generator.DDEsystem.\_float\_types

PyDSTool.Generator.DDEsystem.\_implicitSolveMethods

PyDSTool.Generator.DDEsystem.\_int\_types

PyDSTool.Generator.DDEsystem.\_num\_equivtype

PyDSTool.Generator.DDEsystem.\_num\_maxmin

PyDSTool.Generator.DDEsystem.\_num\_name2equivtypes

PyDSTool.Generator.DDEsystem.\_num\_name2type

PyDSTool.Generator.DDEsystem.\_num\_type2name

PyDSTool.Generator.DDEsystem.\_num\_types

PyDSTool.Generator.DDEsystem.\_pytypefromtype

PyDSTool.Generator.DDEsystem.\_real\_types

PyDSTool.Generator.DDEsystem.\_seq\_types

PyDSTool.Generator.DDEsystem.e  
 PyDSTool.Generator.DDEsystem.pi  
 PyDSTool.Generator.Dopri\_ODEsystem'.API  
 PyDSTool.Generator.Dopri\_ODEsystem'.Continuous  
 PyDSTool.Generator.Dopri\_ODEsystem'.Discrete  
 PyDSTool.Generator.Dopri\_ODEsystem'.E\_COMPUTFAIL  
 PyDSTool.Generator.Dopri\_ODEsystem'.E\_NONUNIQUETERM  
 PyDSTool.Generator.Dopri\_ODEsystem'.Inf  
 PyDSTool.Generator.Dopri\_ODEsystem'.LargestInt32  
 PyDSTool.Generator.Dopri\_ODEsystem'.NaN  
 PyDSTool.Generator.Dopri\_ODEsystem'.W\_BISECTLIMIT  
 PyDSTool.Generator.Dopri\_ODEsystem'.W\_NONTERMEVENT  
 PyDSTool.Generator.Dopri\_ODEsystem'.W\_NONTERMSTATEBD  
 PyDSTool.Generator.Dopri\_ODEsystem'.W\_TERMEVENT  
 PyDSTool.Generator.Dopri\_ODEsystem'.W\_TERMSTATEBD  
 PyDSTool.Generator.Dopri\_ODEsystem'.W\_UNCERTVAL  

PyDSTool.Generator.Dopri\_ODEsystem'.\_1DimplicitSolveMethods

PyDSTool.Generator.Dopri\_ODEsystem'.\_\_warningregistry\_\_  

PyDSTool.Generator.Dopri\_ODEsystem'.\_all\_complex

PyDSTool.Generator.Dopri\_ODEsystem'.\_all\_float

PyDSTool.Generator.Dopri\_ODEsystem'.\_all\_int

PyDSTool.Generator.Dopri\_ODEsystem'.\_all\_numpy\_complex

PyDSTool.Generator.Dopri\_ODEsystem'.\_all\_numpy\_float

PyDSTool.Generator.Dopri\_ODEsystem'.\_all\_numpy\_int

PyDSTool.Generator.Dopri\_ODEsystem'.\_complex\_types

PyDSTool.Generator.Dopri\_ODEsystem'.\_float\_types

PyDSTool.Generator.Dopri\_ODEsystem'.\_implicitSolveMethods

PyDSTool.Generator.Dopri\_ODEsystem'.\_int\_types

PyDSTool.Generator.Dopri\_ODEsystem'.\_num\_equivtype

PyDSTool.Generator.Dopri\_ODEsystem'.\_num\_maxmin

PyDSTool.Generator.Dopri\_ODEsystem'.\_num\_name2equivtypes

PyDSTool.Generator.Dopri\_ODEsystem'.\_num\_name2type

PyDSTool.Generator.Dopri\_ODEsystem'.\_num\_type2name

PyDSTool.Generator.Dopri\_ODEsystem'.\_num\_types

PyDSTool.Generator.Dopri\_ODEsystem'.\_pydstool\_path

PyDSTool.Generator.Dopri\_ODEsystem'.\_pytypefromtype

PyDSTool.Generator.Dopri\_ODEsystem'.\_real\_types

PyDSTool.Generator.Dopri\_ODEsystem'.\_seq\_types

PyDSTool.Generator.Dopri\_ODEsystem'.contained  
 PyDSTool.Generator.Dopri\_ODEsystem'.e  
 PyDSTool.Generator.Dopri\_ODEsystem'.errmessages  
 PyDSTool.Generator.Dopri\_ODEsystem'.errorfields  
 PyDSTool.Generator.Dopri\_ODEsystem'.genDB  
 PyDSTool.Generator.Dopri\_ODEsystem'.isfinite  
 PyDSTool.Generator.Dopri\_ODEsystem'.notcontained  
 PyDSTool.Generator.Dopri\_ODEsystem'.null\_predicate  
 PyDSTool.Generator.Dopri\_ODEsystem'.pi  
 PyDSTool.Generator.Dopri\_ODEsystem'.rerr  
 PyDSTool.Generator.Dopri\_ODEsystem'.rout  
 PyDSTool.Generator.Dopri\_ODEsystem'.symbolMapDict  
 PyDSTool.Generator.Dopri\_ODEsystem'.targetLangs  
 PyDSTool.Generator.Dopri\_ODEsystem'.theGenSpecHelper  
 PyDSTool.Generator.Dopri\_ODEsystem'.uncertain  
 PyDSTool.Generator.Dopri\_ODEsystem'.warnfields  
 PyDSTool.Generator.Dopri\_ODEsystem'.warnmessages  
 PyDSTool.Generator.EmbeddedSysGen'.API  
 PyDSTool.Generator.EmbeddedSysGen'.Continuous  
 PyDSTool.Generator.EmbeddedSysGen'.Discrete  
 PyDSTool.Generator.EmbeddedSysGen'.E\_COMPUTFAIL  
 PyDSTool.Generator.EmbeddedSysGen'.E\_NONUNIQUETERM  
 PyDSTool.Generator.EmbeddedSysGen'.HAVE\_PSYCO  
 PyDSTool.Generator.EmbeddedSysGen'.Inf  
 PyDSTool.Generator.EmbeddedSysGen'.LargestInt32  
 PyDSTool.Generator.EmbeddedSysGen'.NaN  
 PyDSTool.Generator.EmbeddedSysGen'.W\_BISECTLIMIT  
 PyDSTool.Generator.EmbeddedSysGen'.W\_NONTERMEVENT  
 PyDSTool.Generator.EmbeddedSysGen'.W\_NONTERMSTATEBD  
 PyDSTool.Generator.EmbeddedSysGen'.W\_TERMEVENT  
 PyDSTool.Generator.EmbeddedSysGen'.W\_TERMSTATEBD  
 PyDSTool.Generator.EmbeddedSysGen'.W\_UNCERTVAL  

PyDSTool.Generator.EmbeddedSysGen'.\_1DimplicitSolveMethods

PyDSTool.Generator.EmbeddedSysGen'.\_all\_complex

PyDSTool.Generator.EmbeddedSysGen'.\_all\_float

PyDSTool.Generator.EmbeddedSysGen'.\_all\_int

PyDSTool.Generator.EmbeddedSysGen'.\_all\_numpy\_complex

PyDSTool.Generator.EmbeddedSysGen'.\_all\_numpy\_float

PyDSTool.Generator.EmbeddedSysGen'.\_all\_numpy\_int

PyDSTool.Generator.EmbeddedSysGen'.\_complex\_types

PyDSTool.Generator.EmbeddedSysGen'.\_float\_types

PyDSTool.Generator.EmbeddedSysGen'.\_implicitSolveMethods

PyDSTool.Generator.EmbeddedSysGen'.\_int\_types

PyDSTool.Generator.EmbeddedSysGen'.\_num\_equivtype

PyDSTool.Generator.EmbeddedSysGen'.\_num\_maxmin

PyDSTool.Generator.EmbeddedSysGen'.\_num\_name2equivtypes

PyDSTool.Generator.EmbeddedSysGen'.\_num\_name2type

PyDSTool.Generator.EmbeddedSysGen'.\_num\_type2name

PyDSTool.Generator.EmbeddedSysGen'.\_num\_types

PyDSTool.Generator.EmbeddedSysGen'.\_pytypefromtype

PyDSTool.Generator.EmbeddedSysGen'.\_real\_types

PyDSTool.Generator.EmbeddedSysGen'.\_seq\_types

PyDSTool.Generator.EmbeddedSysGen'.contained  
 PyDSTool.Generator.EmbeddedSysGen'.e  
 PyDSTool.Generator.EmbeddedSysGen'.errmessages  
 PyDSTool.Generator.EmbeddedSysGen'.errorfields  
 PyDSTool.Generator.EmbeddedSysGen'.isfinite  
 PyDSTool.Generator.EmbeddedSysGen'.notcontained  
 PyDSTool.Generator.EmbeddedSysGen'.null\_predicate  
 PyDSTool.Generator.EmbeddedSysGen'.pi  
 PyDSTool.Generator.EmbeddedSysGen'.symbolMapDict  
 PyDSTool.Generator.EmbeddedSysGen'.targetLangs  
 PyDSTool.Generator.EmbeddedSysGen'.theGenSpecHelper  
 PyDSTool.Generator.EmbeddedSysGen'.uncertain  
 PyDSTool.Generator.EmbeddedSysGen'.warnfields  
 PyDSTool.Generator.EmbeddedSysGen'.warnmessages  
 PyDSTool.Generator.Euler\_ODEsystem'.API  
 PyDSTool.Generator.Euler\_ODEsystem'.Continuous  
 PyDSTool.Generator.Euler\_ODEsystem'.Discrete  
 PyDSTool.Generator.Euler\_ODEsystem'.E\_COMPUTFAIL  
 PyDSTool.Generator.Euler\_ODEsystem'.E\_NONUNIQUETERM  
 PyDSTool.Generator.Euler\_ODEsystem'.HAVE\_PSYCO  
 PyDSTool.Generator.Euler\_ODEsystem'.Inf  
 PyDSTool.Generator.Euler\_ODEsystem'.LargestInt32  
 PyDSTool.Generator.Euler\_ODEsystem'.NaN  
 PyDSTool.Generator.Euler\_ODEsystem'.W\_BISECTLIMIT  
 PyDSTool.Generator.Euler\_ODEsystem'.W\_NONTERMEVENT  
 PyDSTool.Generator.Euler\_ODEsystem'.W\_NONTERMSTATEBD  
 PyDSTool.Generator.Euler\_ODEsystem'.W\_TERMEVENT  
 PyDSTool.Generator.Euler\_ODEsystem'.W\_TERMSTATEBD  
 PyDSTool.Generator.Euler\_ODEsystem'.W\_UNCERTVAL  

PyDSTool.Generator.Euler\_ODEsystem'.\_1DimplicitSolveMethods

PyDSTool.Generator.Euler\_ODEsystem'.\_all\_complex

PyDSTool.Generator.Euler\_ODEsystem'.\_all\_float

PyDSTool.Generator.Euler\_ODEsystem'.\_all\_int

PyDSTool.Generator.Euler\_ODEsystem'.\_all\_numpy\_complex

PyDSTool.Generator.Euler\_ODEsystem'.\_all\_numpy\_float

PyDSTool.Generator.Euler\_ODEsystem'.\_all\_numpy\_int

PyDSTool.Generator.Euler\_ODEsystem'.\_complex\_types

PyDSTool.Generator.Euler\_ODEsystem'.\_float\_types

PyDSTool.Generator.Euler\_ODEsystem'.\_implicitSolveMethods

PyDSTool.Generator.Euler\_ODEsystem'.\_int\_types

PyDSTool.Generator.Euler\_ODEsystem'.\_num\_equivtype

PyDSTool.Generator.Euler\_ODEsystem'.\_num\_maxmin

PyDSTool.Generator.Euler\_ODEsystem'.\_num\_name2equivtypes

PyDSTool.Generator.Euler\_ODEsystem'.\_num\_name2type

PyDSTool.Generator.Euler\_ODEsystem'.\_num\_type2name

PyDSTool.Generator.Euler\_ODEsystem'.\_num\_types

PyDSTool.Generator.Euler\_ODEsystem'.\_pytypefromtype

PyDSTool.Generator.Euler\_ODEsystem'.\_real\_types

PyDSTool.Generator.Euler\_ODEsystem'.\_seq\_types

PyDSTool.Generator.Euler\_ODEsystem'.contained  
 PyDSTool.Generator.Euler\_ODEsystem'.e  
 PyDSTool.Generator.Euler\_ODEsystem'.errmessages  
 PyDSTool.Generator.Euler\_ODEsystem'.errorfields  
 PyDSTool.Generator.Euler\_ODEsystem'.isfinite  
 PyDSTool.Generator.Euler\_ODEsystem'.less\_equal  
 PyDSTool.Generator.Euler\_ODEsystem'.notcontained  
 PyDSTool.Generator.Euler\_ODEsystem'.null\_predicate  
 PyDSTool.Generator.Euler\_ODEsystem'.pi  
 PyDSTool.Generator.Euler\_ODEsystem'.sign  
 PyDSTool.Generator.Euler\_ODEsystem'.symbolMapDict  
 PyDSTool.Generator.Euler\_ODEsystem'.targetLangs  
 PyDSTool.Generator.Euler\_ODEsystem'.theGenSpecHelper  
 PyDSTool.Generator.Euler\_ODEsystem'.uncertain  
 PyDSTool.Generator.Euler\_ODEsystem'.warnfields  
 PyDSTool.Generator.Euler\_ODEsystem'.warnmessages  
 PyDSTool.Generator.ExplicitFnGen'.API  
 PyDSTool.Generator.ExplicitFnGen'.Continuous  
 PyDSTool.Generator.ExplicitFnGen'.Discrete  
 PyDSTool.Generator.ExplicitFnGen'.E\_COMPUTFAIL  
 PyDSTool.Generator.ExplicitFnGen'.E\_NONUNIQUETERM  
 PyDSTool.Generator.ExplicitFnGen'.HAVE\_PSYCO  
 PyDSTool.Generator.ExplicitFnGen'.Inf  
 PyDSTool.Generator.ExplicitFnGen'.LargestInt32  
 PyDSTool.Generator.ExplicitFnGen'.NaN  
 PyDSTool.Generator.ExplicitFnGen'.W\_BISECTLIMIT  
 PyDSTool.Generator.ExplicitFnGen'.W\_NONTERMEVENT  
 PyDSTool.Generator.ExplicitFnGen'.W\_NONTERMSTATEBD  
 PyDSTool.Generator.ExplicitFnGen'.W\_TERMEVENT  
 PyDSTool.Generator.ExplicitFnGen'.W\_TERMSTATEBD  
 PyDSTool.Generator.ExplicitFnGen'.W\_UNCERTVAL  

PyDSTool.Generator.ExplicitFnGen'.\_1DimplicitSolveMethods

PyDSTool.Generator.ExplicitFnGen'.\_all\_complex

PyDSTool.Generator.ExplicitFnGen'.\_all\_float

PyDSTool.Generator.ExplicitFnGen'.\_all\_int

PyDSTool.Generator.ExplicitFnGen'.\_all\_numpy\_complex

PyDSTool.Generator.ExplicitFnGen'.\_all\_numpy\_float

PyDSTool.Generator.ExplicitFnGen'.\_all\_numpy\_int

PyDSTool.Generator.ExplicitFnGen'.\_complex\_types

PyDSTool.Generator.ExplicitFnGen'.\_float\_types

PyDSTool.Generator.ExplicitFnGen'.\_implicitSolveMethods

PyDSTool.Generator.ExplicitFnGen'.\_int\_types

PyDSTool.Generator.ExplicitFnGen'.\_num\_equivtype

PyDSTool.Generator.ExplicitFnGen'.\_num\_maxmin

PyDSTool.Generator.ExplicitFnGen'.\_num\_name2equivtypes

PyDSTool.Generator.ExplicitFnGen'.\_num\_name2type

PyDSTool.Generator.ExplicitFnGen'.\_num\_type2name

PyDSTool.Generator.ExplicitFnGen'.\_num\_types

PyDSTool.Generator.ExplicitFnGen'.\_pytypefromtype

PyDSTool.Generator.ExplicitFnGen'.\_real\_types

PyDSTool.Generator.ExplicitFnGen'.\_seq\_types

PyDSTool.Generator.ExplicitFnGen'.contained  
 PyDSTool.Generator.ExplicitFnGen'.e  
 PyDSTool.Generator.ExplicitFnGen'.errmessages  
 PyDSTool.Generator.ExplicitFnGen'.errorfields  
 PyDSTool.Generator.ExplicitFnGen'.isfinite  
 PyDSTool.Generator.ExplicitFnGen'.notcontained  
 PyDSTool.Generator.ExplicitFnGen'.null\_predicate  
 PyDSTool.Generator.ExplicitFnGen'.pi  
 PyDSTool.Generator.ExplicitFnGen'.symbolMapDict  
 PyDSTool.Generator.ExplicitFnGen'.targetLangs  
 PyDSTool.Generator.ExplicitFnGen'.theGenSpecHelper  
 PyDSTool.Generator.ExplicitFnGen'.uncertain  
 PyDSTool.Generator.ExplicitFnGen'.warnfields  
 PyDSTool.Generator.ExplicitFnGen'.warnmessages  
 PyDSTool.Generator.ExtrapolateTable'.API  
 PyDSTool.Generator.ExtrapolateTable'.Continuous  
 PyDSTool.Generator.ExtrapolateTable'.Discrete  
 PyDSTool.Generator.ExtrapolateTable'.E\_COMPUTFAIL  
 PyDSTool.Generator.ExtrapolateTable'.E\_NONUNIQUETERM  
 PyDSTool.Generator.ExtrapolateTable'.Inf  
 PyDSTool.Generator.ExtrapolateTable'.LargestInt32  
 PyDSTool.Generator.ExtrapolateTable'.NaN  
 PyDSTool.Generator.ExtrapolateTable'.W\_BISECTLIMIT  
 PyDSTool.Generator.ExtrapolateTable'.W\_NONTERMEVENT  
 PyDSTool.Generator.ExtrapolateTable'.W\_NONTERMSTATEBD  
 PyDSTool.Generator.ExtrapolateTable'.W\_TERMEVENT  
 PyDSTool.Generator.ExtrapolateTable'.W\_TERMSTATEBD  
 PyDSTool.Generator.ExtrapolateTable'.W\_UNCERTVAL  

PyDSTool.Generator.ExtrapolateTable'.\_1DimplicitSolveMethods

PyDSTool.Generator.ExtrapolateTable'.\_all\_complex

PyDSTool.Generator.ExtrapolateTable'.\_all\_float

PyDSTool.Generator.ExtrapolateTable'.\_all\_int

PyDSTool.Generator.ExtrapolateTable'.\_all\_numpy\_complex

PyDSTool.Generator.ExtrapolateTable'.\_all\_numpy\_float

PyDSTool.Generator.ExtrapolateTable'.\_all\_numpy\_int

PyDSTool.Generator.ExtrapolateTable'.\_complex\_types

PyDSTool.Generator.ExtrapolateTable'.\_float\_types

PyDSTool.Generator.ExtrapolateTable'.\_implicitSolveMethods

PyDSTool.Generator.ExtrapolateTable'.\_int\_types

PyDSTool.Generator.ExtrapolateTable'.\_num\_equivtype

PyDSTool.Generator.ExtrapolateTable'.\_num\_maxmin

PyDSTool.Generator.ExtrapolateTable'.\_num\_name2equivtypes

PyDSTool.Generator.ExtrapolateTable'.\_num\_name2type

PyDSTool.Generator.ExtrapolateTable'.\_num\_type2name

PyDSTool.Generator.ExtrapolateTable'.\_num\_types

PyDSTool.Generator.ExtrapolateTable'.\_pytypefromtype

PyDSTool.Generator.ExtrapolateTable'.\_real\_types

PyDSTool.Generator.ExtrapolateTable'.\_seq\_types

PyDSTool.Generator.ExtrapolateTable'.contained  
 PyDSTool.Generator.ExtrapolateTable'.e  
 PyDSTool.Generator.ExtrapolateTable'.errmessages  
 PyDSTool.Generator.ExtrapolateTable'.errorfields  
 PyDSTool.Generator.ExtrapolateTable'.isfinite  
 PyDSTool.Generator.ExtrapolateTable'.notcontained  
 PyDSTool.Generator.ExtrapolateTable'.null\_predicate  
 PyDSTool.Generator.ExtrapolateTable'.pi  
 PyDSTool.Generator.ExtrapolateTable'.symbolMapDict  
 PyDSTool.Generator.ExtrapolateTable'.targetLangs  
 PyDSTool.Generator.ExtrapolateTable'.theGenSpecHelper  
 PyDSTool.Generator.ExtrapolateTable'.uncertain  
 PyDSTool.Generator.ExtrapolateTable'.warnfields  
 PyDSTool.Generator.ExtrapolateTable'.warnmessages  
 PyDSTool.Generator.ImplicitFnGen'.API  
 PyDSTool.Generator.ImplicitFnGen'.Continuous  
 PyDSTool.Generator.ImplicitFnGen'.Discrete  
 PyDSTool.Generator.ImplicitFnGen'.E\_COMPUTFAIL  
 PyDSTool.Generator.ImplicitFnGen'.E\_NONUNIQUETERM  
 PyDSTool.Generator.ImplicitFnGen'.HAVE\_PSYCO  
 PyDSTool.Generator.ImplicitFnGen'.Inf  
 PyDSTool.Generator.ImplicitFnGen'.LargestInt32  
 PyDSTool.Generator.ImplicitFnGen'.NaN  
 PyDSTool.Generator.ImplicitFnGen'.W\_BISECTLIMIT  
 PyDSTool.Generator.ImplicitFnGen'.W\_NONTERMEVENT  
 PyDSTool.Generator.ImplicitFnGen'.W\_NONTERMSTATEBD  
 PyDSTool.Generator.ImplicitFnGen'.W\_TERMEVENT  
 PyDSTool.Generator.ImplicitFnGen'.W\_TERMSTATEBD  
 PyDSTool.Generator.ImplicitFnGen'.W\_UNCERTVAL  

PyDSTool.Generator.ImplicitFnGen'.\_1DimplicitSolveMethods

PyDSTool.Generator.ImplicitFnGen'.\_all\_complex

PyDSTool.Generator.ImplicitFnGen'.\_all\_float

PyDSTool.Generator.ImplicitFnGen'.\_all\_int

PyDSTool.Generator.ImplicitFnGen'.\_all\_numpy\_complex

PyDSTool.Generator.ImplicitFnGen'.\_all\_numpy\_float

PyDSTool.Generator.ImplicitFnGen'.\_all\_numpy\_int

PyDSTool.Generator.ImplicitFnGen'.\_complex\_types

PyDSTool.Generator.ImplicitFnGen'.\_float\_types

PyDSTool.Generator.ImplicitFnGen'.\_implicitSolveMethods

PyDSTool.Generator.ImplicitFnGen'.\_int\_types

PyDSTool.Generator.ImplicitFnGen'.\_num\_equivtype

PyDSTool.Generator.ImplicitFnGen'.\_num\_maxmin

PyDSTool.Generator.ImplicitFnGen'.\_num\_name2equivtypes

PyDSTool.Generator.ImplicitFnGen'.\_num\_name2type

PyDSTool.Generator.ImplicitFnGen'.\_num\_type2name

PyDSTool.Generator.ImplicitFnGen'.\_num\_types

PyDSTool.Generator.ImplicitFnGen'.\_pytypefromtype

PyDSTool.Generator.ImplicitFnGen'.\_real\_types

PyDSTool.Generator.ImplicitFnGen'.\_seq\_types

PyDSTool.Generator.ImplicitFnGen'.contained  
 PyDSTool.Generator.ImplicitFnGen'.e  
 PyDSTool.Generator.ImplicitFnGen'.errmessages  
 PyDSTool.Generator.ImplicitFnGen'.errorfields  
 PyDSTool.Generator.ImplicitFnGen'.isfinite  
 PyDSTool.Generator.ImplicitFnGen'.notcontained  
 PyDSTool.Generator.ImplicitFnGen'.null\_predicate  
 PyDSTool.Generator.ImplicitFnGen'.pi  
 PyDSTool.Generator.ImplicitFnGen'.symbolMapDict  
 PyDSTool.Generator.ImplicitFnGen'.targetLangs  
 PyDSTool.Generator.ImplicitFnGen'.theGenSpecHelper  
 PyDSTool.Generator.ImplicitFnGen'.uncertain  
 PyDSTool.Generator.ImplicitFnGen'.warnfields  
 PyDSTool.Generator.ImplicitFnGen'.warnmessages  
 PyDSTool.Generator.InterpolateTable'.API  
 PyDSTool.Generator.InterpolateTable'.Continuous  
 PyDSTool.Generator.InterpolateTable'.Discrete  
 PyDSTool.Generator.InterpolateTable'.E\_COMPUTFAIL  
 PyDSTool.Generator.InterpolateTable'.E\_NONUNIQUETERM  
 PyDSTool.Generator.InterpolateTable'.Inf  
 PyDSTool.Generator.InterpolateTable'.LargestInt32  
 PyDSTool.Generator.InterpolateTable'.NaN  
 PyDSTool.Generator.InterpolateTable'.W\_BISECTLIMIT  
 PyDSTool.Generator.InterpolateTable'.W\_NONTERMEVENT  
 PyDSTool.Generator.InterpolateTable'.W\_NONTERMSTATEBD  
 PyDSTool.Generator.InterpolateTable'.W\_TERMEVENT  
 PyDSTool.Generator.InterpolateTable'.W\_TERMSTATEBD  
 PyDSTool.Generator.InterpolateTable'.W\_UNCERTVAL  

PyDSTool.Generator.InterpolateTable'.\_1DimplicitSolveMethods

PyDSTool.Generator.InterpolateTable'.\_all\_complex

PyDSTool.Generator.InterpolateTable'.\_all\_float

PyDSTool.Generator.InterpolateTable'.\_all\_int

PyDSTool.Generator.InterpolateTable'.\_all\_numpy\_complex

PyDSTool.Generator.InterpolateTable'.\_all\_numpy\_float

PyDSTool.Generator.InterpolateTable'.\_all\_numpy\_int

PyDSTool.Generator.InterpolateTable'.\_complex\_types

PyDSTool.Generator.InterpolateTable'.\_float\_types

PyDSTool.Generator.InterpolateTable'.\_implicitSolveMethods

PyDSTool.Generator.InterpolateTable'.\_int\_types

PyDSTool.Generator.InterpolateTable'.\_num\_equivtype

PyDSTool.Generator.InterpolateTable'.\_num\_maxmin

PyDSTool.Generator.InterpolateTable'.\_num\_name2equivtypes

PyDSTool.Generator.InterpolateTable'.\_num\_name2type

PyDSTool.Generator.InterpolateTable'.\_num\_type2name

PyDSTool.Generator.InterpolateTable'.\_num\_types

PyDSTool.Generator.InterpolateTable'.\_pytypefromtype

PyDSTool.Generator.InterpolateTable'.\_real\_types

PyDSTool.Generator.InterpolateTable'.\_seq\_types

PyDSTool.Generator.InterpolateTable'.contained  
 PyDSTool.Generator.InterpolateTable'.e  
 PyDSTool.Generator.InterpolateTable'.errmessages  
 PyDSTool.Generator.InterpolateTable'.errorfields  
 PyDSTool.Generator.InterpolateTable'.isfinite  
 PyDSTool.Generator.InterpolateTable'.notcontained  
 PyDSTool.Generator.InterpolateTable'.null\_predicate  
 PyDSTool.Generator.InterpolateTable'.pi  
 PyDSTool.Generator.InterpolateTable'.symbolMapDict  
 PyDSTool.Generator.InterpolateTable'.targetLangs  
 PyDSTool.Generator.InterpolateTable'.theGenSpecHelper  
 PyDSTool.Generator.InterpolateTable'.uncertain  
 PyDSTool.Generator.InterpolateTable'.warnfields  
 PyDSTool.Generator.InterpolateTable'.warnmessages  
 PyDSTool.Generator.LookupTable'.API  
 PyDSTool.Generator.LookupTable'.Continuous  
 PyDSTool.Generator.LookupTable'.Discrete  
 PyDSTool.Generator.LookupTable'.E\_COMPUTFAIL  
 PyDSTool.Generator.LookupTable'.E\_NONUNIQUETERM  
 PyDSTool.Generator.LookupTable'.Inf  
 PyDSTool.Generator.LookupTable'.LargestInt32  
 PyDSTool.Generator.LookupTable'.NaN  
 PyDSTool.Generator.LookupTable'.W\_BISECTLIMIT  
 PyDSTool.Generator.LookupTable'.W\_NONTERMEVENT  
 PyDSTool.Generator.LookupTable'.W\_NONTERMSTATEBD  
 PyDSTool.Generator.LookupTable'.W\_TERMEVENT  
 PyDSTool.Generator.LookupTable'.W\_TERMSTATEBD  
 PyDSTool.Generator.LookupTable'.W\_UNCERTVAL  

PyDSTool.Generator.LookupTable'.\_1DimplicitSolveMethods

PyDSTool.Generator.LookupTable'.\_all\_complex

PyDSTool.Generator.LookupTable'.\_all\_float

PyDSTool.Generator.LookupTable'.\_all\_int

PyDSTool.Generator.LookupTable'.\_all\_numpy\_complex

PyDSTool.Generator.LookupTable'.\_all\_numpy\_float

PyDSTool.Generator.LookupTable'.\_all\_numpy\_int

PyDSTool.Generator.LookupTable'.\_complex\_types

PyDSTool.Generator.LookupTable'.\_float\_types

PyDSTool.Generator.LookupTable'.\_implicitSolveMethods

PyDSTool.Generator.LookupTable'.\_int\_types

PyDSTool.Generator.LookupTable'.\_num\_equivtype

PyDSTool.Generator.LookupTable'.\_num\_maxmin

PyDSTool.Generator.LookupTable'.\_num\_name2equivtypes

PyDSTool.Generator.LookupTable'.\_num\_name2type

PyDSTool.Generator.LookupTable'.\_num\_type2name

PyDSTool.Generator.LookupTable'.\_num\_types

PyDSTool.Generator.LookupTable'.\_pytypefromtype

PyDSTool.Generator.LookupTable'.\_real\_types

PyDSTool.Generator.LookupTable'.\_seq\_types

PyDSTool.Generator.LookupTable'.contained  
 PyDSTool.Generator.LookupTable'.e  
 PyDSTool.Generator.LookupTable'.errmessages  
 PyDSTool.Generator.LookupTable'.errorfields  
 PyDSTool.Generator.LookupTable'.isfinite  
 PyDSTool.Generator.LookupTable'.notcontained  
 PyDSTool.Generator.LookupTable'.null\_predicate  
 PyDSTool.Generator.LookupTable'.pi  
 PyDSTool.Generator.LookupTable'.symbolMapDict  
 PyDSTool.Generator.LookupTable'.targetLangs  
 PyDSTool.Generator.LookupTable'.theGenSpecHelper  
 PyDSTool.Generator.LookupTable'.uncertain  
 PyDSTool.Generator.LookupTable'.warnfields  
 PyDSTool.Generator.LookupTable'.warnmessages  
 PyDSTool.Generator.MapSystem'.API  
 PyDSTool.Generator.MapSystem'.Continuous  
 PyDSTool.Generator.MapSystem'.Discrete  
 PyDSTool.Generator.MapSystem'.E\_COMPUTFAIL  
 PyDSTool.Generator.MapSystem'.E\_NONUNIQUETERM  
 PyDSTool.Generator.MapSystem'.HAVE\_PSYCO  
 PyDSTool.Generator.MapSystem'.Inf  
 PyDSTool.Generator.MapSystem'.LargestInt32  
 PyDSTool.Generator.MapSystem'.NaN  
 PyDSTool.Generator.MapSystem'.W\_BISECTLIMIT  
 PyDSTool.Generator.MapSystem'.W\_NONTERMEVENT  
 PyDSTool.Generator.MapSystem'.W\_NONTERMSTATEBD  
 PyDSTool.Generator.MapSystem'.W\_TERMEVENT  
 PyDSTool.Generator.MapSystem'.W\_TERMSTATEBD  
 PyDSTool.Generator.MapSystem'.W\_UNCERTVAL  

PyDSTool.Generator.MapSystem'.\_1DimplicitSolveMethods

PyDSTool.Generator.MapSystem'.\_all\_complex

PyDSTool.Generator.MapSystem'.\_all\_float

PyDSTool.Generator.MapSystem'.\_all\_int

PyDSTool.Generator.MapSystem'.\_all\_numpy\_complex

PyDSTool.Generator.MapSystem'.\_all\_numpy\_float

PyDSTool.Generator.MapSystem'.\_all\_numpy\_int

PyDSTool.Generator.MapSystem'.\_complex\_types

PyDSTool.Generator.MapSystem'.\_float\_types

PyDSTool.Generator.MapSystem'.\_implicitSolveMethods

PyDSTool.Generator.MapSystem'.\_int\_types

PyDSTool.Generator.MapSystem'.\_num\_equivtype

PyDSTool.Generator.MapSystem'.\_num\_maxmin

PyDSTool.Generator.MapSystem'.\_num\_name2equivtypes

PyDSTool.Generator.MapSystem'.\_num\_name2type

PyDSTool.Generator.MapSystem'.\_num\_type2name

PyDSTool.Generator.MapSystem'.\_num\_types

PyDSTool.Generator.MapSystem'.\_pytypefromtype

PyDSTool.Generator.MapSystem'.\_real\_types

PyDSTool.Generator.MapSystem'.\_seq\_types

PyDSTool.Generator.MapSystem'.contained  
 PyDSTool.Generator.MapSystem'.e  
 PyDSTool.Generator.MapSystem'.errmessages  
 PyDSTool.Generator.MapSystem'.errorfields  
 PyDSTool.Generator.MapSystem'.isfinite  
 PyDSTool.Generator.MapSystem'.notcontained  
 PyDSTool.Generator.MapSystem'.null\_predicate  
 PyDSTool.Generator.MapSystem'.pi  
 PyDSTool.Generator.MapSystem'.symbolMapDict  
 PyDSTool.Generator.MapSystem'.targetLangs  
 PyDSTool.Generator.MapSystem'.theGenSpecHelper  
 PyDSTool.Generator.MapSystem'.uncertain  
 PyDSTool.Generator.MapSystem'.warnfields  
 PyDSTool.Generator.MapSystem'.warnmessages  
 PyDSTool.Generator.ODEsystem'.API  
 PyDSTool.Generator.ODEsystem'.Continuous  
 PyDSTool.Generator.ODEsystem'.Discrete  
 PyDSTool.Generator.ODEsystem'.E\_COMPUTFAIL  
 PyDSTool.Generator.ODEsystem'.E\_NONUNIQUETERM  
 PyDSTool.Generator.ODEsystem'.HAVE\_PSYCO  
 PyDSTool.Generator.ODEsystem'.Inf  
 PyDSTool.Generator.ODEsystem'.LargestInt32  
 PyDSTool.Generator.ODEsystem'.NaN  
 PyDSTool.Generator.ODEsystem'.W\_BISECTLIMIT  
 PyDSTool.Generator.ODEsystem'.W\_NONTERMEVENT  
 PyDSTool.Generator.ODEsystem'.W\_NONTERMSTATEBD  
 PyDSTool.Generator.ODEsystem'.W\_TERMEVENT  
 PyDSTool.Generator.ODEsystem'.W\_TERMSTATEBD  
 PyDSTool.Generator.ODEsystem'.W\_UNCERTVAL  

PyDSTool.Generator.ODEsystem'.\_1DimplicitSolveMethods

PyDSTool.Generator.ODEsystem'.\_all\_complex

PyDSTool.Generator.ODEsystem'.\_all\_float

PyDSTool.Generator.ODEsystem'.\_all\_int

PyDSTool.Generator.ODEsystem'.\_all\_numpy\_complex

PyDSTool.Generator.ODEsystem'.\_all\_numpy\_float

PyDSTool.Generator.ODEsystem'.\_all\_numpy\_int

PyDSTool.Generator.ODEsystem'.\_complex\_types

PyDSTool.Generator.ODEsystem'.\_float\_types

PyDSTool.Generator.ODEsystem'.\_implicitSolveMethods

PyDSTool.Generator.ODEsystem'.\_int\_types

PyDSTool.Generator.ODEsystem'.\_num\_equivtype

PyDSTool.Generator.ODEsystem'.\_num\_maxmin

PyDSTool.Generator.ODEsystem'.\_num\_name2equivtypes

PyDSTool.Generator.ODEsystem'.\_num\_name2type

PyDSTool.Generator.ODEsystem'.\_num\_type2name

PyDSTool.Generator.ODEsystem'.\_num\_types

PyDSTool.Generator.ODEsystem'.\_pytypefromtype

PyDSTool.Generator.ODEsystem'.\_real\_types

PyDSTool.Generator.ODEsystem'.\_seq\_types

PyDSTool.Generator.ODEsystem'.contained  
 PyDSTool.Generator.ODEsystem'.e  
 PyDSTool.Generator.ODEsystem'.errmessages  
 PyDSTool.Generator.ODEsystem'.errorfields  
 PyDSTool.Generator.ODEsystem'.isfinite  
 PyDSTool.Generator.ODEsystem'.notcontained  
 PyDSTool.Generator.ODEsystem'.null\_predicate  
 PyDSTool.Generator.ODEsystem'.pi  
 PyDSTool.Generator.ODEsystem'.targetLangs  
 PyDSTool.Generator.ODEsystem'.theGenSpecHelper  
 PyDSTool.Generator.ODEsystem'.uncertain  
 PyDSTool.Generator.ODEsystem'.warnfields  
 PyDSTool.Generator.ODEsystem'.warnmessages  
 PyDSTool.Generator.Radau\_ODEsystem'.API  
 PyDSTool.Generator.Radau\_ODEsystem'.Continuous  
 PyDSTool.Generator.Radau\_ODEsystem'.Discrete  
 PyDSTool.Generator.Radau\_ODEsystem'.E\_COMPUTFAIL  
 PyDSTool.Generator.Radau\_ODEsystem'.E\_NONUNIQUETERM  
 PyDSTool.Generator.Radau\_ODEsystem'.Inf  
 PyDSTool.Generator.Radau\_ODEsystem'.LargestInt32  
 PyDSTool.Generator.Radau\_ODEsystem'.NaN  
 PyDSTool.Generator.Radau\_ODEsystem'.W\_BISECTLIMIT  
 PyDSTool.Generator.Radau\_ODEsystem'.W\_NONTERMEVENT  
 PyDSTool.Generator.Radau\_ODEsystem'.W\_NONTERMSTATEBD  
 PyDSTool.Generator.Radau\_ODEsystem'.W\_TERMEVENT  
 PyDSTool.Generator.Radau\_ODEsystem'.W\_TERMSTATEBD  
 PyDSTool.Generator.Radau\_ODEsystem'.W\_UNCERTVAL  

PyDSTool.Generator.Radau\_ODEsystem'.\_1DimplicitSolveMethods

PyDSTool.Generator.Radau\_ODEsystem'.\_all\_complex

PyDSTool.Generator.Radau\_ODEsystem'.\_all\_float

PyDSTool.Generator.Radau\_ODEsystem'.\_all\_int

PyDSTool.Generator.Radau\_ODEsystem'.\_all\_numpy\_complex

PyDSTool.Generator.Radau\_ODEsystem'.\_all\_numpy\_float

PyDSTool.Generator.Radau\_ODEsystem'.\_all\_numpy\_int

PyDSTool.Generator.Radau\_ODEsystem'.\_complex\_types

PyDSTool.Generator.Radau\_ODEsystem'.\_float\_types

PyDSTool.Generator.Radau\_ODEsystem'.\_implicitSolveMethods

PyDSTool.Generator.Radau\_ODEsystem'.\_int\_types

PyDSTool.Generator.Radau\_ODEsystem'.\_num\_equivtype

PyDSTool.Generator.Radau\_ODEsystem'.\_num\_maxmin

PyDSTool.Generator.Radau\_ODEsystem'.\_num\_name2equivtypes

PyDSTool.Generator.Radau\_ODEsystem'.\_num\_name2type

PyDSTool.Generator.Radau\_ODEsystem'.\_num\_type2name

PyDSTool.Generator.Radau\_ODEsystem'.\_num\_types

PyDSTool.Generator.Radau\_ODEsystem'.\_pydstool\_path

PyDSTool.Generator.Radau\_ODEsystem'.\_pytypefromtype

PyDSTool.Generator.Radau\_ODEsystem'.\_real\_types

PyDSTool.Generator.Radau\_ODEsystem'.\_seq\_types

PyDSTool.Generator.Radau\_ODEsystem'.contained  
 PyDSTool.Generator.Radau\_ODEsystem'.e  
 PyDSTool.Generator.Radau\_ODEsystem'.errmessages  
 PyDSTool.Generator.Radau\_ODEsystem'.errorfields  
 PyDSTool.Generator.Radau\_ODEsystem'.genDB  
 PyDSTool.Generator.Radau\_ODEsystem'.isfinite  
 PyDSTool.Generator.Radau\_ODEsystem'.notcontained  
 PyDSTool.Generator.Radau\_ODEsystem'.null\_predicate  
 PyDSTool.Generator.Radau\_ODEsystem'.pi  
 PyDSTool.Generator.Radau\_ODEsystem'.rerr  
 PyDSTool.Generator.Radau\_ODEsystem'.rout  
 PyDSTool.Generator.Radau\_ODEsystem'.symbolMapDict  
 PyDSTool.Generator.Radau\_ODEsystem'.targetLangs  
 PyDSTool.Generator.Radau\_ODEsystem'.theGenSpecHelper  
 PyDSTool.Generator.Radau\_ODEsystem'.uncertain  
 PyDSTool.Generator.Radau\_ODEsystem'.warnfields  
 PyDSTool.Generator.Radau\_ODEsystem'.warnmessages  
 PyDSTool.Generator.Vode\_ODEsystem'.API  
 PyDSTool.Generator.Vode\_ODEsystem'.Continuous  
 PyDSTool.Generator.Vode\_ODEsystem'.Discrete  
 PyDSTool.Generator.Vode\_ODEsystem'.E\_COMPUTFAIL  
 PyDSTool.Generator.Vode\_ODEsystem'.E\_NONUNIQUETERM  
 PyDSTool.Generator.Vode\_ODEsystem'.HAVE\_PSYCO  
 PyDSTool.Generator.Vode\_ODEsystem'.Inf  
 PyDSTool.Generator.Vode\_ODEsystem'.LargestInt32  
 PyDSTool.Generator.Vode\_ODEsystem'.NaN  
 PyDSTool.Generator.Vode\_ODEsystem'.W\_BISECTLIMIT  
 PyDSTool.Generator.Vode\_ODEsystem'.W\_NONTERMEVENT  
 PyDSTool.Generator.Vode\_ODEsystem'.W\_NONTERMSTATEBD  
 PyDSTool.Generator.Vode\_ODEsystem'.W\_TERMEVENT  
 PyDSTool.Generator.Vode\_ODEsystem'.W\_TERMSTATEBD  
 PyDSTool.Generator.Vode\_ODEsystem'.W\_UNCERTVAL  

PyDSTool.Generator.Vode\_ODEsystem'.\_1DimplicitSolveMethods

PyDSTool.Generator.Vode\_ODEsystem'.\_all\_complex

PyDSTool.Generator.Vode\_ODEsystem'.\_all\_float

PyDSTool.Generator.Vode\_ODEsystem'.\_all\_int

PyDSTool.Generator.Vode\_ODEsystem'.\_all\_numpy\_complex

PyDSTool.Generator.Vode\_ODEsystem'.\_all\_numpy\_float

PyDSTool.Generator.Vode\_ODEsystem'.\_all\_numpy\_int

PyDSTool.Generator.Vode\_ODEsystem'.\_complex\_types

PyDSTool.Generator.Vode\_ODEsystem'.\_float\_types

PyDSTool.Generator.Vode\_ODEsystem'.\_implicitSolveMethods

PyDSTool.Generator.Vode\_ODEsystem'.\_int\_types

PyDSTool.Generator.Vode\_ODEsystem'.\_num\_equivtype

PyDSTool.Generator.Vode\_ODEsystem'.\_num\_maxmin

PyDSTool.Generator.Vode\_ODEsystem'.\_num\_name2equivtypes

PyDSTool.Generator.Vode\_ODEsystem'.\_num\_name2type

PyDSTool.Generator.Vode\_ODEsystem'.\_num\_type2name

PyDSTool.Generator.Vode\_ODEsystem'.\_num\_types

PyDSTool.Generator.Vode\_ODEsystem'.\_pytypefromtype

PyDSTool.Generator.Vode\_ODEsystem'.\_real\_types

PyDSTool.Generator.Vode\_ODEsystem'.\_seq\_types

PyDSTool.Generator.Vode\_ODEsystem'.contained  
 PyDSTool.Generator.Vode\_ODEsystem'.e  
 PyDSTool.Generator.Vode\_ODEsystem'.errmessages  
 PyDSTool.Generator.Vode\_ODEsystem'.errorfields  
 PyDSTool.Generator.Vode\_ODEsystem'.isfinite  
 PyDSTool.Generator.Vode\_ODEsystem'.less\_equal  
 PyDSTool.Generator.Vode\_ODEsystem'.notcontained  
 PyDSTool.Generator.Vode\_ODEsystem'.null\_predicate  
 PyDSTool.Generator.Vode\_ODEsystem'.pi  
 PyDSTool.Generator.Vode\_ODEsystem'.sign  
 PyDSTool.Generator.Vode\_ODEsystem'.symbolMapDict  
 PyDSTool.Generator.Vode\_ODEsystem'.targetLangs  
 PyDSTool.Generator.Vode\_ODEsystem'.theGenSpecHelper  
 PyDSTool.Generator.Vode\_ODEsystem'.uncertain  
 PyDSTool.Generator.Vode\_ODEsystem'.warnfields  
 PyDSTool.Generator.Vode\_ODEsystem'.warnmessages  
 PyDSTool.Generator.allimports.e  
 PyDSTool.Generator.allimports.pi  

PyDSTool.Generator.baseclasses.HAVE\_PSYCO

PyDSTool.Generator.baseclasses.\_1DimplicitSolveMethods

PyDSTool.Generator.baseclasses.\_all\_complex

PyDSTool.Generator.baseclasses.\_all\_float

PyDSTool.Generator.baseclasses.\_all\_int

PyDSTool.Generator.baseclasses.\_all\_numpy\_complex

PyDSTool.Generator.baseclasses.\_all\_numpy\_float

PyDSTool.Generator.baseclasses.\_all\_numpy\_int

PyDSTool.Generator.baseclasses.\_complex\_types

PyDSTool.Generator.baseclasses.\_float\_types

PyDSTool.Generator.baseclasses.\_implicitSolveMethods

PyDSTool.Generator.baseclasses.\_int\_types

PyDSTool.Generator.baseclasses.\_num\_equivtype

PyDSTool.Generator.baseclasses.\_num\_maxmin

PyDSTool.Generator.baseclasses.\_num\_name2equivtypes

PyDSTool.Generator.baseclasses.\_num\_name2type

PyDSTool.Generator.baseclasses.\_num\_type2name

PyDSTool.Generator.baseclasses.\_num\_types

PyDSTool.Generator.baseclasses.\_pytypefromtype

PyDSTool.Generator.baseclasses.\_real\_types

PyDSTool.Generator.baseclasses.\_seq\_types

PyDSTool.Generator.baseclasses.e

PyDSTool.Generator.baseclasses.genDB  

PyDSTool.Generator.baseclasses.pi

PyDSTool.Generator.baseclasses.theGenSpecHelper  
 PyDSTool.Generator.e  
 PyDSTool.Generator.messagecodes.E\_COMPUTFAIL  
 PyDSTool.Generator.messagecodes.E\_NONUNIQUETERM  
 PyDSTool.Generator.messagecodes.W\_BISECTLIMIT  
 PyDSTool.Generator.messagecodes.W\_NONTERMEVENT  
 PyDSTool.Generator.messagecodes.W\_NONTERMSTATEBD  
 PyDSTool.Generator.messagecodes.W\_TERMEVENT  
 PyDSTool.Generator.messagecodes.W\_TERMSTATEBD  
 PyDSTool.Generator.messagecodes.W\_UNCERTVAL  
 PyDSTool.Generator.messagecodes.errmessages  
 PyDSTool.Generator.messagecodes.errorfields  
 PyDSTool.Generator.messagecodes.warnfields  
 PyDSTool.Generator.messagecodes.warnmessages  
 PyDSTool.Generator.pi  
 PyDSTool.Getrandbits  
 PyDSTool.Getstate  
 PyDSTool.Hypot  
 PyDSTool.Infinity  

PyDSTool.Interval'.API

PyDSTool.Interval'.Continuous

PyDSTool.Interval'.Discrete

PyDSTool.Interval'.Inf

PyDSTool.Interval'.LargestInt32

PyDSTool.Interval'.MIN\_EXP

PyDSTool.Interval'.NaN

PyDSTool.Interval'.\_1DimplicitSolveMethods

PyDSTool.Interval'.\_all\_complex

PyDSTool.Interval'.\_all\_float

PyDSTool.Interval'.\_all\_int

PyDSTool.Interval'.\_all\_numpy\_complex

PyDSTool.Interval'.\_all\_numpy\_float

PyDSTool.Interval'.\_all\_numpy\_int

PyDSTool.Interval'.\_complex\_types

PyDSTool.Interval'.\_float\_types

PyDSTool.Interval'.\_implicitSolveMethods

PyDSTool.Interval'.\_int\_types

PyDSTool.Interval'.\_num\_equivtype

PyDSTool.Interval'.\_num\_maxmin

PyDSTool.Interval'.\_num\_name2equivtypes

PyDSTool.Interval'.\_num\_name2type

PyDSTool.Interval'.\_num\_type2name

PyDSTool.Interval'.\_num\_types

PyDSTool.Interval'.\_pytypefromtype

PyDSTool.Interval'.\_real\_types

PyDSTool.Interval'.\_seq\_types

PyDSTool.Interval'.c

PyDSTool.Interval'.contained  

PyDSTool.Interval'.isfinite

PyDSTool.Interval'.isinf

PyDSTool.Interval'.isnan

PyDSTool.Interval'.n

PyDSTool.Interval'.notcontained  

PyDSTool.Interval'.null\_predicate

PyDSTool.Interval'.re\_number

PyDSTool.Interval'.sign

PyDSTool.Interval'.targetLangs

PyDSTool.Interval'.u

PyDSTool.Interval'.uncertain  
 PyDSTool.Jumpahead  
 PyDSTool.Ldexp  
 PyDSTool.Log  
 PyDSTool.Log10  
 PyDSTool.Lognormvariate  
 PyDSTool.MAXDIMS  

PyDSTool.MProject.\_classes

PyDSTool.MProject.\_functions

PyDSTool.Max  
 PyDSTool.Min  

PyDSTool.Model.\_1DimplicitSolveMethods

PyDSTool.Model.\_all\_complex

PyDSTool.Model.\_all\_float

PyDSTool.Model.\_all\_int

PyDSTool.Model.\_all\_numpy\_complex

PyDSTool.Model.\_all\_numpy\_float

PyDSTool.Model.\_all\_numpy\_int

PyDSTool.Model.\_complex\_types

PyDSTool.Model.\_float\_types

PyDSTool.Model.\_implicitSolveMethods

PyDSTool.Model.\_int\_types

PyDSTool.Model.\_num\_equivtype

PyDSTool.Model.\_num\_maxmin

PyDSTool.Model.\_num\_name2equivtypes

PyDSTool.Model.\_num\_name2type

PyDSTool.Model.\_num\_type2name

PyDSTool.Model.\_num\_types

PyDSTool.Model.\_pytypefromtype

PyDSTool.Model.\_real\_types

PyDSTool.Model.\_seq\_types

PyDSTool.ModelConstructor'.API

PyDSTool.ModelConstructor'.Continuous

PyDSTool.ModelConstructor'.Discrete

PyDSTool.ModelConstructor'.Inf

PyDSTool.ModelConstructor'.LargestInt32

PyDSTool.ModelConstructor'.NAMESEP

PyDSTool.ModelConstructor'.NaN

PyDSTool.ModelConstructor'.\_all\_complex

PyDSTool.ModelConstructor'.\_all\_float

PyDSTool.ModelConstructor'.\_all\_int

PyDSTool.ModelConstructor'.\_all\_numpy\_complex

PyDSTool.ModelConstructor'.\_all\_numpy\_float

PyDSTool.ModelConstructor'.\_all\_numpy\_int

PyDSTool.ModelConstructor'.\_complex\_types

PyDSTool.ModelConstructor'.\_float\_types

PyDSTool.ModelConstructor'.\_int\_types

PyDSTool.ModelConstructor'.\_num\_equivtype

PyDSTool.ModelConstructor'.\_num\_maxmin

PyDSTool.ModelConstructor'.\_num\_name2equivtypes

PyDSTool.ModelConstructor'.\_num\_name2type

PyDSTool.ModelConstructor'.\_num\_type2name

PyDSTool.ModelConstructor'.\_num\_types

PyDSTool.ModelConstructor'.\_pytypefromtype

PyDSTool.ModelConstructor'.\_real\_types

PyDSTool.ModelConstructor'.\_seq\_types

PyDSTool.ModelConstructor'.isfinite

PyDSTool.ModelConstructor'.mathNameMap

PyDSTool.ModelConstructor'.null\_predicate

PyDSTool.ModelConstructor'.targetLangs

PyDSTool.ModelSpec'.API

PyDSTool.ModelSpec'.Abs

PyDSTool.ModelSpec'.Acos

PyDSTool.ModelSpec'.Asin

PyDSTool.ModelSpec'.Atan

PyDSTool.ModelSpec'.Atan2

PyDSTool.ModelSpec'.Betavariate

PyDSTool.ModelSpec'.Ceil

PyDSTool.ModelSpec'.Choice

PyDSTool.ModelSpec'.Continuous

PyDSTool.ModelSpec'.Cos

PyDSTool.ModelSpec'.Cosh

PyDSTool.ModelSpec'.Degrees

PyDSTool.ModelSpec'.Discrete

PyDSTool.ModelSpec'.E

PyDSTool.ModelSpec'.Exp

PyDSTool.ModelSpec'.Expovariate

PyDSTool.ModelSpec'.Fabs

PyDSTool.ModelSpec'.Floor

PyDSTool.ModelSpec'.Fmod

PyDSTool.ModelSpec'.Frexp

PyDSTool.ModelSpec'.Gammavariate

PyDSTool.ModelSpec'.Gauss

PyDSTool.ModelSpec'.Getrandbits

PyDSTool.ModelSpec'.Getstate

PyDSTool.ModelSpec'.Hypot

PyDSTool.ModelSpec'.Inf

PyDSTool.ModelSpec'.Jumpahead

PyDSTool.ModelSpec'.LargestInt32

PyDSTool.ModelSpec'.Ldexp

PyDSTool.ModelSpec'.Log

PyDSTool.ModelSpec'.Log10

PyDSTool.ModelSpec'.Lognormvariate

PyDSTool.ModelSpec'.Max

PyDSTool.ModelSpec'.Min

PyDSTool.ModelSpec'.Modf

PyDSTool.ModelSpec'.NAMESEP

PyDSTool.ModelSpec'.NaN

PyDSTool.ModelSpec'.Normalvariate

PyDSTool.ModelSpec'.ONES

PyDSTool.ModelSpec'.Paretovariate

PyDSTool.ModelSpec'.Pi

PyDSTool.ModelSpec'.Pow

PyDSTool.ModelSpec'.Radians

PyDSTool.ModelSpec'.Randint

PyDSTool.ModelSpec'.Random

PyDSTool.ModelSpec'.Randrange

PyDSTool.ModelSpec'.Sample

PyDSTool.ModelSpec'.Seed

PyDSTool.ModelSpec'.Setstate

PyDSTool.ModelSpec'.Shuffle

PyDSTool.ModelSpec'.Sin

PyDSTool.ModelSpec'.Sinh

PyDSTool.ModelSpec'.Sqrt

PyDSTool.ModelSpec'.Sum

PyDSTool.ModelSpec'.Systemrandom

PyDSTool.ModelSpec'.Tan

PyDSTool.ModelSpec'.Tanh

PyDSTool.ModelSpec'.Uniform

PyDSTool.ModelSpec'.Vonmisesvariate

PyDSTool.ModelSpec'.Weibullvariate

PyDSTool.ModelSpec'.Wichmannhill

PyDSTool.ModelSpec'.ZEROS

PyDSTool.ModelSpec'.\_1DimplicitSolveMethods

PyDSTool.ModelSpec'.\_all\_complex

PyDSTool.ModelSpec'.\_all\_float

PyDSTool.ModelSpec'.\_all\_int

PyDSTool.ModelSpec'.\_all\_numpy\_complex

PyDSTool.ModelSpec'.\_all\_numpy\_float

PyDSTool.ModelSpec'.\_all\_numpy\_int

PyDSTool.ModelSpec'.\_classes

PyDSTool.ModelSpec'.\_complex\_types

PyDSTool.ModelSpec'.\_float\_types

PyDSTool.ModelSpec'.\_functions

PyDSTool.ModelSpec'.\_implicitSolveMethods

PyDSTool.ModelSpec'.\_indentstr

PyDSTool.ModelSpec'.\_int\_types

PyDSTool.ModelSpec'.\_num\_equivtype

PyDSTool.ModelSpec'.\_num\_maxmin

PyDSTool.ModelSpec'.\_num\_name2equivtypes

PyDSTool.ModelSpec'.\_num\_name2type

PyDSTool.ModelSpec'.\_num\_type2name

PyDSTool.ModelSpec'.\_num\_types

PyDSTool.ModelSpec'.\_objects

PyDSTool.ModelSpec'.\_pytypefromtype

PyDSTool.ModelSpec'.\_real\_types

PyDSTool.ModelSpec'.\_seq\_types

PyDSTool.ModelSpec'.allmathnames

PyDSTool.ModelSpec'.allmathnames\_symbolic

PyDSTool.ModelSpec'.builtinFnSigInfo

PyDSTool.ModelSpec'.builtin\_auxnames

PyDSTool.ModelSpec'.e

PyDSTool.ModelSpec'.inverseMathNameMap

PyDSTool.ModelSpec'.isfinite

PyDSTool.ModelSpec'.mathNameMap

PyDSTool.ModelSpec'.mod

PyDSTool.ModelSpec'.nameResolver  

PyDSTool.ModelSpec'.name\_chars\_RE

PyDSTool.ModelSpec'.null\_predicate

PyDSTool.ModelSpec'.num\_chars

PyDSTool.ModelSpec'.pi

PyDSTool.ModelSpec'.protected\_allnames

PyDSTool.ModelSpec'.protected\_auxnamesDB

PyDSTool.ModelSpec'.protected\_macronames

PyDSTool.ModelSpec'.protected\_mathnames

PyDSTool.ModelSpec'.protected\_randomnames

PyDSTool.ModelSpec'.protected\_scipynames

PyDSTool.ModelSpec'.protected\_specialfns

PyDSTool.ModelSpec'.specTypes

PyDSTool.ModelSpec'.syms

PyDSTool.ModelSpec'.targetLangs

PyDSTool.Modf  
 PyDSTool.NAN  
 PyDSTool.NINF  
 PyDSTool.NZERO  
 PyDSTool.Normalvariate  
 PyDSTool.PINF  
 PyDSTool.PZERO  
 PyDSTool.Paretovariate  
 PyDSTool.Pi  

PyDSTool.Points.\_1DimplicitSolveMethods

PyDSTool.Points.\_all\_complex

PyDSTool.Points.\_all\_float

PyDSTool.Points.\_all\_int

PyDSTool.Points.\_all\_numpy\_complex

PyDSTool.Points.\_all\_numpy\_float

PyDSTool.Points.\_all\_numpy\_int

PyDSTool.Points.\_complex\_types

PyDSTool.Points.\_float\_types

PyDSTool.Points.\_implicitSolveMethods

PyDSTool.Points.\_int\_types

PyDSTool.Points.\_num\_equivtype

PyDSTool.Points.\_num\_maxmin

PyDSTool.Points.\_num\_name2equivtypes

PyDSTool.Points.\_num\_name2type

PyDSTool.Points.\_num\_type2name

PyDSTool.Points.\_num\_types

PyDSTool.Points.\_pytypefromtype

PyDSTool.Points.\_real\_types

PyDSTool.Points.\_seq\_types

PyDSTool.Points.point\_keys

PyDSTool.Pow  

PyDSTool.PyCont.BifPoint.\_classes

PyDSTool.PyCont.ContClass'.ALLOW\_THREADS

PyDSTool.PyCont.ContClass'.BUFSIZE

PyDSTool.PyCont.ContClass'.CLIP

PyDSTool.PyCont.ContClass'.DAILY

PyDSTool.PyCont.ContClass'.ERR\_CALL

PyDSTool.PyCont.ContClass'.ERR\_DEFAULT

PyDSTool.PyCont.ContClass'.ERR\_DEFAULT2

PyDSTool.PyCont.ContClass'.ERR\_IGNORE

PyDSTool.PyCont.ContClass'.ERR\_LOG

PyDSTool.PyCont.ContClass'.ERR\_PRINT

PyDSTool.PyCont.ContClass'.ERR\_RAISE

PyDSTool.PyCont.ContClass'.ERR\_WARN

PyDSTool.PyCont.ContClass'.FLOATING\_POINT\_SUPPORT

PyDSTool.PyCont.ContClass'.FPE\_DIVIDEBYZERO

PyDSTool.PyCont.ContClass'.FPE\_INVALID

PyDSTool.PyCont.ContClass'.FPE\_OVERFLOW

PyDSTool.PyCont.ContClass'.FPE\_UNDERFLOW

PyDSTool.PyCont.ContClass'.FR

PyDSTool.PyCont.ContClass'.False\_

PyDSTool.PyCont.ContClass'.HOURLY

PyDSTool.PyCont.ContClass'.Inf

PyDSTool.PyCont.ContClass'.Infinity

PyDSTool.PyCont.ContClass'.MAXDIMS

PyDSTool.PyCont.ContClass'.MINUTELY

PyDSTool.PyCont.ContClass'.MO

PyDSTool.PyCont.ContClass'.MONTHLY

PyDSTool.PyCont.ContClass'.NAN

PyDSTool.PyCont.ContClass'.NINF

PyDSTool.PyCont.ContClass'.NZERO

PyDSTool.PyCont.ContClass'.NaN

PyDSTool.PyCont.ContClass'.PINF

PyDSTool.PyCont.ContClass'.PZERO

PyDSTool.PyCont.ContClass'.RAISE

PyDSTool.PyCont.ContClass'.SA

PyDSTool.PyCont.ContClass'.SECONDLY

PyDSTool.PyCont.ContClass'.SHIFT\_DIVIDEBYZERO

PyDSTool.PyCont.ContClass'.SHIFT\_INVALID

PyDSTool.PyCont.ContClass'.SHIFT\_OVERFLOW

PyDSTool.PyCont.ContClass'.SHIFT\_UNDERFLOW

PyDSTool.PyCont.ContClass'.SU

PyDSTool.PyCont.ContClass'.ScalarType

PyDSTool.PyCont.ContClass'.TH

PyDSTool.PyCont.ContClass'.TU

PyDSTool.PyCont.ContClass'.True\_

PyDSTool.PyCont.ContClass'.UFUNC\_BUFSIZE\_DEFAULT

PyDSTool.PyCont.ContClass'.UFUNC\_PYVALS\_NAME

PyDSTool.PyCont.ContClass'.WE

PyDSTool.PyCont.ContClass'.WEEKLY

PyDSTool.PyCont.ContClass'.WRAP

PyDSTool.PyCont.ContClass'.YEARLY

PyDSTool.PyCont.ContClass'.\_classes

PyDSTool.PyCont.ContClass'.\_constants

PyDSTool.PyCont.ContClass'.\_pydstool\_path

PyDSTool.PyCont.ContClass'.absolute

PyDSTool.PyCont.ContClass'.add

PyDSTool.PyCont.ContClass'.arccos

PyDSTool.PyCont.ContClass'.arccosh

PyDSTool.PyCont.ContClass'.arcsin

PyDSTool.PyCont.ContClass'.arcsinh

PyDSTool.PyCont.ContClass'.arctan

PyDSTool.PyCont.ContClass'.arctan2

PyDSTool.PyCont.ContClass'.arctanh

PyDSTool.PyCont.ContClass'.auto\_list  

PyDSTool.PyCont.ContClass'.bitwise\_and

PyDSTool.PyCont.ContClass'.bitwise\_not

PyDSTool.PyCont.ContClass'.bitwise\_or

PyDSTool.PyCont.ContClass'.bitwise\_xor

PyDSTool.PyCont.ContClass'.c\_

PyDSTool.PyCont.ContClass'.cast

PyDSTool.PyCont.ContClass'.ceil

PyDSTool.PyCont.ContClass'.colorbar\_doc

PyDSTool.PyCont.ContClass'.conj

PyDSTool.PyCont.ContClass'.conjugate

PyDSTool.PyCont.ContClass'.copysign

PyDSTool.PyCont.ContClass'.cos

PyDSTool.PyCont.ContClass'.cosh

PyDSTool.PyCont.ContClass'.curve\_args\_list  
 PyDSTool.PyCont.ContClass'.curve\_list  

PyDSTool.PyCont.ContClass'.deg2rad

PyDSTool.PyCont.ContClass'.degrees

PyDSTool.PyCont.ContClass'.divide

PyDSTool.PyCont.ContClass'.e

PyDSTool.PyCont.ContClass'.equal

PyDSTool.PyCont.ContClass'.exp

PyDSTool.PyCont.ContClass'.exp2

PyDSTool.PyCont.ContClass'.expm1

PyDSTool.PyCont.ContClass'.fabs

PyDSTool.PyCont.ContClass'.floor

PyDSTool.PyCont.ContClass'.floor\_divide

PyDSTool.PyCont.ContClass'.fmax

PyDSTool.PyCont.ContClass'.fmin

PyDSTool.PyCont.ContClass'.fmod

PyDSTool.PyCont.ContClass'.frexp

PyDSTool.PyCont.ContClass'.greater

PyDSTool.PyCont.ContClass'.greater\_equal

PyDSTool.PyCont.ContClass'.hypot

PyDSTool.PyCont.ContClass'.index\_exp

PyDSTool.PyCont.ContClass'.inf

PyDSTool.PyCont.ContClass'.infty

PyDSTool.PyCont.ContClass'.invert

PyDSTool.PyCont.ContClass'.isfinite

PyDSTool.PyCont.ContClass'.isinf

PyDSTool.PyCont.ContClass'.isnan

PyDSTool.PyCont.ContClass'.ldexp

PyDSTool.PyCont.ContClass'.left\_shift

PyDSTool.PyCont.ContClass'.less

PyDSTool.PyCont.ContClass'.less\_equal

PyDSTool.PyCont.ContClass'.little\_endian

PyDSTool.PyCont.ContClass'.log

PyDSTool.PyCont.ContClass'.log10

PyDSTool.PyCont.ContClass'.log1p

PyDSTool.PyCont.ContClass'.logaddexp

PyDSTool.PyCont.ContClass'.logaddexp2

PyDSTool.PyCont.ContClass'.logical\_and

PyDSTool.PyCont.ContClass'.logical\_not

PyDSTool.PyCont.ContClass'.logical\_or

PyDSTool.PyCont.ContClass'.logical\_xor

PyDSTool.PyCont.ContClass'.maximum

PyDSTool.PyCont.ContClass'.mgrid

PyDSTool.PyCont.ContClass'.minimum

PyDSTool.PyCont.ContClass'.mod

PyDSTool.PyCont.ContClass'.modf

PyDSTool.PyCont.ContClass'.multiply

PyDSTool.PyCont.ContClass'.nan

PyDSTool.PyCont.ContClass'.nbytes

PyDSTool.PyCont.ContClass'.negative

PyDSTool.PyCont.ContClass'.newaxis

PyDSTool.PyCont.ContClass'.nextafter

PyDSTool.PyCont.ContClass'.not\_equal

PyDSTool.PyCont.ContClass'.ogrid

PyDSTool.PyCont.ContClass'.ones\_like

PyDSTool.PyCont.ContClass'.pi

PyDSTool.PyCont.ContClass'.r\_

PyDSTool.PyCont.ContClass'.rad2deg

PyDSTool.PyCont.ContClass'.radians

PyDSTool.PyCont.ContClass'.rcParams

PyDSTool.PyCont.ContClass'.rcParamsDefault

PyDSTool.PyCont.ContClass'.reciprocal

PyDSTool.PyCont.ContClass'.remainder

PyDSTool.PyCont.ContClass'.rerr

PyDSTool.PyCont.ContClass'.right\_shift

PyDSTool.PyCont.ContClass'.rint

PyDSTool.PyCont.ContClass'.rout

PyDSTool.PyCont.ContClass'.s\_

PyDSTool.PyCont.ContClass'.sctypeDict

PyDSTool.PyCont.ContClass'.sctypeNA

PyDSTool.PyCont.ContClass'.sctypes

PyDSTool.PyCont.ContClass'.sign

PyDSTool.PyCont.ContClass'.signbit

PyDSTool.PyCont.ContClass'.sin

PyDSTool.PyCont.ContClass'.sinh

PyDSTool.PyCont.ContClass'.spacing

PyDSTool.PyCont.ContClass'.sqrt

PyDSTool.PyCont.ContClass'.square

PyDSTool.PyCont.ContClass'.subtract

PyDSTool.PyCont.ContClass'.tan

PyDSTool.PyCont.ContClass'.tanh

PyDSTool.PyCont.ContClass'.true\_divide

PyDSTool.PyCont.ContClass'.trunc

PyDSTool.PyCont.ContClass'.typeDict

PyDSTool.PyCont.ContClass'.typeNA

PyDSTool.PyCont.ContClass'.typecodes

PyDSTool.PyCont.Continuation.FLOQ\_TOL  

PyDSTool.PyCont.Continuation.Inf

PyDSTool.PyCont.Continuation.NaN

PyDSTool.PyCont.Continuation.PI  

PyDSTool.PyCont.Continuation.\_classes

PyDSTool.PyCont.Continuation.\_constants

PyDSTool.PyCont.Continuation.all\_args\_list  
 PyDSTool.PyCont.Continuation.all\_curve\_types  
 PyDSTool.PyCont.Continuation.all\_point\_types  
 PyDSTool.PyCont.Continuation.argmax  
 PyDSTool.PyCont.Continuation.auto\_point\_types  
 PyDSTool.PyCont.Continuation.bif\_curve\_colors  
 PyDSTool.PyCont.Continuation.bif\_point\_colors  

PyDSTool.PyCont.Continuation.c\_

PyDSTool.PyCont.Continuation.clock  
 PyDSTool.PyCont.Continuation.conjugate  
 PyDSTool.PyCont.Continuation.cont\_args\_list  
 PyDSTool.PyCont.Continuation.cont\_bif\_points  

PyDSTool.PyCont.Continuation.divide

PyDSTool.PyCont.Continuation.equilibrium\_args\_list  
 PyDSTool.PyCont.Continuation.equilibrium\_bif\_points  
 PyDSTool.PyCont.Continuation.evec1\_standard  
 PyDSTool.PyCont.Continuation.fixedpoint\_args\_list  
 PyDSTool.PyCont.Continuation.fixedpoint\_bif\_points  
 PyDSTool.PyCont.Continuation.fold\_args\_list  
 PyDSTool.PyCont.Continuation.fold\_bif\_points  
 PyDSTool.PyCont.Continuation.hopf\_args\_list  
 PyDSTool.PyCont.Continuation.hopf\_bif\_points  
 PyDSTool.PyCont.Continuation.identity  

PyDSTool.PyCont.Continuation.isfinite

PyDSTool.PyCont.Continuation.isnan

PyDSTool.PyCont.Continuation.limitcycle\_args\_list  
 PyDSTool.PyCont.Continuation.limitcycle\_bif\_points  

PyDSTool.PyCont.Continuation.log10

PyDSTool.PyCont.Continuation.mod

PyDSTool.PyCont.Continuation.multiply  
 PyDSTool.PyCont.Continuation.ndarray  
 PyDSTool.PyCont.Continuation.negate  
 PyDSTool.PyCont.Continuation.other\_special\_points  
 PyDSTool.PyCont.Continuation.pointsToPointset  

PyDSTool.PyCont.Continuation.r\_

PyDSTool.PyCont.Continuation.sign

PyDSTool.PyCont.Continuation.solution\_measures  
 PyDSTool.PyCont.Continuation.solution\_measures\_list  
 PyDSTool.PyCont.Continuation.sqrt  
 PyDSTool.PyCont.Continuation.stab\_line\_styles  

PyDSTool.PyCont.Continuation.subtract

PyDSTool.PyCont.Continuation.sum  
 PyDSTool.PyCont.Continuation.userdefined\_args\_list  

PyDSTool.PyCont.Plotting.\_classes

PyDSTool.PyCont.Plotting.\_functions

PyDSTool.PyCont.Plotting.all\_curve\_types

PyDSTool.PyCont.Plotting.all\_point\_types

PyDSTool.PyCont.TestFunc.\_classes

PyDSTool.PyCont.misc.FLOQ\_TOL

PyDSTool.PyCont.misc.Inf

PyDSTool.PyCont.misc.NaN

PyDSTool.PyCont.misc.PI

PyDSTool.PyCont.misc.\_classes

PyDSTool.PyCont.misc.\_functions

PyDSTool.PyCont.misc.all\_curve\_types

PyDSTool.PyCont.misc.all\_point\_types

PyDSTool.PyCont.misc.c\_

PyDSTool.PyCont.misc.conjugate

PyDSTool.PyCont.misc.divide

PyDSTool.PyCont.misc.isfinite

PyDSTool.PyCont.misc.mod

PyDSTool.PyCont.misc.multiply

PyDSTool.PyCont.misc.r\_

PyDSTool.PyCont.misc.sign

PyDSTool.PyCont.misc.sqrt

PyDSTool.PyCont.misc.subtract

PyDSTool.RAISE  
 PyDSTool.Radians  
 PyDSTool.Randint  
 PyDSTool.Random  
 PyDSTool.Randrange  
 PyDSTool.Redirector.STDERR  
 PyDSTool.Redirector.STDOUT  
 PyDSTool.SHIFT\_DIVIDEBYZERO  
 PyDSTool.SHIFT\_INVALID  
 PyDSTool.SHIFT\_OVERFLOW  
 PyDSTool.SHIFT\_UNDERFLOW  
 PyDSTool.Sample  
 PyDSTool.ScalarType  
 PyDSTool.Seed  
 PyDSTool.Setstate  
 PyDSTool.Shuffle  
 PyDSTool.Sin  
 PyDSTool.Sinh  
 PyDSTool.Sqrt  
 PyDSTool.Sum  

PyDSTool.Symbolic.API

PyDSTool.Symbolic.API\_class  
 PyDSTool.Symbolic.Abs  
 PyDSTool.Symbolic.Acos  
 PyDSTool.Symbolic.Asin  
 PyDSTool.Symbolic.Atan  
 PyDSTool.Symbolic.Atan2  
 PyDSTool.Symbolic.Betavariate  
 PyDSTool.Symbolic.Ceil  
 PyDSTool.Symbolic.Choice  

PyDSTool.Symbolic.Continuous

PyDSTool.Symbolic.Cos  
 PyDSTool.Symbolic.Cosh  
 PyDSTool.Symbolic.DO\_DEC  
 PyDSTool.Symbolic.DO\_POW  
 PyDSTool.Symbolic.Degrees  

PyDSTool.Symbolic.Discrete

PyDSTool.Symbolic.DomainType  
 PyDSTool.Symbolic.E  
 PyDSTool.Symbolic.Exp  
 PyDSTool.Symbolic.Expovariate  
 PyDSTool.Symbolic.Fabs  
 PyDSTool.Symbolic.Floor  
 PyDSTool.Symbolic.Fmod  
 PyDSTool.Symbolic.Frexp  
 PyDSTool.Symbolic.Gammavariate  
 PyDSTool.Symbolic.Gauss  
 PyDSTool.Symbolic.Getrandbits  
 PyDSTool.Symbolic.Getstate  
 PyDSTool.Symbolic.Hypot  

PyDSTool.Symbolic.Inf

PyDSTool.Symbolic.Jumpahead  

PyDSTool.Symbolic.LargestInt32

PyDSTool.Symbolic.Ldexp  
 PyDSTool.Symbolic.Log  
 PyDSTool.Symbolic.Log10  
 PyDSTool.Symbolic.Lognormvariate  
 PyDSTool.Symbolic.Macheps  
 PyDSTool.Symbolic.Max  
 PyDSTool.Symbolic.Min  
 PyDSTool.Symbolic.Modf  

PyDSTool.Symbolic.NAMESEP

PyDSTool.Symbolic.NaN

PyDSTool.Symbolic.Normalvariate  

PyDSTool.Symbolic.ONES

PyDSTool.Symbolic.POW\_STR  
 PyDSTool.Symbolic.Paretovariate  
 PyDSTool.Symbolic.Pi  
 PyDSTool.Symbolic.Pow  
 PyDSTool.Symbolic.Radians  
 PyDSTool.Symbolic.Randint  
 PyDSTool.Symbolic.Random  
 PyDSTool.Symbolic.Randrange  
 PyDSTool.Symbolic.Sample  
 PyDSTool.Symbolic.Seed  
 PyDSTool.Symbolic.Setstate  
 PyDSTool.Symbolic.Shuffle  
 PyDSTool.Symbolic.Sin  
 PyDSTool.Symbolic.Sinh  
 PyDSTool.Symbolic.Sqrt  
 PyDSTool.Symbolic.Sum  
 PyDSTool.Symbolic.Systemrandom  
 PyDSTool.Symbolic.TENS  
 PyDSTool.Symbolic.Tan  
 PyDSTool.Symbolic.Tanh  
 PyDSTool.Symbolic.Uniform  
 PyDSTool.Symbolic.Vonmisesvariate  
 PyDSTool.Symbolic.Weibullvariate  
 PyDSTool.Symbolic.Wichmannhill  

PyDSTool.Symbolic.ZEROS

PyDSTool.Symbolic.\_1DimplicitSolveMethods

PyDSTool.Symbolic.\_all\_complex

PyDSTool.Symbolic.\_all\_float

PyDSTool.Symbolic.\_all\_int

PyDSTool.Symbolic.\_all\_numpy\_complex

PyDSTool.Symbolic.\_all\_numpy\_float

PyDSTool.Symbolic.\_all\_numpy\_int

PyDSTool.Symbolic.\_avoid\_math\_symbols

PyDSTool.Symbolic.\_classes

PyDSTool.Symbolic.\_complex\_types

PyDSTool.Symbolic.\_constants

PyDSTool.Symbolic.\_float\_types

PyDSTool.Symbolic.\_functions

PyDSTool.Symbolic.\_implicitSolveMethods

PyDSTool.Symbolic.\_indentstr

PyDSTool.Symbolic.\_int\_types

PyDSTool.Symbolic.\_localfuncnames

PyDSTool.Symbolic.\_num\_equivtype

PyDSTool.Symbolic.\_num\_maxmin

PyDSTool.Symbolic.\_num\_name2equivtypes

PyDSTool.Symbolic.\_num\_name2type

PyDSTool.Symbolic.\_num\_type2name

PyDSTool.Symbolic.\_num\_types

PyDSTool.Symbolic.\_pytypefromtype

PyDSTool.Symbolic.\_real\_types

PyDSTool.Symbolic.\_seq\_types

PyDSTool.Symbolic.a

PyDSTool.Symbolic.acos

PyDSTool.Symbolic.acosh

PyDSTool.Symbolic.all  
 PyDSTool.Symbolic.allmathnames  
 PyDSTool.Symbolic.allmathnames\_symbolic  
 PyDSTool.Symbolic.alphabet\_chars\_RE  
 PyDSTool.Symbolic.any  

PyDSTool.Symbolic.arccos

PyDSTool.Symbolic.arccosh

PyDSTool.Symbolic.arcsin

PyDSTool.Symbolic.arcsinh

PyDSTool.Symbolic.arctan

PyDSTool.Symbolic.arctan2

PyDSTool.Symbolic.arctanh

PyDSTool.Symbolic.argmax  
 PyDSTool.Symbolic.argmin  
 PyDSTool.Symbolic.argsort  

PyDSTool.Symbolic.argstr

PyDSTool.Symbolic.asarray  

PyDSTool.Symbolic.asin

PyDSTool.Symbolic.asinh

PyDSTool.Symbolic.atan

PyDSTool.Symbolic.atan2

PyDSTool.Symbolic.atanh

PyDSTool.Symbolic.atleast\_1d  

PyDSTool.Symbolic.builtinFnSigInfo

PyDSTool.Symbolic.builtin\_auxnames

PyDSTool.Symbolic.builtinlookup

PyDSTool.Symbolic.c

PyDSTool.Symbolic.ceil

PyDSTool.Symbolic.clip  
 PyDSTool.Symbolic.collect\_numbers  
 PyDSTool.Symbolic.complex  
 PyDSTool.Symbolic.complex128  
 PyDSTool.Symbolic.complex192  
 PyDSTool.Symbolic.complex64  
 PyDSTool.Symbolic.complex\_  
 PyDSTool.Symbolic.complexfloating  
 PyDSTool.Symbolic.concatenate  
 PyDSTool.Symbolic.convert\_power\_reserved\_keywords  

PyDSTool.Symbolic.cos

PyDSTool.Symbolic.cosh

PyDSTool.Symbolic.double  

PyDSTool.Symbolic.e

PyDSTool.Symbolic.ensureints  

PyDSTool.Symbolic.exp

PyDSTool.Symbolic.f

PyDSTool.Symbolic.fabs

PyDSTool.Symbolic.feval\_map\_const

PyDSTool.Symbolic.feval\_map\_symb

PyDSTool.Symbolic.finfo  
 PyDSTool.Symbolic.fit\_function  
 PyDSTool.Symbolic.float  
 PyDSTool.Symbolic.float32  
 PyDSTool.Symbolic.float96  
 PyDSTool.Symbolic.float\_  
 PyDSTool.Symbolic.floating  

PyDSTool.Symbolic.floor

PyDSTool.Symbolic.fmod

PyDSTool.Symbolic.fname

PyDSTool.Symbolic.frexp

PyDSTool.Symbolic.funcnames

PyDSTool.Symbolic.funcstr

PyDSTool.Symbolic.greater  

PyDSTool.Symbolic.hypot

PyDSTool.Symbolic.i

PyDSTool.Symbolic.int  
 PyDSTool.Symbolic.int0  
 PyDSTool.Symbolic.int16  
 PyDSTool.Symbolic.int64  
 PyDSTool.Symbolic.int8  
 PyDSTool.Symbolic.int\_  
 PyDSTool.Symbolic.integer  
 PyDSTool.Symbolic.inverseMathNameMap  
 PyDSTool.Symbolic.isVectorClause  

PyDSTool.Symbolic.isfinite

PyDSTool.Symbolic.ldexp

PyDSTool.Symbolic.less  
 PyDSTool.Symbolic.less\_equal  

PyDSTool.Symbolic.local\_fndef

PyDSTool.Symbolic.log

PyDSTool.Symbolic.log10

PyDSTool.Symbolic.logical\_or  

PyDSTool.Symbolic.m

PyDSTool.Symbolic.mat  
 PyDSTool.Symbolic.mathNameMap  

PyDSTool.Symbolic.math\_dir

PyDSTool.Symbolic.math\_globals

PyDSTool.Symbolic.mathlookup

PyDSTool.Symbolic.minpack  

PyDSTool.Symbolic.mod

PyDSTool.Symbolic.modf

PyDSTool.Symbolic.modlookup

PyDSTool.Symbolic.n

PyDSTool.Symbolic.name\_chars\_RE

PyDSTool.Symbolic.ndarray  
 PyDSTool.Symbolic.newton\_meth  
 PyDSTool.Symbolic.norm  
 PyDSTool.Symbolic.npy  

PyDSTool.Symbolic.null\_predicate

PyDSTool.Symbolic.null\_predicate\_class  

PyDSTool.Symbolic.num\_chars

PyDSTool.Symbolic.ones  
 PyDSTool.Symbolic.parser  

PyDSTool.Symbolic.pi

PyDSTool.Symbolic.pow

PyDSTool.Symbolic.power

PyDSTool.Symbolic.predicate\_op  

PyDSTool.Symbolic.protected\_allnames

PyDSTool.Symbolic.protected\_auxnamesDB

PyDSTool.Symbolic.protected\_macronames

PyDSTool.Symbolic.protected\_mathnames

PyDSTool.Symbolic.protected\_randomnames

PyDSTool.Symbolic.protected\_scipynames

PyDSTool.Symbolic.protected\_specialfns

PyDSTool.Symbolic.putmask  

PyDSTool.Symbolic.qtypes

PyDSTool.Symbolic.randomlookup

PyDSTool.Symbolic.re  
 PyDSTool.Symbolic.redirc  
 PyDSTool.Symbolic.replaceSepQSpec  
 PyDSTool.Symbolic.replaceSepStr  
 PyDSTool.Symbolic.rerr  
 PyDSTool.Symbolic.reserved\_keywords  
 PyDSTool.Symbolic.rout  
 PyDSTool.Symbolic.searchsorted  
 PyDSTool.Symbolic.shape  
 PyDSTool.Symbolic.sign  

PyDSTool.Symbolic.sin

PyDSTool.Symbolic.sinh

PyDSTool.Symbolic.specTypes  
 PyDSTool.Symbolic.specialfns  
 PyDSTool.Symbolic.spy  

PyDSTool.Symbolic.sqrt

PyDSTool.Symbolic.swapaxes  

PyDSTool.Symbolic.symb

PyDSTool.Symbolic.symbol  

PyDSTool.Symbolic.syms

PyDSTool.Symbolic.take  

PyDSTool.Symbolic.tan

PyDSTool.Symbolic.tanh

PyDSTool.Symbolic.targetLangs

PyDSTool.Symbolic.temp\_macro\_names  
 PyDSTool.Symbolic.temp\_macro\_names\_inv  
 PyDSTool.Symbolic.time  
 PyDSTool.Symbolic.toCircumflexSyntax  
 PyDSTool.Symbolic.toDoubleStarSyntax  
 PyDSTool.Symbolic.token  
 PyDSTool.Symbolic.transpose  
 PyDSTool.Symbolic.zeros  
 PyDSTool.Systemrandom  
 PyDSTool.Tan  
 PyDSTool.Tanh  
 PyDSTool.Toolbox.ActivationFuncs.ALLOW\_THREADS  
 PyDSTool.Toolbox.ActivationFuncs.Abs  
 PyDSTool.Toolbox.ActivationFuncs.Acos  
 PyDSTool.Toolbox.ActivationFuncs.Asin  
 PyDSTool.Toolbox.ActivationFuncs.Atan  
 PyDSTool.Toolbox.ActivationFuncs.Atan2  
 PyDSTool.Toolbox.ActivationFuncs.BUFSIZE  
 PyDSTool.Toolbox.ActivationFuncs.Betavariate  
 PyDSTool.Toolbox.ActivationFuncs.CLIP  
 PyDSTool.Toolbox.ActivationFuncs.Ceil  
 PyDSTool.Toolbox.ActivationFuncs.Choice  
 PyDSTool.Toolbox.ActivationFuncs.Cos  
 PyDSTool.Toolbox.ActivationFuncs.Cosh  
 PyDSTool.Toolbox.ActivationFuncs.Degrees  
 PyDSTool.Toolbox.ActivationFuncs.E  
 PyDSTool.Toolbox.ActivationFuncs.ERR\_CALL  
 PyDSTool.Toolbox.ActivationFuncs.ERR\_DEFAULT  
 PyDSTool.Toolbox.ActivationFuncs.ERR\_DEFAULT2  
 PyDSTool.Toolbox.ActivationFuncs.ERR\_IGNORE  
 PyDSTool.Toolbox.ActivationFuncs.ERR\_LOG  
 PyDSTool.Toolbox.ActivationFuncs.ERR\_PRINT  
 PyDSTool.Toolbox.ActivationFuncs.ERR\_RAISE  
 PyDSTool.Toolbox.ActivationFuncs.ERR\_WARN  
 PyDSTool.Toolbox.ActivationFuncs.Exp  
 PyDSTool.Toolbox.ActivationFuncs.Expovariate  
 PyDSTool.Toolbox.ActivationFuncs.FLOATING\_POINT\_SUPPORT  
 PyDSTool.Toolbox.ActivationFuncs.FPE\_DIVIDEBYZERO  
 PyDSTool.Toolbox.ActivationFuncs.FPE\_INVALID  
 PyDSTool.Toolbox.ActivationFuncs.FPE\_OVERFLOW  
 PyDSTool.Toolbox.ActivationFuncs.FPE\_UNDERFLOW  
 PyDSTool.Toolbox.ActivationFuncs.Fabs  
 PyDSTool.Toolbox.ActivationFuncs.False\_  
 PyDSTool.Toolbox.ActivationFuncs.Floor  
 PyDSTool.Toolbox.ActivationFuncs.Fmod  
 PyDSTool.Toolbox.ActivationFuncs.Frexp  
 PyDSTool.Toolbox.ActivationFuncs.Gammavariate  
 PyDSTool.Toolbox.ActivationFuncs.Gauss  
 PyDSTool.Toolbox.ActivationFuncs.Getrandbits  
 PyDSTool.Toolbox.ActivationFuncs.Getstate  
 PyDSTool.Toolbox.ActivationFuncs.Hypot  
 PyDSTool.Toolbox.ActivationFuncs.Infinity  
 PyDSTool.Toolbox.ActivationFuncs.Jumpahead  
 PyDSTool.Toolbox.ActivationFuncs.Ldexp  
 PyDSTool.Toolbox.ActivationFuncs.Log  
 PyDSTool.Toolbox.ActivationFuncs.Log10  
 PyDSTool.Toolbox.ActivationFuncs.Lognormvariate  
 PyDSTool.Toolbox.ActivationFuncs.MAXDIMS  
 PyDSTool.Toolbox.ActivationFuncs.Max  
 PyDSTool.Toolbox.ActivationFuncs.Min  
 PyDSTool.Toolbox.ActivationFuncs.Modf  
 PyDSTool.Toolbox.ActivationFuncs.NAN  
 PyDSTool.Toolbox.ActivationFuncs.NINF  
 PyDSTool.Toolbox.ActivationFuncs.NZERO  
 PyDSTool.Toolbox.ActivationFuncs.Normalvariate  
 PyDSTool.Toolbox.ActivationFuncs.PINF  
 PyDSTool.Toolbox.ActivationFuncs.PZERO  
 PyDSTool.Toolbox.ActivationFuncs.Paretovariate  
 PyDSTool.Toolbox.ActivationFuncs.Pi  
 PyDSTool.Toolbox.ActivationFuncs.Pow  
 PyDSTool.Toolbox.ActivationFuncs.RAISE  
 PyDSTool.Toolbox.ActivationFuncs.Radians  
 PyDSTool.Toolbox.ActivationFuncs.Randint  
 PyDSTool.Toolbox.ActivationFuncs.Random  
 PyDSTool.Toolbox.ActivationFuncs.Randrange  
 PyDSTool.Toolbox.ActivationFuncs.SHIFT\_DIVIDEBYZERO  
 PyDSTool.Toolbox.ActivationFuncs.SHIFT\_INVALID  
 PyDSTool.Toolbox.ActivationFuncs.SHIFT\_OVERFLOW  
 PyDSTool.Toolbox.ActivationFuncs.SHIFT\_UNDERFLOW  
 PyDSTool.Toolbox.ActivationFuncs.Sample  
 PyDSTool.Toolbox.ActivationFuncs.ScalarType  
 PyDSTool.Toolbox.ActivationFuncs.Seed  
 PyDSTool.Toolbox.ActivationFuncs.Setstate  
 PyDSTool.Toolbox.ActivationFuncs.Shuffle  
 PyDSTool.Toolbox.ActivationFuncs.Sin  
 PyDSTool.Toolbox.ActivationFuncs.Sinh  
 PyDSTool.Toolbox.ActivationFuncs.Sqrt  
 PyDSTool.Toolbox.ActivationFuncs.Sum  
 PyDSTool.Toolbox.ActivationFuncs.Systemrandom  
 PyDSTool.Toolbox.ActivationFuncs.Tan  
 PyDSTool.Toolbox.ActivationFuncs.Tanh  
 PyDSTool.Toolbox.ActivationFuncs.True\_  
 PyDSTool.Toolbox.ActivationFuncs.UFUNC\_BUFSIZE\_DEFAULT  
 PyDSTool.Toolbox.ActivationFuncs.UFUNC\_PYVALS\_NAME  
 PyDSTool.Toolbox.ActivationFuncs.Uniform  
 PyDSTool.Toolbox.ActivationFuncs.Vonmisesvariate  
 PyDSTool.Toolbox.ActivationFuncs.WRAP  
 PyDSTool.Toolbox.ActivationFuncs.Weibullvariate  
 PyDSTool.Toolbox.ActivationFuncs.Wichmannhill  
 PyDSTool.Toolbox.ActivationFuncs.absolute  
 PyDSTool.Toolbox.ActivationFuncs.add  
 PyDSTool.Toolbox.ActivationFuncs.bitwise\_and  
 PyDSTool.Toolbox.ActivationFuncs.bitwise\_not  
 PyDSTool.Toolbox.ActivationFuncs.bitwise\_or  
 PyDSTool.Toolbox.ActivationFuncs.bitwise\_xor  
 PyDSTool.Toolbox.ActivationFuncs.c\_  
 PyDSTool.Toolbox.ActivationFuncs.cast  
 PyDSTool.Toolbox.ActivationFuncs.conj  
 PyDSTool.Toolbox.ActivationFuncs.conjugate  
 PyDSTool.Toolbox.ActivationFuncs.copysign  
 PyDSTool.Toolbox.ActivationFuncs.deg2rad  
 PyDSTool.Toolbox.ActivationFuncs.divide  
 PyDSTool.Toolbox.ActivationFuncs.equal  
 PyDSTool.Toolbox.ActivationFuncs.exp2  
 PyDSTool.Toolbox.ActivationFuncs.expm1  
 PyDSTool.Toolbox.ActivationFuncs.floor\_divide  
 PyDSTool.Toolbox.ActivationFuncs.fmax  
 PyDSTool.Toolbox.ActivationFuncs.fmin  
 PyDSTool.Toolbox.ActivationFuncs.greater\_equal  
 PyDSTool.Toolbox.ActivationFuncs.index\_exp  
 PyDSTool.Toolbox.ActivationFuncs.inf  
 PyDSTool.Toolbox.ActivationFuncs.infty  
 PyDSTool.Toolbox.ActivationFuncs.invert  
 PyDSTool.Toolbox.ActivationFuncs.isinf  
 PyDSTool.Toolbox.ActivationFuncs.left\_shift  
 PyDSTool.Toolbox.ActivationFuncs.little\_endian  
 PyDSTool.Toolbox.ActivationFuncs.log1p  
 PyDSTool.Toolbox.ActivationFuncs.logaddexp  
 PyDSTool.Toolbox.ActivationFuncs.logaddexp2  
 PyDSTool.Toolbox.ActivationFuncs.logical\_and  
 PyDSTool.Toolbox.ActivationFuncs.logical\_not  
 PyDSTool.Toolbox.ActivationFuncs.logical\_xor  
 PyDSTool.Toolbox.ActivationFuncs.maximum  
 PyDSTool.Toolbox.ActivationFuncs.mgrid  
 PyDSTool.Toolbox.ActivationFuncs.minimum  
 PyDSTool.Toolbox.ActivationFuncs.multiply  
 PyDSTool.Toolbox.ActivationFuncs.n  
 PyDSTool.Toolbox.ActivationFuncs.nan  
 PyDSTool.Toolbox.ActivationFuncs.nbytes  
 PyDSTool.Toolbox.ActivationFuncs.negative  
 PyDSTool.Toolbox.ActivationFuncs.newaxis  
 PyDSTool.Toolbox.ActivationFuncs.nextafter  
 PyDSTool.Toolbox.ActivationFuncs.not\_equal  
 PyDSTool.Toolbox.ActivationFuncs.ogrid  
 PyDSTool.Toolbox.ActivationFuncs.ones\_like  
 PyDSTool.Toolbox.ActivationFuncs.r\_  
 PyDSTool.Toolbox.ActivationFuncs.rad2deg  
 PyDSTool.Toolbox.ActivationFuncs.reciprocal  
 PyDSTool.Toolbox.ActivationFuncs.remainder  
 PyDSTool.Toolbox.ActivationFuncs.right\_shift  
 PyDSTool.Toolbox.ActivationFuncs.rint  
 PyDSTool.Toolbox.ActivationFuncs.s\_  
 PyDSTool.Toolbox.ActivationFuncs.sctypeDict  
 PyDSTool.Toolbox.ActivationFuncs.sctypeNA  
 PyDSTool.Toolbox.ActivationFuncs.sctypes  
 PyDSTool.Toolbox.ActivationFuncs.signbit  
 PyDSTool.Toolbox.ActivationFuncs.spacing  
 PyDSTool.Toolbox.ActivationFuncs.square  
 PyDSTool.Toolbox.ActivationFuncs.subtract  
 PyDSTool.Toolbox.ActivationFuncs.t  
 PyDSTool.Toolbox.ActivationFuncs.true\_divide  
 PyDSTool.Toolbox.ActivationFuncs.trunc  
 PyDSTool.Toolbox.ActivationFuncs.typeDict  
 PyDSTool.Toolbox.ActivationFuncs.typeNA  
 PyDSTool.Toolbox.ActivationFuncs.typecodes  

PyDSTool.Toolbox.DSSRT\_tools.ALLOW\_THREADS

PyDSTool.Toolbox.DSSRT\_tools.Abs

PyDSTool.Toolbox.DSSRT\_tools.Acos

PyDSTool.Toolbox.DSSRT\_tools.Asin

PyDSTool.Toolbox.DSSRT\_tools.Atan

PyDSTool.Toolbox.DSSRT\_tools.Atan2

PyDSTool.Toolbox.DSSRT\_tools.BUFSIZE

PyDSTool.Toolbox.DSSRT\_tools.Betavariate

PyDSTool.Toolbox.DSSRT\_tools.CFGorder

PyDSTool.Toolbox.DSSRT\_tools.CLIP

PyDSTool.Toolbox.DSSRT\_tools.Ceil

PyDSTool.Toolbox.DSSRT\_tools.Choice

PyDSTool.Toolbox.DSSRT\_tools.Cos

PyDSTool.Toolbox.DSSRT\_tools.Cosh

PyDSTool.Toolbox.DSSRT\_tools.Degrees

PyDSTool.Toolbox.DSSRT\_tools.E

PyDSTool.Toolbox.DSSRT\_tools.ERR\_CALL

PyDSTool.Toolbox.DSSRT\_tools.ERR\_DEFAULT

PyDSTool.Toolbox.DSSRT\_tools.ERR\_DEFAULT2

PyDSTool.Toolbox.DSSRT\_tools.ERR\_IGNORE

PyDSTool.Toolbox.DSSRT\_tools.ERR\_LOG

PyDSTool.Toolbox.DSSRT\_tools.ERR\_PRINT

PyDSTool.Toolbox.DSSRT\_tools.ERR\_RAISE

PyDSTool.Toolbox.DSSRT\_tools.ERR\_WARN

PyDSTool.Toolbox.DSSRT\_tools.Exp

PyDSTool.Toolbox.DSSRT\_tools.Expovariate

PyDSTool.Toolbox.DSSRT\_tools.FLOATING\_POINT\_SUPPORT

PyDSTool.Toolbox.DSSRT\_tools.FPE\_DIVIDEBYZERO

PyDSTool.Toolbox.DSSRT\_tools.FPE\_INVALID

PyDSTool.Toolbox.DSSRT\_tools.FPE\_OVERFLOW

PyDSTool.Toolbox.DSSRT\_tools.FPE\_UNDERFLOW

PyDSTool.Toolbox.DSSRT\_tools.Fabs

PyDSTool.Toolbox.DSSRT\_tools.False\_

PyDSTool.Toolbox.DSSRT\_tools.Floor

PyDSTool.Toolbox.DSSRT\_tools.Fmod

PyDSTool.Toolbox.DSSRT\_tools.Frexp

PyDSTool.Toolbox.DSSRT\_tools.Gammavariate

PyDSTool.Toolbox.DSSRT\_tools.Gauss

PyDSTool.Toolbox.DSSRT\_tools.Getrandbits

PyDSTool.Toolbox.DSSRT\_tools.Getstate

PyDSTool.Toolbox.DSSRT\_tools.Hypot

PyDSTool.Toolbox.DSSRT\_tools.Infinity

PyDSTool.Toolbox.DSSRT\_tools.Jumpahead

PyDSTool.Toolbox.DSSRT\_tools.Ldexp

PyDSTool.Toolbox.DSSRT\_tools.Log

PyDSTool.Toolbox.DSSRT\_tools.Log10

PyDSTool.Toolbox.DSSRT\_tools.Lognormvariate

PyDSTool.Toolbox.DSSRT\_tools.MAXDIMS

PyDSTool.Toolbox.DSSRT\_tools.Max

PyDSTool.Toolbox.DSSRT\_tools.Min

PyDSTool.Toolbox.DSSRT\_tools.Modf

PyDSTool.Toolbox.DSSRT\_tools.NAN

PyDSTool.Toolbox.DSSRT\_tools.NINF

PyDSTool.Toolbox.DSSRT\_tools.NZERO

PyDSTool.Toolbox.DSSRT\_tools.Normalvariate

PyDSTool.Toolbox.DSSRT\_tools.PINF

PyDSTool.Toolbox.DSSRT\_tools.PZERO

PyDSTool.Toolbox.DSSRT\_tools.Paretovariate

PyDSTool.Toolbox.DSSRT\_tools.Pi

PyDSTool.Toolbox.DSSRT\_tools.Pow

PyDSTool.Toolbox.DSSRT\_tools.RAISE

PyDSTool.Toolbox.DSSRT\_tools.Radians

PyDSTool.Toolbox.DSSRT\_tools.Randint

PyDSTool.Toolbox.DSSRT\_tools.Random

PyDSTool.Toolbox.DSSRT\_tools.Randrange

PyDSTool.Toolbox.DSSRT\_tools.SHIFT\_DIVIDEBYZERO

PyDSTool.Toolbox.DSSRT\_tools.SHIFT\_INVALID

PyDSTool.Toolbox.DSSRT\_tools.SHIFT\_OVERFLOW

PyDSTool.Toolbox.DSSRT\_tools.SHIFT\_UNDERFLOW

PyDSTool.Toolbox.DSSRT\_tools.Sample

PyDSTool.Toolbox.DSSRT\_tools.ScalarType

PyDSTool.Toolbox.DSSRT\_tools.Seed

PyDSTool.Toolbox.DSSRT\_tools.Setstate

PyDSTool.Toolbox.DSSRT\_tools.Shuffle

PyDSTool.Toolbox.DSSRT\_tools.Sin

PyDSTool.Toolbox.DSSRT\_tools.Sinh

PyDSTool.Toolbox.DSSRT\_tools.Sqrt

PyDSTool.Toolbox.DSSRT\_tools.Sum

PyDSTool.Toolbox.DSSRT\_tools.Systemrandom

PyDSTool.Toolbox.DSSRT\_tools.Tan

PyDSTool.Toolbox.DSSRT\_tools.Tanh

PyDSTool.Toolbox.DSSRT\_tools.True\_

PyDSTool.Toolbox.DSSRT\_tools.UFUNC\_BUFSIZE\_DEFAULT

PyDSTool.Toolbox.DSSRT\_tools.UFUNC\_PYVALS\_NAME

PyDSTool.Toolbox.DSSRT\_tools.Uniform

PyDSTool.Toolbox.DSSRT\_tools.Vonmisesvariate

PyDSTool.Toolbox.DSSRT\_tools.WRAP

PyDSTool.Toolbox.DSSRT\_tools.Weibullvariate

PyDSTool.Toolbox.DSSRT\_tools.Wichmannhill

PyDSTool.Toolbox.DSSRT\_tools.absolute

PyDSTool.Toolbox.DSSRT\_tools.add

PyDSTool.Toolbox.DSSRT\_tools.bitwise\_and

PyDSTool.Toolbox.DSSRT\_tools.bitwise\_not

PyDSTool.Toolbox.DSSRT\_tools.bitwise\_or

PyDSTool.Toolbox.DSSRT\_tools.bitwise\_xor

PyDSTool.Toolbox.DSSRT\_tools.c\_

PyDSTool.Toolbox.DSSRT\_tools.cast

PyDSTool.Toolbox.DSSRT\_tools.conj

PyDSTool.Toolbox.DSSRT\_tools.conjugate

PyDSTool.Toolbox.DSSRT\_tools.copysign

PyDSTool.Toolbox.DSSRT\_tools.deg2rad

PyDSTool.Toolbox.DSSRT\_tools.divide

PyDSTool.Toolbox.DSSRT\_tools.equal

PyDSTool.Toolbox.DSSRT\_tools.exp2

PyDSTool.Toolbox.DSSRT\_tools.expm1

PyDSTool.Toolbox.DSSRT\_tools.floor\_divide

PyDSTool.Toolbox.DSSRT\_tools.fmax

PyDSTool.Toolbox.DSSRT\_tools.fmin

PyDSTool.Toolbox.DSSRT\_tools.greater\_equal

PyDSTool.Toolbox.DSSRT\_tools.index\_exp

PyDSTool.Toolbox.DSSRT\_tools.inf

PyDSTool.Toolbox.DSSRT\_tools.infty

PyDSTool.Toolbox.DSSRT\_tools.invert

PyDSTool.Toolbox.DSSRT\_tools.isinf

PyDSTool.Toolbox.DSSRT\_tools.left\_shift

PyDSTool.Toolbox.DSSRT\_tools.little\_endian

PyDSTool.Toolbox.DSSRT\_tools.log1p

PyDSTool.Toolbox.DSSRT\_tools.logaddexp

PyDSTool.Toolbox.DSSRT\_tools.logaddexp2

PyDSTool.Toolbox.DSSRT\_tools.logical\_and

PyDSTool.Toolbox.DSSRT\_tools.logical\_not

PyDSTool.Toolbox.DSSRT\_tools.logical\_xor

PyDSTool.Toolbox.DSSRT\_tools.maximum

PyDSTool.Toolbox.DSSRT\_tools.mgrid

PyDSTool.Toolbox.DSSRT\_tools.minimum

PyDSTool.Toolbox.DSSRT\_tools.multiply

PyDSTool.Toolbox.DSSRT\_tools.n

PyDSTool.Toolbox.DSSRT\_tools.nan

PyDSTool.Toolbox.DSSRT\_tools.nbytes

PyDSTool.Toolbox.DSSRT\_tools.negative

PyDSTool.Toolbox.DSSRT\_tools.newaxis

PyDSTool.Toolbox.DSSRT\_tools.nextafter

PyDSTool.Toolbox.DSSRT\_tools.nodes\_order

PyDSTool.Toolbox.DSSRT\_tools.not\_equal

PyDSTool.Toolbox.DSSRT\_tools.ogrid

PyDSTool.Toolbox.DSSRT\_tools.ones\_like

PyDSTool.Toolbox.DSSRT\_tools.r\_

PyDSTool.Toolbox.DSSRT\_tools.rad2deg

PyDSTool.Toolbox.DSSRT\_tools.reciprocal

PyDSTool.Toolbox.DSSRT\_tools.remainder

PyDSTool.Toolbox.DSSRT\_tools.right\_shift

PyDSTool.Toolbox.DSSRT\_tools.rint

PyDSTool.Toolbox.DSSRT\_tools.s\_

PyDSTool.Toolbox.DSSRT\_tools.sctypeDict

PyDSTool.Toolbox.DSSRT\_tools.sctypeNA

PyDSTool.Toolbox.DSSRT\_tools.sctypes

PyDSTool.Toolbox.DSSRT\_tools.signbit

PyDSTool.Toolbox.DSSRT\_tools.spacing

PyDSTool.Toolbox.DSSRT\_tools.square

PyDSTool.Toolbox.DSSRT\_tools.subtract

PyDSTool.Toolbox.DSSRT\_tools.t

PyDSTool.Toolbox.DSSRT\_tools.true\_divide

PyDSTool.Toolbox.DSSRT\_tools.trunc

PyDSTool.Toolbox.DSSRT\_tools.typeDict

PyDSTool.Toolbox.DSSRT\_tools.typeNA

PyDSTool.Toolbox.DSSRT\_tools.typecodes

PyDSTool.Toolbox.FR.tarray  
 PyDSTool.Toolbox.InputProfile.ALLOW\_THREADS  
 PyDSTool.Toolbox.InputProfile.Abs  
 PyDSTool.Toolbox.InputProfile.Acos  
 PyDSTool.Toolbox.InputProfile.Asin  
 PyDSTool.Toolbox.InputProfile.Atan  
 PyDSTool.Toolbox.InputProfile.Atan2  
 PyDSTool.Toolbox.InputProfile.BUFSIZE  
 PyDSTool.Toolbox.InputProfile.Betavariate  
 PyDSTool.Toolbox.InputProfile.CLIP  
 PyDSTool.Toolbox.InputProfile.Ceil  
 PyDSTool.Toolbox.InputProfile.Choice  
 PyDSTool.Toolbox.InputProfile.Cos  
 PyDSTool.Toolbox.InputProfile.Cosh  
 PyDSTool.Toolbox.InputProfile.Degrees  
 PyDSTool.Toolbox.InputProfile.E  
 PyDSTool.Toolbox.InputProfile.ERR\_CALL  
 PyDSTool.Toolbox.InputProfile.ERR\_DEFAULT  
 PyDSTool.Toolbox.InputProfile.ERR\_DEFAULT2  
 PyDSTool.Toolbox.InputProfile.ERR\_IGNORE  
 PyDSTool.Toolbox.InputProfile.ERR\_LOG  
 PyDSTool.Toolbox.InputProfile.ERR\_PRINT  
 PyDSTool.Toolbox.InputProfile.ERR\_RAISE  
 PyDSTool.Toolbox.InputProfile.ERR\_WARN  
 PyDSTool.Toolbox.InputProfile.Exp  
 PyDSTool.Toolbox.InputProfile.Expovariate  
 PyDSTool.Toolbox.InputProfile.FLOATING\_POINT\_SUPPORT  
 PyDSTool.Toolbox.InputProfile.FPE\_DIVIDEBYZERO  
 PyDSTool.Toolbox.InputProfile.FPE\_INVALID  
 PyDSTool.Toolbox.InputProfile.FPE\_OVERFLOW  
 PyDSTool.Toolbox.InputProfile.FPE\_UNDERFLOW  
 PyDSTool.Toolbox.InputProfile.Fabs  
 PyDSTool.Toolbox.InputProfile.False\_  
 PyDSTool.Toolbox.InputProfile.Floor  
 PyDSTool.Toolbox.InputProfile.Fmod  
 PyDSTool.Toolbox.InputProfile.Frexp  
 PyDSTool.Toolbox.InputProfile.Gammavariate  
 PyDSTool.Toolbox.InputProfile.Gauss  
 PyDSTool.Toolbox.InputProfile.Getrandbits  
 PyDSTool.Toolbox.InputProfile.Getstate  
 PyDSTool.Toolbox.InputProfile.Hypot  
 PyDSTool.Toolbox.InputProfile.Infinity  
 PyDSTool.Toolbox.InputProfile.Jumpahead  
 PyDSTool.Toolbox.InputProfile.Ldexp  
 PyDSTool.Toolbox.InputProfile.Log  
 PyDSTool.Toolbox.InputProfile.Log10  
 PyDSTool.Toolbox.InputProfile.Lognormvariate  
 PyDSTool.Toolbox.InputProfile.MAXDIMS  
 PyDSTool.Toolbox.InputProfile.Max  
 PyDSTool.Toolbox.InputProfile.Min  
 PyDSTool.Toolbox.InputProfile.Modf  
 PyDSTool.Toolbox.InputProfile.NAN  
 PyDSTool.Toolbox.InputProfile.NINF  
 PyDSTool.Toolbox.InputProfile.NZERO  
 PyDSTool.Toolbox.InputProfile.Normalvariate  
 PyDSTool.Toolbox.InputProfile.PINF  
 PyDSTool.Toolbox.InputProfile.PZERO  
 PyDSTool.Toolbox.InputProfile.Paretovariate  
 PyDSTool.Toolbox.InputProfile.Pi  
 PyDSTool.Toolbox.InputProfile.Pow  
 PyDSTool.Toolbox.InputProfile.RAISE  
 PyDSTool.Toolbox.InputProfile.Radians  
 PyDSTool.Toolbox.InputProfile.Randint  
 PyDSTool.Toolbox.InputProfile.Random  
 PyDSTool.Toolbox.InputProfile.Randrange  
 PyDSTool.Toolbox.InputProfile.SHIFT\_DIVIDEBYZERO  
 PyDSTool.Toolbox.InputProfile.SHIFT\_INVALID  
 PyDSTool.Toolbox.InputProfile.SHIFT\_OVERFLOW  
 PyDSTool.Toolbox.InputProfile.SHIFT\_UNDERFLOW  
 PyDSTool.Toolbox.InputProfile.Sample  
 PyDSTool.Toolbox.InputProfile.ScalarType  
 PyDSTool.Toolbox.InputProfile.Seed  
 PyDSTool.Toolbox.InputProfile.Setstate  
 PyDSTool.Toolbox.InputProfile.Shuffle  
 PyDSTool.Toolbox.InputProfile.Sin  
 PyDSTool.Toolbox.InputProfile.Sinh  
 PyDSTool.Toolbox.InputProfile.Sqrt  
 PyDSTool.Toolbox.InputProfile.Sum  
 PyDSTool.Toolbox.InputProfile.Systemrandom  
 PyDSTool.Toolbox.InputProfile.Tan  
 PyDSTool.Toolbox.InputProfile.Tanh  
 PyDSTool.Toolbox.InputProfile.True\_  
 PyDSTool.Toolbox.InputProfile.UFUNC\_BUFSIZE\_DEFAULT  
 PyDSTool.Toolbox.InputProfile.UFUNC\_PYVALS\_NAME  
 PyDSTool.Toolbox.InputProfile.Uniform  
 PyDSTool.Toolbox.InputProfile.Vonmisesvariate  
 PyDSTool.Toolbox.InputProfile.WRAP  
 PyDSTool.Toolbox.InputProfile.Weibullvariate  
 PyDSTool.Toolbox.InputProfile.Wichmannhill  
 PyDSTool.Toolbox.InputProfile.absolute  
 PyDSTool.Toolbox.InputProfile.add  
 PyDSTool.Toolbox.InputProfile.bitwise\_and  
 PyDSTool.Toolbox.InputProfile.bitwise\_not  
 PyDSTool.Toolbox.InputProfile.bitwise\_or  
 PyDSTool.Toolbox.InputProfile.bitwise\_xor  
 PyDSTool.Toolbox.InputProfile.c\_  
 PyDSTool.Toolbox.InputProfile.cast  
 PyDSTool.Toolbox.InputProfile.conj  
 PyDSTool.Toolbox.InputProfile.conjugate  
 PyDSTool.Toolbox.InputProfile.copysign  
 PyDSTool.Toolbox.InputProfile.deg2rad  
 PyDSTool.Toolbox.InputProfile.divide  
 PyDSTool.Toolbox.InputProfile.equal  
 PyDSTool.Toolbox.InputProfile.exp2  
 PyDSTool.Toolbox.InputProfile.expm1  
 PyDSTool.Toolbox.InputProfile.floor\_divide  
 PyDSTool.Toolbox.InputProfile.fmax  
 PyDSTool.Toolbox.InputProfile.fmin  
 PyDSTool.Toolbox.InputProfile.greater\_equal  
 PyDSTool.Toolbox.InputProfile.index\_exp  
 PyDSTool.Toolbox.InputProfile.inf  
 PyDSTool.Toolbox.InputProfile.infty  
 PyDSTool.Toolbox.InputProfile.invert  
 PyDSTool.Toolbox.InputProfile.isinf  
 PyDSTool.Toolbox.InputProfile.left\_shift  
 PyDSTool.Toolbox.InputProfile.little\_endian  
 PyDSTool.Toolbox.InputProfile.log1p  
 PyDSTool.Toolbox.InputProfile.logaddexp  
 PyDSTool.Toolbox.InputProfile.logaddexp2  
 PyDSTool.Toolbox.InputProfile.logical\_and  
 PyDSTool.Toolbox.InputProfile.logical\_not  
 PyDSTool.Toolbox.InputProfile.logical\_xor  
 PyDSTool.Toolbox.InputProfile.maximum  
 PyDSTool.Toolbox.InputProfile.mgrid  
 PyDSTool.Toolbox.InputProfile.minimum  
 PyDSTool.Toolbox.InputProfile.multiply  
 PyDSTool.Toolbox.InputProfile.n  
 PyDSTool.Toolbox.InputProfile.nan  
 PyDSTool.Toolbox.InputProfile.nbytes  
 PyDSTool.Toolbox.InputProfile.negative  
 PyDSTool.Toolbox.InputProfile.newaxis  
 PyDSTool.Toolbox.InputProfile.nextafter  
 PyDSTool.Toolbox.InputProfile.not\_equal  
 PyDSTool.Toolbox.InputProfile.ogrid  
 PyDSTool.Toolbox.InputProfile.ones\_like  
 PyDSTool.Toolbox.InputProfile.r\_  
 PyDSTool.Toolbox.InputProfile.rad2deg  
 PyDSTool.Toolbox.InputProfile.reciprocal  
 PyDSTool.Toolbox.InputProfile.remainder  
 PyDSTool.Toolbox.InputProfile.right\_shift  
 PyDSTool.Toolbox.InputProfile.rint  
 PyDSTool.Toolbox.InputProfile.s\_  
 PyDSTool.Toolbox.InputProfile.sctypeDict  
 PyDSTool.Toolbox.InputProfile.sctypeNA  
 PyDSTool.Toolbox.InputProfile.sctypes  
 PyDSTool.Toolbox.InputProfile.signbit  
 PyDSTool.Toolbox.InputProfile.spacing  
 PyDSTool.Toolbox.InputProfile.square  
 PyDSTool.Toolbox.InputProfile.subtract  
 PyDSTool.Toolbox.InputProfile.t  
 PyDSTool.Toolbox.InputProfile.true\_divide  
 PyDSTool.Toolbox.InputProfile.trunc  
 PyDSTool.Toolbox.InputProfile.typeDict  
 PyDSTool.Toolbox.InputProfile.typeNA  
 PyDSTool.Toolbox.InputProfile.typecodes  
 PyDSTool.Toolbox.ModelHelper.ALLOW\_THREADS  
 PyDSTool.Toolbox.ModelHelper.Abs  
 PyDSTool.Toolbox.ModelHelper.Acos  
 PyDSTool.Toolbox.ModelHelper.Asin  
 PyDSTool.Toolbox.ModelHelper.Atan  
 PyDSTool.Toolbox.ModelHelper.Atan2  
 PyDSTool.Toolbox.ModelHelper.BUFSIZE  
 PyDSTool.Toolbox.ModelHelper.Betavariate  
 PyDSTool.Toolbox.ModelHelper.CLIP  
 PyDSTool.Toolbox.ModelHelper.Ceil  
 PyDSTool.Toolbox.ModelHelper.Choice  
 PyDSTool.Toolbox.ModelHelper.Cos  
 PyDSTool.Toolbox.ModelHelper.Cosh  
 PyDSTool.Toolbox.ModelHelper.Degrees  
 PyDSTool.Toolbox.ModelHelper.E  
 PyDSTool.Toolbox.ModelHelper.ERR\_CALL  
 PyDSTool.Toolbox.ModelHelper.ERR\_DEFAULT  
 PyDSTool.Toolbox.ModelHelper.ERR\_DEFAULT2  
 PyDSTool.Toolbox.ModelHelper.ERR\_IGNORE  
 PyDSTool.Toolbox.ModelHelper.ERR\_LOG  
 PyDSTool.Toolbox.ModelHelper.ERR\_PRINT  
 PyDSTool.Toolbox.ModelHelper.ERR\_RAISE  
 PyDSTool.Toolbox.ModelHelper.ERR\_WARN  
 PyDSTool.Toolbox.ModelHelper.Exp  
 PyDSTool.Toolbox.ModelHelper.Expovariate  
 PyDSTool.Toolbox.ModelHelper.FLOATING\_POINT\_SUPPORT  
 PyDSTool.Toolbox.ModelHelper.FPE\_DIVIDEBYZERO  
 PyDSTool.Toolbox.ModelHelper.FPE\_INVALID  
 PyDSTool.Toolbox.ModelHelper.FPE\_OVERFLOW  
 PyDSTool.Toolbox.ModelHelper.FPE\_UNDERFLOW  
 PyDSTool.Toolbox.ModelHelper.Fabs  
 PyDSTool.Toolbox.ModelHelper.False\_  
 PyDSTool.Toolbox.ModelHelper.Floor  
 PyDSTool.Toolbox.ModelHelper.Fmod  
 PyDSTool.Toolbox.ModelHelper.Frexp  
 PyDSTool.Toolbox.ModelHelper.Gammavariate  
 PyDSTool.Toolbox.ModelHelper.Gauss  
 PyDSTool.Toolbox.ModelHelper.Getrandbits  
 PyDSTool.Toolbox.ModelHelper.Getstate  
 PyDSTool.Toolbox.ModelHelper.Hypot  
 PyDSTool.Toolbox.ModelHelper.Infinity  
 PyDSTool.Toolbox.ModelHelper.Jumpahead  
 PyDSTool.Toolbox.ModelHelper.Ldexp  
 PyDSTool.Toolbox.ModelHelper.Log  
 PyDSTool.Toolbox.ModelHelper.Log10  
 PyDSTool.Toolbox.ModelHelper.Lognormvariate  
 PyDSTool.Toolbox.ModelHelper.MAXDIMS  
 PyDSTool.Toolbox.ModelHelper.Max  
 PyDSTool.Toolbox.ModelHelper.Min  
 PyDSTool.Toolbox.ModelHelper.Modf  
 PyDSTool.Toolbox.ModelHelper.NAN  
 PyDSTool.Toolbox.ModelHelper.NINF  
 PyDSTool.Toolbox.ModelHelper.NZERO  
 PyDSTool.Toolbox.ModelHelper.Normalvariate  
 PyDSTool.Toolbox.ModelHelper.PINF  
 PyDSTool.Toolbox.ModelHelper.PZERO  
 PyDSTool.Toolbox.ModelHelper.Paretovariate  
 PyDSTool.Toolbox.ModelHelper.Pi  
 PyDSTool.Toolbox.ModelHelper.Pow  
 PyDSTool.Toolbox.ModelHelper.RAISE  
 PyDSTool.Toolbox.ModelHelper.Radians  
 PyDSTool.Toolbox.ModelHelper.Randint  
 PyDSTool.Toolbox.ModelHelper.Random  
 PyDSTool.Toolbox.ModelHelper.Randrange  
 PyDSTool.Toolbox.ModelHelper.SHIFT\_DIVIDEBYZERO  
 PyDSTool.Toolbox.ModelHelper.SHIFT\_INVALID  
 PyDSTool.Toolbox.ModelHelper.SHIFT\_OVERFLOW  
 PyDSTool.Toolbox.ModelHelper.SHIFT\_UNDERFLOW  
 PyDSTool.Toolbox.ModelHelper.Sample  
 PyDSTool.Toolbox.ModelHelper.ScalarType  
 PyDSTool.Toolbox.ModelHelper.Seed  
 PyDSTool.Toolbox.ModelHelper.Setstate  
 PyDSTool.Toolbox.ModelHelper.Shuffle  
 PyDSTool.Toolbox.ModelHelper.Sin  
 PyDSTool.Toolbox.ModelHelper.Sinh  
 PyDSTool.Toolbox.ModelHelper.Sqrt  
 PyDSTool.Toolbox.ModelHelper.Sum  
 PyDSTool.Toolbox.ModelHelper.Systemrandom  
 PyDSTool.Toolbox.ModelHelper.Tan  
 PyDSTool.Toolbox.ModelHelper.Tanh  
 PyDSTool.Toolbox.ModelHelper.True\_  
 PyDSTool.Toolbox.ModelHelper.UFUNC\_BUFSIZE\_DEFAULT  
 PyDSTool.Toolbox.ModelHelper.UFUNC\_PYVALS\_NAME  
 PyDSTool.Toolbox.ModelHelper.Uniform  
 PyDSTool.Toolbox.ModelHelper.Vonmisesvariate  
 PyDSTool.Toolbox.ModelHelper.WRAP  
 PyDSTool.Toolbox.ModelHelper.Weibullvariate  
 PyDSTool.Toolbox.ModelHelper.Wichmannhill  
 PyDSTool.Toolbox.ModelHelper.absolute  
 PyDSTool.Toolbox.ModelHelper.add  
 PyDSTool.Toolbox.ModelHelper.allODEgens  
 PyDSTool.Toolbox.ModelHelper.bitwise\_and  
 PyDSTool.Toolbox.ModelHelper.bitwise\_not  
 PyDSTool.Toolbox.ModelHelper.bitwise\_or  
 PyDSTool.Toolbox.ModelHelper.bitwise\_xor  
 PyDSTool.Toolbox.ModelHelper.c\_  
 PyDSTool.Toolbox.ModelHelper.cast  
 PyDSTool.Toolbox.ModelHelper.conj  
 PyDSTool.Toolbox.ModelHelper.conjugate  
 PyDSTool.Toolbox.ModelHelper.copysign  
 PyDSTool.Toolbox.ModelHelper.deg2rad  
 PyDSTool.Toolbox.ModelHelper.divide  
 PyDSTool.Toolbox.ModelHelper.equal  
 PyDSTool.Toolbox.ModelHelper.exp2  
 PyDSTool.Toolbox.ModelHelper.expm1  
 PyDSTool.Toolbox.ModelHelper.floor\_divide  
 PyDSTool.Toolbox.ModelHelper.fmax  
 PyDSTool.Toolbox.ModelHelper.fmin  
 PyDSTool.Toolbox.ModelHelper.greater\_equal  
 PyDSTool.Toolbox.ModelHelper.index\_exp  
 PyDSTool.Toolbox.ModelHelper.inf  
 PyDSTool.Toolbox.ModelHelper.infty  
 PyDSTool.Toolbox.ModelHelper.invert  
 PyDSTool.Toolbox.ModelHelper.isinf  
 PyDSTool.Toolbox.ModelHelper.left\_shift  
 PyDSTool.Toolbox.ModelHelper.little\_endian  
 PyDSTool.Toolbox.ModelHelper.log1p  
 PyDSTool.Toolbox.ModelHelper.logaddexp  
 PyDSTool.Toolbox.ModelHelper.logaddexp2  
 PyDSTool.Toolbox.ModelHelper.logical\_and  
 PyDSTool.Toolbox.ModelHelper.logical\_not  
 PyDSTool.Toolbox.ModelHelper.logical\_xor  
 PyDSTool.Toolbox.ModelHelper.maximum  
 PyDSTool.Toolbox.ModelHelper.mgrid  
 PyDSTool.Toolbox.ModelHelper.minimum  
 PyDSTool.Toolbox.ModelHelper.multiply  
 PyDSTool.Toolbox.ModelHelper.n  
 PyDSTool.Toolbox.ModelHelper.nan  
 PyDSTool.Toolbox.ModelHelper.nbytes  
 PyDSTool.Toolbox.ModelHelper.negative  
 PyDSTool.Toolbox.ModelHelper.newaxis  
 PyDSTool.Toolbox.ModelHelper.nextafter  
 PyDSTool.Toolbox.ModelHelper.not\_equal  
 PyDSTool.Toolbox.ModelHelper.ogrid  
 PyDSTool.Toolbox.ModelHelper.ones\_like  
 PyDSTool.Toolbox.ModelHelper.r\_  
 PyDSTool.Toolbox.ModelHelper.rad2deg  
 PyDSTool.Toolbox.ModelHelper.reciprocal  
 PyDSTool.Toolbox.ModelHelper.remainder  
 PyDSTool.Toolbox.ModelHelper.right\_shift  
 PyDSTool.Toolbox.ModelHelper.rint  
 PyDSTool.Toolbox.ModelHelper.s\_  
 PyDSTool.Toolbox.ModelHelper.sctypeDict  
 PyDSTool.Toolbox.ModelHelper.sctypeNA  
 PyDSTool.Toolbox.ModelHelper.sctypes  
 PyDSTool.Toolbox.ModelHelper.signbit  
 PyDSTool.Toolbox.ModelHelper.spacing  
 PyDSTool.Toolbox.ModelHelper.square  
 PyDSTool.Toolbox.ModelHelper.subtract  
 PyDSTool.Toolbox.ModelHelper.t  
 PyDSTool.Toolbox.ModelHelper.true\_divide  
 PyDSTool.Toolbox.ModelHelper.trunc  
 PyDSTool.Toolbox.ModelHelper.typeDict  
 PyDSTool.Toolbox.ModelHelper.typeNA  
 PyDSTool.Toolbox.ModelHelper.typecodes  

PyDSTool.Toolbox.NineML.ALLOW\_THREADS

PyDSTool.Toolbox.NineML.API

PyDSTool.Toolbox.NineML.API\_class  

PyDSTool.Toolbox.NineML.Abs

PyDSTool.Toolbox.NineML.Acos

PyDSTool.Toolbox.NineML.Asin

PyDSTool.Toolbox.NineML.Atan

PyDSTool.Toolbox.NineML.Atan2

PyDSTool.Toolbox.NineML.BUFSIZE

PyDSTool.Toolbox.NineML.Betavariate

PyDSTool.Toolbox.NineML.CLIP

PyDSTool.Toolbox.NineML.Ceil

PyDSTool.Toolbox.NineML.Choice

PyDSTool.Toolbox.NineML.Continuous

PyDSTool.Toolbox.NineML.Cos

PyDSTool.Toolbox.NineML.Cosh

PyDSTool.Toolbox.NineML.DO\_DEC  
 PyDSTool.Toolbox.NineML.DO\_POW  

PyDSTool.Toolbox.NineML.Degrees

PyDSTool.Toolbox.NineML.Discrete

PyDSTool.Toolbox.NineML.DomainType  

PyDSTool.Toolbox.NineML.E

PyDSTool.Toolbox.NineML.ERR\_CALL

PyDSTool.Toolbox.NineML.ERR\_DEFAULT

PyDSTool.Toolbox.NineML.ERR\_DEFAULT2

PyDSTool.Toolbox.NineML.ERR\_IGNORE

PyDSTool.Toolbox.NineML.ERR\_LOG

PyDSTool.Toolbox.NineML.ERR\_PRINT

PyDSTool.Toolbox.NineML.ERR\_RAISE

PyDSTool.Toolbox.NineML.ERR\_WARN

PyDSTool.Toolbox.NineML.E\_COMPUTFAIL

PyDSTool.Toolbox.NineML.E\_NONUNIQUETERM

PyDSTool.Toolbox.NineML.Exp

PyDSTool.Toolbox.NineML.Expovariate

PyDSTool.Toolbox.NineML.FLOATING\_POINT\_SUPPORT

PyDSTool.Toolbox.NineML.FPE\_DIVIDEBYZERO

PyDSTool.Toolbox.NineML.FPE\_INVALID

PyDSTool.Toolbox.NineML.FPE\_OVERFLOW

PyDSTool.Toolbox.NineML.FPE\_UNDERFLOW

PyDSTool.Toolbox.NineML.Fabs

PyDSTool.Toolbox.NineML.False\_

PyDSTool.Toolbox.NineML.Floor

PyDSTool.Toolbox.NineML.Fmod

PyDSTool.Toolbox.NineML.Frexp

PyDSTool.Toolbox.NineML.Gammavariate

PyDSTool.Toolbox.NineML.Gauss

PyDSTool.Toolbox.NineML.Getrandbits

PyDSTool.Toolbox.NineML.Getstate

PyDSTool.Toolbox.NineML.HAVE\_PSYCO

PyDSTool.Toolbox.NineML.Hypot

PyDSTool.Toolbox.NineML.Inf

PyDSTool.Toolbox.NineML.Infinity

PyDSTool.Toolbox.NineML.Jumpahead

PyDSTool.Toolbox.NineML.LargestInt32

PyDSTool.Toolbox.NineML.Ldexp

PyDSTool.Toolbox.NineML.Log

PyDSTool.Toolbox.NineML.Log10

PyDSTool.Toolbox.NineML.Lognormvariate

PyDSTool.Toolbox.NineML.MAXDIMS

PyDSTool.Toolbox.NineML.MReg  
 PyDSTool.Toolbox.NineML.Macheps  

PyDSTool.Toolbox.NineML.Max

PyDSTool.Toolbox.NineML.Min

PyDSTool.Toolbox.NineML.Modf

PyDSTool.Toolbox.NineML.NAMESEP  

PyDSTool.Toolbox.NineML.NAN

PyDSTool.Toolbox.NineML.NINF

PyDSTool.Toolbox.NineML.NZERO

PyDSTool.Toolbox.NineML.NaN

PyDSTool.Toolbox.NineML.Normalvariate

PyDSTool.Toolbox.NineML.ONES  

PyDSTool.Toolbox.NineML.PINF

PyDSTool.Toolbox.NineML.POW\_STR  

PyDSTool.Toolbox.NineML.PZERO

PyDSTool.Toolbox.NineML.Paretovariate

PyDSTool.Toolbox.NineML.Pi

PyDSTool.Toolbox.NineML.Pow

PyDSTool.Toolbox.NineML.RAISE

PyDSTool.Toolbox.NineML.Radians

PyDSTool.Toolbox.NineML.Randint

PyDSTool.Toolbox.NineML.Random

PyDSTool.Toolbox.NineML.Randrange

PyDSTool.Toolbox.NineML.SHIFT\_DIVIDEBYZERO

PyDSTool.Toolbox.NineML.SHIFT\_INVALID

PyDSTool.Toolbox.NineML.SHIFT\_OVERFLOW

PyDSTool.Toolbox.NineML.SHIFT\_UNDERFLOW

PyDSTool.Toolbox.NineML.Sample

PyDSTool.Toolbox.NineML.ScalarType

PyDSTool.Toolbox.NineML.Seed

PyDSTool.Toolbox.NineML.Setstate

PyDSTool.Toolbox.NineML.Shuffle

PyDSTool.Toolbox.NineML.Sin

PyDSTool.Toolbox.NineML.Sinh

PyDSTool.Toolbox.NineML.Sqrt

PyDSTool.Toolbox.NineML.Sum

PyDSTool.Toolbox.NineML.Systemrandom

PyDSTool.Toolbox.NineML.TENS  

PyDSTool.Toolbox.NineML.Tan

PyDSTool.Toolbox.NineML.Tanh

PyDSTool.Toolbox.NineML.True\_

PyDSTool.Toolbox.NineML.UFUNC\_BUFSIZE\_DEFAULT

PyDSTool.Toolbox.NineML.UFUNC\_PYVALS\_NAME

PyDSTool.Toolbox.NineML.Uniform

PyDSTool.Toolbox.NineML.Vonmisesvariate

PyDSTool.Toolbox.NineML.WRAP

PyDSTool.Toolbox.NineML.W\_BISECTLIMIT

PyDSTool.Toolbox.NineML.W\_NONTERMEVENT

PyDSTool.Toolbox.NineML.W\_NONTERMSTATEBD

PyDSTool.Toolbox.NineML.W\_TERMEVENT

PyDSTool.Toolbox.NineML.W\_TERMSTATEBD

PyDSTool.Toolbox.NineML.W\_UNCERTVAL

PyDSTool.Toolbox.NineML.Weibullvariate

PyDSTool.Toolbox.NineML.Wichmannhill

PyDSTool.Toolbox.NineML.ZEROS  

PyDSTool.Toolbox.NineML.\_classes

PyDSTool.Toolbox.NineML.\_features

PyDSTool.Toolbox.NineML.\_functions

PyDSTool.Toolbox.NineML.\_num\_types

PyDSTool.Toolbox.NineML.\_seq\_types

PyDSTool.Toolbox.NineML.absolute

PyDSTool.Toolbox.NineML.accrueCompTypes  
 PyDSTool.Toolbox.NineML.acosh  

PyDSTool.Toolbox.NineML.add

PyDSTool.Toolbox.NineML.allCompTypes  

PyDSTool.Toolbox.NineML.allODEgens

PyDSTool.Toolbox.NineML.allmathnames

PyDSTool.Toolbox.NineML.allmathnames\_symbolic

PyDSTool.Toolbox.NineML.alphabet\_chars\_RE  

PyDSTool.Toolbox.NineML.arccos

PyDSTool.Toolbox.NineML.arccosh

PyDSTool.Toolbox.NineML.arcsin

PyDSTool.Toolbox.NineML.arcsinh

PyDSTool.Toolbox.NineML.arctan

PyDSTool.Toolbox.NineML.arctan2

PyDSTool.Toolbox.NineML.arctanh

PyDSTool.Toolbox.NineML.asinh  
 PyDSTool.Toolbox.NineML.ast2shortlist  
 PyDSTool.Toolbox.NineML.ast2string  
 PyDSTool.Toolbox.NineML.atanh  

PyDSTool.Toolbox.NineML.bitwise\_and

PyDSTool.Toolbox.NineML.bitwise\_not

PyDSTool.Toolbox.NineML.bitwise\_or

PyDSTool.Toolbox.NineML.bitwise\_xor

PyDSTool.Toolbox.NineML.builtinFnSigInfo  
 PyDSTool.Toolbox.NineML.builtin\_auxnames  
 PyDSTool.Toolbox.NineML.builtinlookup  
 PyDSTool.Toolbox.NineML.cPickle  

PyDSTool.Toolbox.NineML.c\_

PyDSTool.Toolbox.NineML.cast

PyDSTool.Toolbox.NineML.ceil

PyDSTool.Toolbox.NineML.collect\_numbers  
 PyDSTool.Toolbox.NineML.complex  
 PyDSTool.Toolbox.NineML.complex192  

PyDSTool.Toolbox.NineML.conj

PyDSTool.Toolbox.NineML.conjugate

PyDSTool.Toolbox.NineML.contained

PyDSTool.Toolbox.NineML.convert\_power\_reserved\_keywords  

PyDSTool.Toolbox.NineML.copysign

PyDSTool.Toolbox.NineML.cos

PyDSTool.Toolbox.NineML.cosh

PyDSTool.Toolbox.NineML.count\_sep  

PyDSTool.Toolbox.NineML.deg2rad

PyDSTool.Toolbox.NineML.degrees

PyDSTool.Toolbox.NineML.divide

PyDSTool.Toolbox.NineML.doadd  
 PyDSTool.Toolbox.NineML.dodiv  
 PyDSTool.Toolbox.NineML.dofun  
 PyDSTool.Toolbox.NineML.domul  
 PyDSTool.Toolbox.NineML.doneg  
 PyDSTool.Toolbox.NineML.dopower  
 PyDSTool.Toolbox.NineML.dosub  
 PyDSTool.Toolbox.NineML.dsInterface  

PyDSTool.Toolbox.NineML.e

PyDSTool.Toolbox.NineML.ensurebare  
 PyDSTool.Toolbox.NineML.ensuredecimalconst  
 PyDSTool.Toolbox.NineML.ensureints  
 PyDSTool.Toolbox.NineML.ensureparen  
 PyDSTool.Toolbox.NineML.ensureparen\_div  

PyDSTool.Toolbox.NineML.equal

PyDSTool.Toolbox.NineML.errmessages

PyDSTool.Toolbox.NineML.errorfields

PyDSTool.Toolbox.NineML.exp

PyDSTool.Toolbox.NineML.exp2

PyDSTool.Toolbox.NineML.expm1

PyDSTool.Toolbox.NineML.fabs

PyDSTool.Toolbox.NineML.feature\_leaf  
 PyDSTool.Toolbox.NineML.feature\_node  
 PyDSTool.Toolbox.NineML.feval\_map\_const  
 PyDSTool.Toolbox.NineML.feval\_map\_symb  
 PyDSTool.Toolbox.NineML.findEndBrace  
 PyDSTool.Toolbox.NineML.findNumTailPos  
 PyDSTool.Toolbox.NineML.fit\_function  
 PyDSTool.Toolbox.NineML.float96  

PyDSTool.Toolbox.NineML.floor

PyDSTool.Toolbox.NineML.floor\_divide

PyDSTool.Toolbox.NineML.fmax

PyDSTool.Toolbox.NineML.fmin

PyDSTool.Toolbox.NineML.fmod

PyDSTool.Toolbox.NineML.frexp

PyDSTool.Toolbox.NineML.funcnames  

PyDSTool.Toolbox.NineML.genDB

PyDSTool.Toolbox.NineML.getdim  
 PyDSTool.Toolbox.NineML.getlftrtD  

PyDSTool.Toolbox.NineML.greater

PyDSTool.Toolbox.NineML.greater\_equal

PyDSTool.Toolbox.NineML.hypot

PyDSTool.Toolbox.NineML.index\_exp

PyDSTool.Toolbox.NineML.inf

PyDSTool.Toolbox.NineML.infty

PyDSTool.Toolbox.NineML.inverseMathNameMap

PyDSTool.Toolbox.NineML.invert

PyDSTool.Toolbox.NineML.isHierarchicalName  
 PyDSTool.Toolbox.NineML.isIntegerToken  
 PyDSTool.Toolbox.NineML.isNameToken  
 PyDSTool.Toolbox.NineML.isNumericToken  
 PyDSTool.Toolbox.NineML.isToken  
 PyDSTool.Toolbox.NineML.isVectorClause  

PyDSTool.Toolbox.NineML.isfinite

PyDSTool.Toolbox.NineML.isinf

PyDSTool.Toolbox.NineML.isnan

PyDSTool.Toolbox.NineML.joinAsStrs  
 PyDSTool.Toolbox.NineML.joinStrs  

PyDSTool.Toolbox.NineML.ldexp

PyDSTool.Toolbox.NineML.left\_shift

PyDSTool.Toolbox.NineML.less

PyDSTool.Toolbox.NineML.less\_equal

PyDSTool.Toolbox.NineML.little\_endian

PyDSTool.Toolbox.NineML.local\_fndef  

PyDSTool.Toolbox.NineML.log

PyDSTool.Toolbox.NineML.log10

PyDSTool.Toolbox.NineML.log1p

PyDSTool.Toolbox.NineML.logaddexp

PyDSTool.Toolbox.NineML.logaddexp2

PyDSTool.Toolbox.NineML.logical\_and

PyDSTool.Toolbox.NineML.logical\_not

PyDSTool.Toolbox.NineML.logical\_or

PyDSTool.Toolbox.NineML.logical\_xor

PyDSTool.Toolbox.NineML.makeParList  
 PyDSTool.Toolbox.NineML.mapNames  
 PyDSTool.Toolbox.NineML.mapPowStr  

PyDSTool.Toolbox.NineML.mathNameMap

PyDSTool.Toolbox.NineML.math\_dir  
 PyDSTool.Toolbox.NineML.math\_globals  
 PyDSTool.Toolbox.NineML.mathlookup  

PyDSTool.Toolbox.NineML.maximum

PyDSTool.Toolbox.NineML.mgrid

PyDSTool.Toolbox.NineML.minimum

PyDSTool.Toolbox.NineML.minpack  

PyDSTool.Toolbox.NineML.mod

PyDSTool.Toolbox.NineML.modf

PyDSTool.Toolbox.NineML.modlookup  

PyDSTool.Toolbox.NineML.multiply

PyDSTool.Toolbox.NineML.n

PyDSTool.Toolbox.NineML.nameResolver

PyDSTool.Toolbox.NineML.name\_chars\_RE  

PyDSTool.Toolbox.NineML.nan

PyDSTool.Toolbox.NineML.nbytes

PyDSTool.Toolbox.NineML.negative

PyDSTool.Toolbox.NineML.newaxis

PyDSTool.Toolbox.NineML.newton\_meth  

PyDSTool.Toolbox.NineML.nextafter

PyDSTool.Toolbox.NineML.norm  

PyDSTool.Toolbox.NineML.not\_equal

PyDSTool.Toolbox.NineML.notcontained

PyDSTool.Toolbox.NineML.null\_predicate

PyDSTool.Toolbox.NineML.null\_predicate\_class  
 PyDSTool.Toolbox.NineML.num\_chars  

PyDSTool.Toolbox.NineML.ogrid

PyDSTool.Toolbox.NineML.ones\_like

PyDSTool.Toolbox.NineML.parseMatrixStrToDictStr  
 PyDSTool.Toolbox.NineML.parser  
 PyDSTool.Toolbox.NineML.parserObject  

PyDSTool.Toolbox.NineML.pi

PyDSTool.Toolbox.NineML.power

PyDSTool.Toolbox.NineML.predicate\_op  
 PyDSTool.Toolbox.NineML.processMultiDef  
 PyDSTool.Toolbox.NineML.process\_raw\_residual  
 PyDSTool.Toolbox.NineML.proper\_match  

PyDSTool.Toolbox.NineML.protected\_allnames

PyDSTool.Toolbox.NineML.protected\_auxnamesDB

PyDSTool.Toolbox.NineML.protected\_macronames  
 PyDSTool.Toolbox.NineML.protected\_mathnames  
 PyDSTool.Toolbox.NineML.protected\_randomnames  
 PyDSTool.Toolbox.NineML.protected\_scipynames  
 PyDSTool.Toolbox.NineML.protected\_specialfns  
 PyDSTool.Toolbox.NineML.qtypes  

PyDSTool.Toolbox.NineML.r\_

PyDSTool.Toolbox.NineML.rad2deg

PyDSTool.Toolbox.NineML.radians

PyDSTool.Toolbox.NineML.randomlookup  
 PyDSTool.Toolbox.NineML.re  
 PyDSTool.Toolbox.NineML.readArgs  

PyDSTool.Toolbox.NineML.reciprocal

PyDSTool.Toolbox.NineML.regObject  

PyDSTool.Toolbox.NineML.remainder

PyDSTool.Toolbox.NineML.remove\_indices\_from\_range  
 PyDSTool.Toolbox.NineML.replaceCallsWithDummies  
 PyDSTool.Toolbox.NineML.replaceSep  
 PyDSTool.Toolbox.NineML.replaceSepInv  
 PyDSTool.Toolbox.NineML.replaceSepList  
 PyDSTool.Toolbox.NineML.replaceSepListInv  
 PyDSTool.Toolbox.NineML.replaceSepQSpec  
 PyDSTool.Toolbox.NineML.replaceSepStr  

PyDSTool.Toolbox.NineML.rerr

PyDSTool.Toolbox.NineML.reserved\_keywords  
 PyDSTool.Toolbox.NineML.resolveSpecTypeCombos  

PyDSTool.Toolbox.NineML.right\_shift

PyDSTool.Toolbox.NineML.rint

PyDSTool.Toolbox.NineML.rout

PyDSTool.Toolbox.NineML.s\_

PyDSTool.Toolbox.NineML.sctypeDict

PyDSTool.Toolbox.NineML.sctypeNA

PyDSTool.Toolbox.NineML.sctypes

PyDSTool.Toolbox.NineML.sign

PyDSTool.Toolbox.NineML.signbit

PyDSTool.Toolbox.NineML.simplify  
 PyDSTool.Toolbox.NineML.simplify\_str  

PyDSTool.Toolbox.NineML.sin

PyDSTool.Toolbox.NineML.sinh

PyDSTool.Toolbox.NineML.spacing

PyDSTool.Toolbox.NineML.specTypes

PyDSTool.Toolbox.NineML.specialfns  
 PyDSTool.Toolbox.NineML.splitargs  
 PyDSTool.Toolbox.NineML.splitastLR  

PyDSTool.Toolbox.NineML.sqrt

PyDSTool.Toolbox.NineML.square

PyDSTool.Toolbox.NineML.strIfSeq  
 PyDSTool.Toolbox.NineML.string2ast  

PyDSTool.Toolbox.NineML.subtract

PyDSTool.Toolbox.NineML.sym2name  
 PyDSTool.Toolbox.NineML.symbol  
 PyDSTool.Toolbox.NineML.symbolMapClass  

PyDSTool.Toolbox.NineML.symbolMapDict

PyDSTool.Toolbox.NineML.syms  

PyDSTool.Toolbox.NineML.t

PyDSTool.Toolbox.NineML.tan

PyDSTool.Toolbox.NineML.tanh

PyDSTool.Toolbox.NineML.targetLangs

PyDSTool.Toolbox.NineML.temp\_macro\_names  
 PyDSTool.Toolbox.NineML.temp\_macro\_names\_inv  

PyDSTool.Toolbox.NineML.theGenSpecHelper

PyDSTool.Toolbox.NineML.toCircumflexSyntax  
 PyDSTool.Toolbox.NineML.toDoubleStarSyntax  
 PyDSTool.Toolbox.NineML.toPowSyntax  
 PyDSTool.Toolbox.NineML.token  
 PyDSTool.Toolbox.NineML.traceback  

PyDSTool.Toolbox.NineML.true\_divide

PyDSTool.Toolbox.NineML.trunc

PyDSTool.Toolbox.NineML.trysimple  
 PyDSTool.Toolbox.NineML.typeCounter  

PyDSTool.Toolbox.NineML.typeDict

PyDSTool.Toolbox.NineML.typeNA

PyDSTool.Toolbox.NineML.typecodes

PyDSTool.Toolbox.NineML.uncertain

PyDSTool.Toolbox.NineML.utils\_info  

PyDSTool.Toolbox.NineML.verstr\_parts

PyDSTool.Toolbox.NineML.warnfields

PyDSTool.Toolbox.NineML.warnmessages

PyDSTool.Toolbox.NineML.whoQ  

PyDSTool.Toolbox.ParamEst.HAVE\_PSYCO

PyDSTool.Toolbox.ParamEst.Tester  

PyDSTool.Toolbox.ParamEst.\_ctn\_functions

PyDSTool.Toolbox.ParamEst.\_deprecated\_functions

PyDSTool.Toolbox.ParamEst.\_errors

PyDSTool.Toolbox.ParamEst.\_generic\_opt

PyDSTool.Toolbox.ParamEst.\_pest\_classes

PyDSTool.Toolbox.ParamEst.\_seq\_types

PyDSTool.Toolbox.ParamEst.\_utils

PyDSTool.Toolbox.ParamEst.power

PyDSTool.Toolbox.ParamEst.rerr

PyDSTool.Toolbox.ParamEst.res\_fn\_lookup

PyDSTool.Toolbox.ParamEst.rout

PyDSTool.Toolbox.ParamEst.sign

PyDSTool.Toolbox.ParamEst.solver\_lookup

PyDSTool.Toolbox.ParamEst.test  

PyDSTool.Toolbox.PySCes\_SBML.\_classes

PyDSTool.Toolbox.PySCes\_SBML.\_features

PyDSTool.Toolbox.PySCes\_SBML.\_functions

PyDSTool.Toolbox.PySCes\_SBML.\_num\_types

PyDSTool.Toolbox.PySCes\_SBML.\_seq\_types

PyDSTool.Toolbox.PySCes\_SBML.isfinite

PyDSTool.Toolbox.PySCes\_SBML.sign

PyDSTool.Toolbox.adjointPRC.ALLOW\_THREADS

PyDSTool.Toolbox.adjointPRC.Abs

PyDSTool.Toolbox.adjointPRC.Acos

PyDSTool.Toolbox.adjointPRC.Asin

PyDSTool.Toolbox.adjointPRC.Atan

PyDSTool.Toolbox.adjointPRC.Atan2

PyDSTool.Toolbox.adjointPRC.BUFSIZE

PyDSTool.Toolbox.adjointPRC.Betavariate

PyDSTool.Toolbox.adjointPRC.CLIP

PyDSTool.Toolbox.adjointPRC.Ceil

PyDSTool.Toolbox.adjointPRC.Choice

PyDSTool.Toolbox.adjointPRC.Cos

PyDSTool.Toolbox.adjointPRC.Cosh

PyDSTool.Toolbox.adjointPRC.Degrees

PyDSTool.Toolbox.adjointPRC.E

PyDSTool.Toolbox.adjointPRC.ERR\_CALL

PyDSTool.Toolbox.adjointPRC.ERR\_DEFAULT

PyDSTool.Toolbox.adjointPRC.ERR\_DEFAULT2

PyDSTool.Toolbox.adjointPRC.ERR\_IGNORE

PyDSTool.Toolbox.adjointPRC.ERR\_LOG

PyDSTool.Toolbox.adjointPRC.ERR\_PRINT

PyDSTool.Toolbox.adjointPRC.ERR\_RAISE

PyDSTool.Toolbox.adjointPRC.ERR\_WARN

PyDSTool.Toolbox.adjointPRC.Exp

PyDSTool.Toolbox.adjointPRC.Expovariate

PyDSTool.Toolbox.adjointPRC.FLOATING\_POINT\_SUPPORT

PyDSTool.Toolbox.adjointPRC.FPE\_DIVIDEBYZERO

PyDSTool.Toolbox.adjointPRC.FPE\_INVALID

PyDSTool.Toolbox.adjointPRC.FPE\_OVERFLOW

PyDSTool.Toolbox.adjointPRC.FPE\_UNDERFLOW

PyDSTool.Toolbox.adjointPRC.Fabs

PyDSTool.Toolbox.adjointPRC.False\_

PyDSTool.Toolbox.adjointPRC.Floor

PyDSTool.Toolbox.adjointPRC.Fmod

PyDSTool.Toolbox.adjointPRC.Frexp

PyDSTool.Toolbox.adjointPRC.Gammavariate

PyDSTool.Toolbox.adjointPRC.Gauss

PyDSTool.Toolbox.adjointPRC.Getrandbits

PyDSTool.Toolbox.adjointPRC.Getstate

PyDSTool.Toolbox.adjointPRC.Hypot

PyDSTool.Toolbox.adjointPRC.Infinity

PyDSTool.Toolbox.adjointPRC.Jumpahead

PyDSTool.Toolbox.adjointPRC.Ldexp

PyDSTool.Toolbox.adjointPRC.Log

PyDSTool.Toolbox.adjointPRC.Log10

PyDSTool.Toolbox.adjointPRC.Lognormvariate

PyDSTool.Toolbox.adjointPRC.MAXDIMS

PyDSTool.Toolbox.adjointPRC.Max

PyDSTool.Toolbox.adjointPRC.Min

PyDSTool.Toolbox.adjointPRC.Modf

PyDSTool.Toolbox.adjointPRC.NAN

PyDSTool.Toolbox.adjointPRC.NINF

PyDSTool.Toolbox.adjointPRC.NZERO

PyDSTool.Toolbox.adjointPRC.Normalvariate

PyDSTool.Toolbox.adjointPRC.PINF

PyDSTool.Toolbox.adjointPRC.PZERO

PyDSTool.Toolbox.adjointPRC.Paretovariate

PyDSTool.Toolbox.adjointPRC.Pi

PyDSTool.Toolbox.adjointPRC.Pow

PyDSTool.Toolbox.adjointPRC.RAISE

PyDSTool.Toolbox.adjointPRC.Radians

PyDSTool.Toolbox.adjointPRC.Randint

PyDSTool.Toolbox.adjointPRC.Random

PyDSTool.Toolbox.adjointPRC.Randrange

PyDSTool.Toolbox.adjointPRC.SHIFT\_DIVIDEBYZERO

PyDSTool.Toolbox.adjointPRC.SHIFT\_INVALID

PyDSTool.Toolbox.adjointPRC.SHIFT\_OVERFLOW

PyDSTool.Toolbox.adjointPRC.SHIFT\_UNDERFLOW

PyDSTool.Toolbox.adjointPRC.Sample

PyDSTool.Toolbox.adjointPRC.ScalarType

PyDSTool.Toolbox.adjointPRC.Seed

PyDSTool.Toolbox.adjointPRC.Setstate

PyDSTool.Toolbox.adjointPRC.Shuffle

PyDSTool.Toolbox.adjointPRC.Sin

PyDSTool.Toolbox.adjointPRC.Sinh

PyDSTool.Toolbox.adjointPRC.Sqrt

PyDSTool.Toolbox.adjointPRC.Sum

PyDSTool.Toolbox.adjointPRC.Systemrandom

PyDSTool.Toolbox.adjointPRC.Tan

PyDSTool.Toolbox.adjointPRC.Tanh

PyDSTool.Toolbox.adjointPRC.True\_

PyDSTool.Toolbox.adjointPRC.UFUNC\_BUFSIZE\_DEFAULT

PyDSTool.Toolbox.adjointPRC.UFUNC\_PYVALS\_NAME

PyDSTool.Toolbox.adjointPRC.Uniform

PyDSTool.Toolbox.adjointPRC.Vonmisesvariate

PyDSTool.Toolbox.adjointPRC.WRAP

PyDSTool.Toolbox.adjointPRC.Weibullvariate

PyDSTool.Toolbox.adjointPRC.Wichmannhill

PyDSTool.Toolbox.adjointPRC.absolute

PyDSTool.Toolbox.adjointPRC.add

PyDSTool.Toolbox.adjointPRC.bitwise\_and

PyDSTool.Toolbox.adjointPRC.bitwise\_not

PyDSTool.Toolbox.adjointPRC.bitwise\_or

PyDSTool.Toolbox.adjointPRC.bitwise\_xor

PyDSTool.Toolbox.adjointPRC.c\_

PyDSTool.Toolbox.adjointPRC.cast

PyDSTool.Toolbox.adjointPRC.conj

PyDSTool.Toolbox.adjointPRC.conjugate

PyDSTool.Toolbox.adjointPRC.copysign

PyDSTool.Toolbox.adjointPRC.deg2rad

PyDSTool.Toolbox.adjointPRC.divide

PyDSTool.Toolbox.adjointPRC.equal

PyDSTool.Toolbox.adjointPRC.exp2

PyDSTool.Toolbox.adjointPRC.expm1

PyDSTool.Toolbox.adjointPRC.floor\_divide

PyDSTool.Toolbox.adjointPRC.fmax

PyDSTool.Toolbox.adjointPRC.fmin

PyDSTool.Toolbox.adjointPRC.greater\_equal

PyDSTool.Toolbox.adjointPRC.index\_exp

PyDSTool.Toolbox.adjointPRC.inf

PyDSTool.Toolbox.adjointPRC.infty

PyDSTool.Toolbox.adjointPRC.invert

PyDSTool.Toolbox.adjointPRC.isinf

PyDSTool.Toolbox.adjointPRC.left\_shift

PyDSTool.Toolbox.adjointPRC.little\_endian

PyDSTool.Toolbox.adjointPRC.log1p

PyDSTool.Toolbox.adjointPRC.logaddexp

PyDSTool.Toolbox.adjointPRC.logaddexp2

PyDSTool.Toolbox.adjointPRC.logical\_and

PyDSTool.Toolbox.adjointPRC.logical\_not

PyDSTool.Toolbox.adjointPRC.logical\_xor

PyDSTool.Toolbox.adjointPRC.maximum

PyDSTool.Toolbox.adjointPRC.mgrid

PyDSTool.Toolbox.adjointPRC.minimum

PyDSTool.Toolbox.adjointPRC.multiply

PyDSTool.Toolbox.adjointPRC.n

PyDSTool.Toolbox.adjointPRC.nan

PyDSTool.Toolbox.adjointPRC.nbytes

PyDSTool.Toolbox.adjointPRC.negative

PyDSTool.Toolbox.adjointPRC.newaxis

PyDSTool.Toolbox.adjointPRC.nextafter

PyDSTool.Toolbox.adjointPRC.not\_equal

PyDSTool.Toolbox.adjointPRC.ogrid

PyDSTool.Toolbox.adjointPRC.ones\_like

PyDSTool.Toolbox.adjointPRC.r\_

PyDSTool.Toolbox.adjointPRC.rad2deg

PyDSTool.Toolbox.adjointPRC.reciprocal

PyDSTool.Toolbox.adjointPRC.remainder

PyDSTool.Toolbox.adjointPRC.right\_shift

PyDSTool.Toolbox.adjointPRC.rint

PyDSTool.Toolbox.adjointPRC.s\_

PyDSTool.Toolbox.adjointPRC.sctypeDict

PyDSTool.Toolbox.adjointPRC.sctypeNA

PyDSTool.Toolbox.adjointPRC.sctypes

PyDSTool.Toolbox.adjointPRC.signbit

PyDSTool.Toolbox.adjointPRC.spacing

PyDSTool.Toolbox.adjointPRC.square

PyDSTool.Toolbox.adjointPRC.subtract

PyDSTool.Toolbox.adjointPRC.t

PyDSTool.Toolbox.adjointPRC.true\_divide

PyDSTool.Toolbox.adjointPRC.trunc

PyDSTool.Toolbox.adjointPRC.typeDict

PyDSTool.Toolbox.adjointPRC.typeNA

PyDSTool.Toolbox.adjointPRC.typecodes

PyDSTool.Toolbox.data\_analysis.\_PCA\_utils

PyDSTool.Toolbox.data\_analysis.\_analysis\_utils

PyDSTool.Toolbox.data\_analysis.\_filter\_utils

PyDSTool.Toolbox.data\_analysis.\_fit\_utils

PyDSTool.Toolbox.data\_analysis.\_int\_types

PyDSTool.Toolbox.data\_analysis.\_metric\_utils

PyDSTool.Toolbox.data\_analysis.\_num\_types

PyDSTool.Toolbox.data\_analysis.\_seq\_types

PyDSTool.Toolbox.data\_analysis.dict\_tol

PyDSTool.Toolbox.data\_analysis.newaxis

PyDSTool.Toolbox.data\_analysis.r\_

PyDSTool.Toolbox.dataanalysis.ALLOW\_THREADS

PyDSTool.Toolbox.dataanalysis.API

PyDSTool.Toolbox.dataanalysis.API\_class  

PyDSTool.Toolbox.dataanalysis.Abs

PyDSTool.Toolbox.dataanalysis.Acos

PyDSTool.Toolbox.dataanalysis.Asin

PyDSTool.Toolbox.dataanalysis.Atan

PyDSTool.Toolbox.dataanalysis.Atan2

PyDSTool.Toolbox.dataanalysis.BUFSIZE

PyDSTool.Toolbox.dataanalysis.Betavariate

PyDSTool.Toolbox.dataanalysis.CLIP

PyDSTool.Toolbox.dataanalysis.Ceil

PyDSTool.Toolbox.dataanalysis.Choice

PyDSTool.Toolbox.dataanalysis.Continuous

PyDSTool.Toolbox.dataanalysis.Cos

PyDSTool.Toolbox.dataanalysis.Cosh

PyDSTool.Toolbox.dataanalysis.DO\_DEC  
 PyDSTool.Toolbox.dataanalysis.DO\_POW  

PyDSTool.Toolbox.dataanalysis.Degrees

PyDSTool.Toolbox.dataanalysis.Discrete

PyDSTool.Toolbox.dataanalysis.DomainType  

PyDSTool.Toolbox.dataanalysis.E

PyDSTool.Toolbox.dataanalysis.ERR\_CALL

PyDSTool.Toolbox.dataanalysis.ERR\_DEFAULT

PyDSTool.Toolbox.dataanalysis.ERR\_DEFAULT2

PyDSTool.Toolbox.dataanalysis.ERR\_IGNORE

PyDSTool.Toolbox.dataanalysis.ERR\_LOG

PyDSTool.Toolbox.dataanalysis.ERR\_PRINT

PyDSTool.Toolbox.dataanalysis.ERR\_RAISE

PyDSTool.Toolbox.dataanalysis.ERR\_WARN

PyDSTool.Toolbox.dataanalysis.E\_COMPUTFAIL

PyDSTool.Toolbox.dataanalysis.E\_NONUNIQUETERM

PyDSTool.Toolbox.dataanalysis.Exp

PyDSTool.Toolbox.dataanalysis.Expovariate

PyDSTool.Toolbox.dataanalysis.FLOATING\_POINT\_SUPPORT

PyDSTool.Toolbox.dataanalysis.FPE\_DIVIDEBYZERO

PyDSTool.Toolbox.dataanalysis.FPE\_INVALID

PyDSTool.Toolbox.dataanalysis.FPE\_OVERFLOW

PyDSTool.Toolbox.dataanalysis.FPE\_UNDERFLOW

PyDSTool.Toolbox.dataanalysis.Fabs

PyDSTool.Toolbox.dataanalysis.False\_

PyDSTool.Toolbox.dataanalysis.Floor

PyDSTool.Toolbox.dataanalysis.Fmod

PyDSTool.Toolbox.dataanalysis.Frexp

PyDSTool.Toolbox.dataanalysis.Gammavariate

PyDSTool.Toolbox.dataanalysis.Gauss

PyDSTool.Toolbox.dataanalysis.Getrandbits

PyDSTool.Toolbox.dataanalysis.Getstate

PyDSTool.Toolbox.dataanalysis.HAVE\_PSYCO

PyDSTool.Toolbox.dataanalysis.Hypot

PyDSTool.Toolbox.dataanalysis.Inf

PyDSTool.Toolbox.dataanalysis.Infinity

PyDSTool.Toolbox.dataanalysis.Jumpahead

PyDSTool.Toolbox.dataanalysis.LargestInt32

PyDSTool.Toolbox.dataanalysis.Ldexp

PyDSTool.Toolbox.dataanalysis.Log

PyDSTool.Toolbox.dataanalysis.Log10

PyDSTool.Toolbox.dataanalysis.Lognormvariate

PyDSTool.Toolbox.dataanalysis.MAXDIMS

PyDSTool.Toolbox.dataanalysis.MReg  
 PyDSTool.Toolbox.dataanalysis.Macheps  

PyDSTool.Toolbox.dataanalysis.Max

PyDSTool.Toolbox.dataanalysis.Min

PyDSTool.Toolbox.dataanalysis.Modf

PyDSTool.Toolbox.dataanalysis.NAMESEP  

PyDSTool.Toolbox.dataanalysis.NAN

PyDSTool.Toolbox.dataanalysis.NINF

PyDSTool.Toolbox.dataanalysis.NZERO

PyDSTool.Toolbox.dataanalysis.NaN

PyDSTool.Toolbox.dataanalysis.Normalvariate

PyDSTool.Toolbox.dataanalysis.ONES  

PyDSTool.Toolbox.dataanalysis.PCANode

PyDSTool.Toolbox.dataanalysis.PINF

PyDSTool.Toolbox.dataanalysis.POW\_STR  

PyDSTool.Toolbox.dataanalysis.PZERO

PyDSTool.Toolbox.dataanalysis.Paretovariate

PyDSTool.Toolbox.dataanalysis.Pi

PyDSTool.Toolbox.dataanalysis.Pow

PyDSTool.Toolbox.dataanalysis.RAISE

PyDSTool.Toolbox.dataanalysis.Radians

PyDSTool.Toolbox.dataanalysis.Randint

PyDSTool.Toolbox.dataanalysis.Random

PyDSTool.Toolbox.dataanalysis.Randrange

PyDSTool.Toolbox.dataanalysis.SHIFT\_DIVIDEBYZERO

PyDSTool.Toolbox.dataanalysis.SHIFT\_INVALID

PyDSTool.Toolbox.dataanalysis.SHIFT\_OVERFLOW

PyDSTool.Toolbox.dataanalysis.SHIFT\_UNDERFLOW

PyDSTool.Toolbox.dataanalysis.Sample

PyDSTool.Toolbox.dataanalysis.ScalarType

PyDSTool.Toolbox.dataanalysis.Seed

PyDSTool.Toolbox.dataanalysis.Setstate

PyDSTool.Toolbox.dataanalysis.Shuffle

PyDSTool.Toolbox.dataanalysis.Sin

PyDSTool.Toolbox.dataanalysis.Sinh

PyDSTool.Toolbox.dataanalysis.Sqrt

PyDSTool.Toolbox.dataanalysis.Sum

PyDSTool.Toolbox.dataanalysis.Systemrandom

PyDSTool.Toolbox.dataanalysis.TENS  

PyDSTool.Toolbox.dataanalysis.Tan

PyDSTool.Toolbox.dataanalysis.Tanh

PyDSTool.Toolbox.dataanalysis.True\_

PyDSTool.Toolbox.dataanalysis.UFUNC\_BUFSIZE\_DEFAULT

PyDSTool.Toolbox.dataanalysis.UFUNC\_PYVALS\_NAME

PyDSTool.Toolbox.dataanalysis.Uniform

PyDSTool.Toolbox.dataanalysis.Vonmisesvariate

PyDSTool.Toolbox.dataanalysis.WRAP

PyDSTool.Toolbox.dataanalysis.W\_BISECTLIMIT

PyDSTool.Toolbox.dataanalysis.W\_NONTERMEVENT

PyDSTool.Toolbox.dataanalysis.W\_NONTERMSTATEBD

PyDSTool.Toolbox.dataanalysis.W\_TERMEVENT

PyDSTool.Toolbox.dataanalysis.W\_TERMSTATEBD

PyDSTool.Toolbox.dataanalysis.W\_UNCERTVAL

PyDSTool.Toolbox.dataanalysis.Weibullvariate

PyDSTool.Toolbox.dataanalysis.Wichmannhill

PyDSTool.Toolbox.dataanalysis.ZEROS  

PyDSTool.Toolbox.dataanalysis.\_PCA\_utils

PyDSTool.Toolbox.dataanalysis.\_analysis\_utils

PyDSTool.Toolbox.dataanalysis.\_fit\_utils

PyDSTool.Toolbox.dataanalysis.\_int\_types

PyDSTool.Toolbox.dataanalysis.\_metric\_utils

PyDSTool.Toolbox.dataanalysis.\_num\_types

PyDSTool.Toolbox.dataanalysis.\_seq\_types

PyDSTool.Toolbox.dataanalysis.absolute

PyDSTool.Toolbox.dataanalysis.accrueCompTypes  
 PyDSTool.Toolbox.dataanalysis.acosh  

PyDSTool.Toolbox.dataanalysis.add

PyDSTool.Toolbox.dataanalysis.allCompTypes  

PyDSTool.Toolbox.dataanalysis.allmathnames

PyDSTool.Toolbox.dataanalysis.allmathnames\_symbolic

PyDSTool.Toolbox.dataanalysis.alphabet\_chars\_RE  

PyDSTool.Toolbox.dataanalysis.arccos

PyDSTool.Toolbox.dataanalysis.arccosh

PyDSTool.Toolbox.dataanalysis.arcsin

PyDSTool.Toolbox.dataanalysis.arcsinh

PyDSTool.Toolbox.dataanalysis.arctan

PyDSTool.Toolbox.dataanalysis.arctan2

PyDSTool.Toolbox.dataanalysis.arctanh

PyDSTool.Toolbox.dataanalysis.asinh  
 PyDSTool.Toolbox.dataanalysis.ast2shortlist  
 PyDSTool.Toolbox.dataanalysis.ast2string  
 PyDSTool.Toolbox.dataanalysis.atanh  

PyDSTool.Toolbox.dataanalysis.bitwise\_and

PyDSTool.Toolbox.dataanalysis.bitwise\_not

PyDSTool.Toolbox.dataanalysis.bitwise\_or

PyDSTool.Toolbox.dataanalysis.bitwise\_xor

PyDSTool.Toolbox.dataanalysis.builtinFnSigInfo  
 PyDSTool.Toolbox.dataanalysis.builtin\_auxnames  
 PyDSTool.Toolbox.dataanalysis.builtinlookup  
 PyDSTool.Toolbox.dataanalysis.cPickle  

PyDSTool.Toolbox.dataanalysis.c\_

PyDSTool.Toolbox.dataanalysis.cast

PyDSTool.Toolbox.dataanalysis.ceil

PyDSTool.Toolbox.dataanalysis.collect\_numbers  
 PyDSTool.Toolbox.dataanalysis.complex  
 PyDSTool.Toolbox.dataanalysis.complex192  

PyDSTool.Toolbox.dataanalysis.conj

PyDSTool.Toolbox.dataanalysis.conjugate

PyDSTool.Toolbox.dataanalysis.contained

PyDSTool.Toolbox.dataanalysis.convert\_power\_reserved\_keywords  

PyDSTool.Toolbox.dataanalysis.copysign

PyDSTool.Toolbox.dataanalysis.cos

PyDSTool.Toolbox.dataanalysis.cosh

PyDSTool.Toolbox.dataanalysis.count\_sep  

PyDSTool.Toolbox.dataanalysis.deg2rad

PyDSTool.Toolbox.dataanalysis.degrees

PyDSTool.Toolbox.dataanalysis.dict\_tol

PyDSTool.Toolbox.dataanalysis.divide

PyDSTool.Toolbox.dataanalysis.doadd  
 PyDSTool.Toolbox.dataanalysis.dodiv  
 PyDSTool.Toolbox.dataanalysis.dofun  
 PyDSTool.Toolbox.dataanalysis.domul  
 PyDSTool.Toolbox.dataanalysis.doneg  
 PyDSTool.Toolbox.dataanalysis.dopower  
 PyDSTool.Toolbox.dataanalysis.dosub  
 PyDSTool.Toolbox.dataanalysis.dsInterface  

PyDSTool.Toolbox.dataanalysis.e

PyDSTool.Toolbox.dataanalysis.ensurebare  
 PyDSTool.Toolbox.dataanalysis.ensuredecimalconst  
 PyDSTool.Toolbox.dataanalysis.ensureints  
 PyDSTool.Toolbox.dataanalysis.ensureparen  
 PyDSTool.Toolbox.dataanalysis.ensureparen\_div  

PyDSTool.Toolbox.dataanalysis.equal

PyDSTool.Toolbox.dataanalysis.errmessages

PyDSTool.Toolbox.dataanalysis.errorfields

PyDSTool.Toolbox.dataanalysis.exp

PyDSTool.Toolbox.dataanalysis.exp2

PyDSTool.Toolbox.dataanalysis.expm1

PyDSTool.Toolbox.dataanalysis.fabs

PyDSTool.Toolbox.dataanalysis.feature\_leaf  
 PyDSTool.Toolbox.dataanalysis.feature\_node  
 PyDSTool.Toolbox.dataanalysis.feval\_map\_const  
 PyDSTool.Toolbox.dataanalysis.feval\_map\_symb  
 PyDSTool.Toolbox.dataanalysis.findEndBrace  
 PyDSTool.Toolbox.dataanalysis.findNumTailPos  
 PyDSTool.Toolbox.dataanalysis.fit\_function  
 PyDSTool.Toolbox.dataanalysis.float96  

PyDSTool.Toolbox.dataanalysis.floor

PyDSTool.Toolbox.dataanalysis.floor\_divide

PyDSTool.Toolbox.dataanalysis.fmax

PyDSTool.Toolbox.dataanalysis.fmin

PyDSTool.Toolbox.dataanalysis.fmod

PyDSTool.Toolbox.dataanalysis.frexp

PyDSTool.Toolbox.dataanalysis.funcnames  

PyDSTool.Toolbox.dataanalysis.genDB

PyDSTool.Toolbox.dataanalysis.getdim  
 PyDSTool.Toolbox.dataanalysis.getlftrtD  

PyDSTool.Toolbox.dataanalysis.greater

PyDSTool.Toolbox.dataanalysis.greater\_equal

PyDSTool.Toolbox.dataanalysis.hypot

PyDSTool.Toolbox.dataanalysis.index\_exp

PyDSTool.Toolbox.dataanalysis.inf

PyDSTool.Toolbox.dataanalysis.infty

PyDSTool.Toolbox.dataanalysis.inverseMathNameMap

PyDSTool.Toolbox.dataanalysis.invert

PyDSTool.Toolbox.dataanalysis.isHierarchicalName  
 PyDSTool.Toolbox.dataanalysis.isIntegerToken  
 PyDSTool.Toolbox.dataanalysis.isNameToken  
 PyDSTool.Toolbox.dataanalysis.isNumericToken  
 PyDSTool.Toolbox.dataanalysis.isToken  
 PyDSTool.Toolbox.dataanalysis.isVectorClause  

PyDSTool.Toolbox.dataanalysis.isfinite

PyDSTool.Toolbox.dataanalysis.isinf

PyDSTool.Toolbox.dataanalysis.isnan

PyDSTool.Toolbox.dataanalysis.joinAsStrs  
 PyDSTool.Toolbox.dataanalysis.joinStrs  

PyDSTool.Toolbox.dataanalysis.ldexp

PyDSTool.Toolbox.dataanalysis.left\_shift

PyDSTool.Toolbox.dataanalysis.less

PyDSTool.Toolbox.dataanalysis.less\_equal

PyDSTool.Toolbox.dataanalysis.little\_endian

PyDSTool.Toolbox.dataanalysis.local\_fndef  

PyDSTool.Toolbox.dataanalysis.log

PyDSTool.Toolbox.dataanalysis.log10

PyDSTool.Toolbox.dataanalysis.log1p

PyDSTool.Toolbox.dataanalysis.logaddexp

PyDSTool.Toolbox.dataanalysis.logaddexp2

PyDSTool.Toolbox.dataanalysis.logical\_and

PyDSTool.Toolbox.dataanalysis.logical\_not

PyDSTool.Toolbox.dataanalysis.logical\_or

PyDSTool.Toolbox.dataanalysis.logical\_xor

PyDSTool.Toolbox.dataanalysis.makeParList  
 PyDSTool.Toolbox.dataanalysis.mapNames  
 PyDSTool.Toolbox.dataanalysis.mapPowStr  

PyDSTool.Toolbox.dataanalysis.mathNameMap

PyDSTool.Toolbox.dataanalysis.math\_dir  
 PyDSTool.Toolbox.dataanalysis.math\_globals  
 PyDSTool.Toolbox.dataanalysis.mathlookup  

PyDSTool.Toolbox.dataanalysis.maximum

PyDSTool.Toolbox.dataanalysis.mgrid

PyDSTool.Toolbox.dataanalysis.minimum

PyDSTool.Toolbox.dataanalysis.minpack  

PyDSTool.Toolbox.dataanalysis.mod

PyDSTool.Toolbox.dataanalysis.modf

PyDSTool.Toolbox.dataanalysis.modlookup  

PyDSTool.Toolbox.dataanalysis.multiply

PyDSTool.Toolbox.dataanalysis.n

PyDSTool.Toolbox.dataanalysis.nameResolver

PyDSTool.Toolbox.dataanalysis.name\_chars\_RE  

PyDSTool.Toolbox.dataanalysis.nan

PyDSTool.Toolbox.dataanalysis.nbytes

PyDSTool.Toolbox.dataanalysis.negative

PyDSTool.Toolbox.dataanalysis.newaxis

PyDSTool.Toolbox.dataanalysis.newton\_meth  

PyDSTool.Toolbox.dataanalysis.nextafter

PyDSTool.Toolbox.dataanalysis.not\_equal

PyDSTool.Toolbox.dataanalysis.notcontained

PyDSTool.Toolbox.dataanalysis.null\_predicate

PyDSTool.Toolbox.dataanalysis.null\_predicate\_class  
 PyDSTool.Toolbox.dataanalysis.num\_chars  

PyDSTool.Toolbox.dataanalysis.ogrid

PyDSTool.Toolbox.dataanalysis.ones\_like

PyDSTool.Toolbox.dataanalysis.parseMatrixStrToDictStr  
 PyDSTool.Toolbox.dataanalysis.parser  
 PyDSTool.Toolbox.dataanalysis.parserObject  

PyDSTool.Toolbox.dataanalysis.pi

PyDSTool.Toolbox.dataanalysis.power

PyDSTool.Toolbox.dataanalysis.predicate\_op  
 PyDSTool.Toolbox.dataanalysis.processMultiDef  
 PyDSTool.Toolbox.dataanalysis.process\_raw\_residual  
 PyDSTool.Toolbox.dataanalysis.proper\_match  

PyDSTool.Toolbox.dataanalysis.protected\_allnames

PyDSTool.Toolbox.dataanalysis.protected\_auxnamesDB

PyDSTool.Toolbox.dataanalysis.protected\_macronames  
 PyDSTool.Toolbox.dataanalysis.protected\_mathnames  
 PyDSTool.Toolbox.dataanalysis.protected\_randomnames  
 PyDSTool.Toolbox.dataanalysis.protected\_scipynames  
 PyDSTool.Toolbox.dataanalysis.protected\_specialfns  
 PyDSTool.Toolbox.dataanalysis.qtypes  

PyDSTool.Toolbox.dataanalysis.r\_

PyDSTool.Toolbox.dataanalysis.rad2deg

PyDSTool.Toolbox.dataanalysis.radians

PyDSTool.Toolbox.dataanalysis.randomlookup  
 PyDSTool.Toolbox.dataanalysis.re  
 PyDSTool.Toolbox.dataanalysis.readArgs  

PyDSTool.Toolbox.dataanalysis.reciprocal

PyDSTool.Toolbox.dataanalysis.regObject  

PyDSTool.Toolbox.dataanalysis.remainder

PyDSTool.Toolbox.dataanalysis.remove\_indices\_from\_range  
 PyDSTool.Toolbox.dataanalysis.replaceCallsWithDummies  
 PyDSTool.Toolbox.dataanalysis.replaceSep  
 PyDSTool.Toolbox.dataanalysis.replaceSepInv  
 PyDSTool.Toolbox.dataanalysis.replaceSepList  
 PyDSTool.Toolbox.dataanalysis.replaceSepListInv  
 PyDSTool.Toolbox.dataanalysis.replaceSepQSpec  
 PyDSTool.Toolbox.dataanalysis.replaceSepStr  

PyDSTool.Toolbox.dataanalysis.rerr

PyDSTool.Toolbox.dataanalysis.reserved\_keywords  
 PyDSTool.Toolbox.dataanalysis.resolveSpecTypeCombos  

PyDSTool.Toolbox.dataanalysis.right\_shift

PyDSTool.Toolbox.dataanalysis.rint

PyDSTool.Toolbox.dataanalysis.rout

PyDSTool.Toolbox.dataanalysis.s\_

PyDSTool.Toolbox.dataanalysis.sctypeDict

PyDSTool.Toolbox.dataanalysis.sctypeNA

PyDSTool.Toolbox.dataanalysis.sctypes

PyDSTool.Toolbox.dataanalysis.sign

PyDSTool.Toolbox.dataanalysis.signbit

PyDSTool.Toolbox.dataanalysis.simplify  
 PyDSTool.Toolbox.dataanalysis.simplify\_str  

PyDSTool.Toolbox.dataanalysis.sin

PyDSTool.Toolbox.dataanalysis.sinh

PyDSTool.Toolbox.dataanalysis.spacing

PyDSTool.Toolbox.dataanalysis.specTypes

PyDSTool.Toolbox.dataanalysis.specialfns  
 PyDSTool.Toolbox.dataanalysis.splitargs  
 PyDSTool.Toolbox.dataanalysis.splitastLR  

PyDSTool.Toolbox.dataanalysis.sqrt

PyDSTool.Toolbox.dataanalysis.square

PyDSTool.Toolbox.dataanalysis.strIfSeq  
 PyDSTool.Toolbox.dataanalysis.string2ast  

PyDSTool.Toolbox.dataanalysis.subtract

PyDSTool.Toolbox.dataanalysis.sym2name  
 PyDSTool.Toolbox.dataanalysis.symbol  
 PyDSTool.Toolbox.dataanalysis.symbolMapClass  

PyDSTool.Toolbox.dataanalysis.symbolMapDict

PyDSTool.Toolbox.dataanalysis.syms  

PyDSTool.Toolbox.dataanalysis.t

PyDSTool.Toolbox.dataanalysis.tan

PyDSTool.Toolbox.dataanalysis.tanh

PyDSTool.Toolbox.dataanalysis.targetLangs

PyDSTool.Toolbox.dataanalysis.temp\_macro\_names  
 PyDSTool.Toolbox.dataanalysis.temp\_macro\_names\_inv  

PyDSTool.Toolbox.dataanalysis.theGenSpecHelper

PyDSTool.Toolbox.dataanalysis.toCircumflexSyntax  
 PyDSTool.Toolbox.dataanalysis.toDoubleStarSyntax  
 PyDSTool.Toolbox.dataanalysis.toPowSyntax  
 PyDSTool.Toolbox.dataanalysis.token  
 PyDSTool.Toolbox.dataanalysis.traceback  

PyDSTool.Toolbox.dataanalysis.true\_divide

PyDSTool.Toolbox.dataanalysis.trunc

PyDSTool.Toolbox.dataanalysis.trysimple  
 PyDSTool.Toolbox.dataanalysis.typeCounter  

PyDSTool.Toolbox.dataanalysis.typeDict

PyDSTool.Toolbox.dataanalysis.typeNA

PyDSTool.Toolbox.dataanalysis.typecodes

PyDSTool.Toolbox.dataanalysis.uncertain

PyDSTool.Toolbox.dataanalysis.utils\_info  

PyDSTool.Toolbox.dataanalysis.verstr\_parts

PyDSTool.Toolbox.dataanalysis.warnfields

PyDSTool.Toolbox.dataanalysis.warnmessages

PyDSTool.Toolbox.dataanalysis.whoQ  
 PyDSTool.Toolbox.dssrt.APPROX  
 PyDSTool.Toolbox.dssrt.CLOSE  
 PyDSTool.Toolbox.dssrt.FAR  
 PyDSTool.Toolbox.dssrt.GAP  

PyDSTool.Toolbox.dssrt.JARO\_MARKER\_CHAR

PyDSTool.Toolbox.dssrt.MATCH  
 PyDSTool.Toolbox.dssrt.MISMATCH  
 PyDSTool.Toolbox.dssrt.VCLOSE  
 PyDSTool.Toolbox.dssrt.VFAR  

PyDSTool.Toolbox.dssrt.\_classes

PyDSTool.Toolbox.dssrt.\_functions

PyDSTool.Toolbox.dssrt.\_predicates

PyDSTool.Toolbox.event\_driven\_simulator.API

PyDSTool.Toolbox.event\_driven\_simulator.API\_class  

PyDSTool.Toolbox.event\_driven\_simulator.Continuous

PyDSTool.Toolbox.event\_driven\_simulator.Discrete

PyDSTool.Toolbox.event\_driven\_simulator.DomainType  

PyDSTool.Toolbox.event\_driven\_simulator.Inf

PyDSTool.Toolbox.event\_driven\_simulator.LargestInt32

PyDSTool.Toolbox.event\_driven\_simulator.Macheps  

PyDSTool.Toolbox.event\_driven\_simulator.NaN

PyDSTool.Toolbox.event\_driven\_simulator.\_all\_complex

PyDSTool.Toolbox.event\_driven\_simulator.\_all\_float

PyDSTool.Toolbox.event\_driven\_simulator.\_all\_int

PyDSTool.Toolbox.event\_driven\_simulator.\_all\_numpy\_complex

PyDSTool.Toolbox.event\_driven\_simulator.\_all\_numpy\_float

PyDSTool.Toolbox.event\_driven\_simulator.\_all\_numpy\_int

PyDSTool.Toolbox.event\_driven\_simulator.\_classes

PyDSTool.Toolbox.event\_driven\_simulator.\_complex\_types

PyDSTool.Toolbox.event\_driven\_simulator.\_float\_types

PyDSTool.Toolbox.event\_driven\_simulator.\_functions

PyDSTool.Toolbox.event\_driven\_simulator.\_instances

PyDSTool.Toolbox.event\_driven\_simulator.\_int\_types

PyDSTool.Toolbox.event\_driven\_simulator.\_num\_equivtype

PyDSTool.Toolbox.event\_driven\_simulator.\_num\_maxmin

PyDSTool.Toolbox.event\_driven\_simulator.\_num\_name2equivtypes

PyDSTool.Toolbox.event\_driven\_simulator.\_num\_name2type

PyDSTool.Toolbox.event\_driven\_simulator.\_num\_type2name

PyDSTool.Toolbox.event\_driven\_simulator.\_num\_types

PyDSTool.Toolbox.event\_driven\_simulator.\_pytypefromtype

PyDSTool.Toolbox.event\_driven\_simulator.\_real\_types

PyDSTool.Toolbox.event\_driven\_simulator.\_seq\_types

PyDSTool.Toolbox.event\_driven\_simulator.all  
 PyDSTool.Toolbox.event\_driven\_simulator.alltrue  
 PyDSTool.Toolbox.event\_driven\_simulator.any  
 PyDSTool.Toolbox.event\_driven\_simulator.argmax  
 PyDSTool.Toolbox.event\_driven\_simulator.argmin  
 PyDSTool.Toolbox.event\_driven\_simulator.atleast\_1d  
 PyDSTool.Toolbox.event\_driven\_simulator.clip  
 PyDSTool.Toolbox.event\_driven\_simulator.complex  
 PyDSTool.Toolbox.event\_driven\_simulator.complex128  
 PyDSTool.Toolbox.event\_driven\_simulator.complex192  
 PyDSTool.Toolbox.event\_driven\_simulator.complex64  
 PyDSTool.Toolbox.event\_driven\_simulator.complex\_  
 PyDSTool.Toolbox.event\_driven\_simulator.complexfloating  
 PyDSTool.Toolbox.event\_driven\_simulator.deepcopy  
 PyDSTool.Toolbox.event\_driven\_simulator.double  
 PyDSTool.Toolbox.event\_driven\_simulator.exp  
 PyDSTool.Toolbox.event\_driven\_simulator.finfo  
 PyDSTool.Toolbox.event\_driven\_simulator.fit\_function  
 PyDSTool.Toolbox.event\_driven\_simulator.float  
 PyDSTool.Toolbox.event\_driven\_simulator.float32  
 PyDSTool.Toolbox.event\_driven\_simulator.float64  
 PyDSTool.Toolbox.event\_driven\_simulator.float96  
 PyDSTool.Toolbox.event\_driven\_simulator.float\_  
 PyDSTool.Toolbox.event\_driven\_simulator.floating  
 PyDSTool.Toolbox.event\_driven\_simulator.greater  
 PyDSTool.Toolbox.event\_driven\_simulator.idmap  
 PyDSTool.Toolbox.event\_driven\_simulator.int  
 PyDSTool.Toolbox.event\_driven\_simulator.int0  
 PyDSTool.Toolbox.event\_driven\_simulator.int16  
 PyDSTool.Toolbox.event\_driven\_simulator.int32  
 PyDSTool.Toolbox.event\_driven\_simulator.int64  
 PyDSTool.Toolbox.event\_driven\_simulator.int8  
 PyDSTool.Toolbox.event\_driven\_simulator.int\_  
 PyDSTool.Toolbox.event\_driven\_simulator.integer  
 PyDSTool.Toolbox.event\_driven\_simulator.intersect  

PyDSTool.Toolbox.event\_driven\_simulator.isfinite

PyDSTool.Toolbox.event\_driven\_simulator.less  
 PyDSTool.Toolbox.event\_driven\_simulator.less\_equal  
 PyDSTool.Toolbox.event\_driven\_simulator.log  
 PyDSTool.Toolbox.event\_driven\_simulator.logical\_or  
 PyDSTool.Toolbox.event\_driven\_simulator.mat  
 PyDSTool.Toolbox.event\_driven\_simulator.minpack  
 PyDSTool.Toolbox.event\_driven\_simulator.ndarray  
 PyDSTool.Toolbox.event\_driven\_simulator.norm  
 PyDSTool.Toolbox.event\_driven\_simulator.npy  

PyDSTool.Toolbox.event\_driven\_simulator.null\_predicate

PyDSTool.Toolbox.event\_driven\_simulator.null\_predicate\_class  
 PyDSTool.Toolbox.event\_driven\_simulator.ones  
 PyDSTool.Toolbox.event\_driven\_simulator.os  
 PyDSTool.Toolbox.event\_driven\_simulator.predicate\_op  
 PyDSTool.Toolbox.event\_driven\_simulator.putmask  
 PyDSTool.Toolbox.event\_driven\_simulator.searchsorted  
 PyDSTool.Toolbox.event\_driven\_simulator.shape  
 PyDSTool.Toolbox.event\_driven\_simulator.sign  
 PyDSTool.Toolbox.event\_driven\_simulator.sometrue  
 PyDSTool.Toolbox.event\_driven\_simulator.spy  
 PyDSTool.Toolbox.event\_driven\_simulator.sqrt  
 PyDSTool.Toolbox.event\_driven\_simulator.swapaxes  
 PyDSTool.Toolbox.event\_driven\_simulator.sys  
 PyDSTool.Toolbox.event\_driven\_simulator.take  

PyDSTool.Toolbox.event\_driven\_simulator.targetLangs

PyDSTool.Toolbox.event\_driven\_simulator.time  
 PyDSTool.Toolbox.event\_driven\_simulator.types  
 PyDSTool.Toolbox.event\_driven\_simulator.zeros  

PyDSTool.Toolbox.fracdim.ALLOW\_THREADS

PyDSTool.Toolbox.fracdim.Abs

PyDSTool.Toolbox.fracdim.Acos

PyDSTool.Toolbox.fracdim.Asin

PyDSTool.Toolbox.fracdim.Atan

PyDSTool.Toolbox.fracdim.Atan2

PyDSTool.Toolbox.fracdim.BUFSIZE

PyDSTool.Toolbox.fracdim.Betavariate

PyDSTool.Toolbox.fracdim.CLIP

PyDSTool.Toolbox.fracdim.Ceil

PyDSTool.Toolbox.fracdim.Choice

PyDSTool.Toolbox.fracdim.Cos

PyDSTool.Toolbox.fracdim.Cosh

PyDSTool.Toolbox.fracdim.Degrees

PyDSTool.Toolbox.fracdim.E

PyDSTool.Toolbox.fracdim.ERR\_CALL

PyDSTool.Toolbox.fracdim.ERR\_DEFAULT

PyDSTool.Toolbox.fracdim.ERR\_DEFAULT2

PyDSTool.Toolbox.fracdim.ERR\_IGNORE

PyDSTool.Toolbox.fracdim.ERR\_LOG

PyDSTool.Toolbox.fracdim.ERR\_PRINT

PyDSTool.Toolbox.fracdim.ERR\_RAISE

PyDSTool.Toolbox.fracdim.ERR\_WARN

PyDSTool.Toolbox.fracdim.Exp

PyDSTool.Toolbox.fracdim.Expovariate

PyDSTool.Toolbox.fracdim.FLOATING\_POINT\_SUPPORT

PyDSTool.Toolbox.fracdim.FPE\_DIVIDEBYZERO

PyDSTool.Toolbox.fracdim.FPE\_INVALID

PyDSTool.Toolbox.fracdim.FPE\_OVERFLOW

PyDSTool.Toolbox.fracdim.FPE\_UNDERFLOW

PyDSTool.Toolbox.fracdim.Fabs

PyDSTool.Toolbox.fracdim.False\_

PyDSTool.Toolbox.fracdim.Floor

PyDSTool.Toolbox.fracdim.Fmod

PyDSTool.Toolbox.fracdim.Frexp

PyDSTool.Toolbox.fracdim.Gammavariate

PyDSTool.Toolbox.fracdim.Gauss

PyDSTool.Toolbox.fracdim.Getrandbits

PyDSTool.Toolbox.fracdim.Getstate

PyDSTool.Toolbox.fracdim.Hypot

PyDSTool.Toolbox.fracdim.Infinity

PyDSTool.Toolbox.fracdim.Jumpahead

PyDSTool.Toolbox.fracdim.Ldexp

PyDSTool.Toolbox.fracdim.Log

PyDSTool.Toolbox.fracdim.Log10

PyDSTool.Toolbox.fracdim.Lognormvariate

PyDSTool.Toolbox.fracdim.MAXDIMS

PyDSTool.Toolbox.fracdim.Max

PyDSTool.Toolbox.fracdim.Min

PyDSTool.Toolbox.fracdim.Modf

PyDSTool.Toolbox.fracdim.NAN

PyDSTool.Toolbox.fracdim.NINF

PyDSTool.Toolbox.fracdim.NZERO

PyDSTool.Toolbox.fracdim.Normalvariate

PyDSTool.Toolbox.fracdim.PINF

PyDSTool.Toolbox.fracdim.PZERO

PyDSTool.Toolbox.fracdim.Paretovariate

PyDSTool.Toolbox.fracdim.Pi

PyDSTool.Toolbox.fracdim.Pow

PyDSTool.Toolbox.fracdim.RAISE

PyDSTool.Toolbox.fracdim.Radians

PyDSTool.Toolbox.fracdim.Randint

PyDSTool.Toolbox.fracdim.Random

PyDSTool.Toolbox.fracdim.Randrange

PyDSTool.Toolbox.fracdim.SHIFT\_DIVIDEBYZERO

PyDSTool.Toolbox.fracdim.SHIFT\_INVALID

PyDSTool.Toolbox.fracdim.SHIFT\_OVERFLOW

PyDSTool.Toolbox.fracdim.SHIFT\_UNDERFLOW

PyDSTool.Toolbox.fracdim.Sample

PyDSTool.Toolbox.fracdim.ScalarType

PyDSTool.Toolbox.fracdim.Seed

PyDSTool.Toolbox.fracdim.Setstate

PyDSTool.Toolbox.fracdim.Shuffle

PyDSTool.Toolbox.fracdim.Sin

PyDSTool.Toolbox.fracdim.Sinh

PyDSTool.Toolbox.fracdim.Sqrt

PyDSTool.Toolbox.fracdim.Sum

PyDSTool.Toolbox.fracdim.Systemrandom

PyDSTool.Toolbox.fracdim.Tan

PyDSTool.Toolbox.fracdim.Tanh

PyDSTool.Toolbox.fracdim.True\_

PyDSTool.Toolbox.fracdim.UFUNC\_BUFSIZE\_DEFAULT

PyDSTool.Toolbox.fracdim.UFUNC\_PYVALS\_NAME

PyDSTool.Toolbox.fracdim.Uniform

PyDSTool.Toolbox.fracdim.Vonmisesvariate

PyDSTool.Toolbox.fracdim.WRAP

PyDSTool.Toolbox.fracdim.Weibullvariate

PyDSTool.Toolbox.fracdim.Wichmannhill

PyDSTool.Toolbox.fracdim.absolute

PyDSTool.Toolbox.fracdim.add

PyDSTool.Toolbox.fracdim.bitwise\_and

PyDSTool.Toolbox.fracdim.bitwise\_not

PyDSTool.Toolbox.fracdim.bitwise\_or

PyDSTool.Toolbox.fracdim.bitwise\_xor

PyDSTool.Toolbox.fracdim.c\_

PyDSTool.Toolbox.fracdim.cast

PyDSTool.Toolbox.fracdim.conj

PyDSTool.Toolbox.fracdim.conjugate

PyDSTool.Toolbox.fracdim.copysign

PyDSTool.Toolbox.fracdim.deg2rad

PyDSTool.Toolbox.fracdim.divide

PyDSTool.Toolbox.fracdim.equal

PyDSTool.Toolbox.fracdim.exp2

PyDSTool.Toolbox.fracdim.expm1

PyDSTool.Toolbox.fracdim.floor\_divide

PyDSTool.Toolbox.fracdim.fmax

PyDSTool.Toolbox.fracdim.fmin

PyDSTool.Toolbox.fracdim.greater\_equal

PyDSTool.Toolbox.fracdim.index\_exp

PyDSTool.Toolbox.fracdim.inf

PyDSTool.Toolbox.fracdim.infty

PyDSTool.Toolbox.fracdim.invert

PyDSTool.Toolbox.fracdim.isinf

PyDSTool.Toolbox.fracdim.left\_shift

PyDSTool.Toolbox.fracdim.little\_endian

PyDSTool.Toolbox.fracdim.log1p

PyDSTool.Toolbox.fracdim.logaddexp

PyDSTool.Toolbox.fracdim.logaddexp2

PyDSTool.Toolbox.fracdim.logical\_and

PyDSTool.Toolbox.fracdim.logical\_not

PyDSTool.Toolbox.fracdim.logical\_xor

PyDSTool.Toolbox.fracdim.maximum

PyDSTool.Toolbox.fracdim.mgrid

PyDSTool.Toolbox.fracdim.minimum

PyDSTool.Toolbox.fracdim.multiply

PyDSTool.Toolbox.fracdim.n

PyDSTool.Toolbox.fracdim.nan

PyDSTool.Toolbox.fracdim.nbytes

PyDSTool.Toolbox.fracdim.negative

PyDSTool.Toolbox.fracdim.nextafter

PyDSTool.Toolbox.fracdim.not\_equal

PyDSTool.Toolbox.fracdim.ogrid

PyDSTool.Toolbox.fracdim.ones\_like

PyDSTool.Toolbox.fracdim.rad2deg

PyDSTool.Toolbox.fracdim.reciprocal

PyDSTool.Toolbox.fracdim.remainder

PyDSTool.Toolbox.fracdim.right\_shift

PyDSTool.Toolbox.fracdim.rint

PyDSTool.Toolbox.fracdim.s\_

PyDSTool.Toolbox.fracdim.sctypeDict

PyDSTool.Toolbox.fracdim.sctypeNA

PyDSTool.Toolbox.fracdim.sctypes

PyDSTool.Toolbox.fracdim.signbit

PyDSTool.Toolbox.fracdim.spacing

PyDSTool.Toolbox.fracdim.square

PyDSTool.Toolbox.fracdim.subtract

PyDSTool.Toolbox.fracdim.t

PyDSTool.Toolbox.fracdim.true\_divide

PyDSTool.Toolbox.fracdim.trunc

PyDSTool.Toolbox.fracdim.typeDict

PyDSTool.Toolbox.fracdim.typeNA

PyDSTool.Toolbox.fracdim.typecodes

PyDSTool.Toolbox.makeSloppyModel.ALLOW\_THREADS  
 PyDSTool.Toolbox.makeSloppyModel.Abs  
 PyDSTool.Toolbox.makeSloppyModel.Acos  
 PyDSTool.Toolbox.makeSloppyModel.Asin  
 PyDSTool.Toolbox.makeSloppyModel.Atan  
 PyDSTool.Toolbox.makeSloppyModel.Atan2  
 PyDSTool.Toolbox.makeSloppyModel.BUFSIZE  
 PyDSTool.Toolbox.makeSloppyModel.Betavariate  
 PyDSTool.Toolbox.makeSloppyModel.CLIP  
 PyDSTool.Toolbox.makeSloppyModel.Ceil  
 PyDSTool.Toolbox.makeSloppyModel.Choice  
 PyDSTool.Toolbox.makeSloppyModel.Cos  
 PyDSTool.Toolbox.makeSloppyModel.Cosh  
 PyDSTool.Toolbox.makeSloppyModel.Degrees  
 PyDSTool.Toolbox.makeSloppyModel.E  
 PyDSTool.Toolbox.makeSloppyModel.ERR\_CALL  
 PyDSTool.Toolbox.makeSloppyModel.ERR\_DEFAULT  
 PyDSTool.Toolbox.makeSloppyModel.ERR\_DEFAULT2  
 PyDSTool.Toolbox.makeSloppyModel.ERR\_IGNORE  
 PyDSTool.Toolbox.makeSloppyModel.ERR\_LOG  
 PyDSTool.Toolbox.makeSloppyModel.ERR\_PRINT  
 PyDSTool.Toolbox.makeSloppyModel.ERR\_RAISE  
 PyDSTool.Toolbox.makeSloppyModel.ERR\_WARN  
 PyDSTool.Toolbox.makeSloppyModel.Exp  
 PyDSTool.Toolbox.makeSloppyModel.Expovariate  
 PyDSTool.Toolbox.makeSloppyModel.FLOATING\_POINT\_SUPPORT  
 PyDSTool.Toolbox.makeSloppyModel.FPE\_DIVIDEBYZERO  
 PyDSTool.Toolbox.makeSloppyModel.FPE\_INVALID  
 PyDSTool.Toolbox.makeSloppyModel.FPE\_OVERFLOW  
 PyDSTool.Toolbox.makeSloppyModel.FPE\_UNDERFLOW  
 PyDSTool.Toolbox.makeSloppyModel.Fabs  
 PyDSTool.Toolbox.makeSloppyModel.False\_  
 PyDSTool.Toolbox.makeSloppyModel.Floor  
 PyDSTool.Toolbox.makeSloppyModel.Fmod  
 PyDSTool.Toolbox.makeSloppyModel.Frexp  
 PyDSTool.Toolbox.makeSloppyModel.Gammavariate  
 PyDSTool.Toolbox.makeSloppyModel.Gauss  
 PyDSTool.Toolbox.makeSloppyModel.Getrandbits  
 PyDSTool.Toolbox.makeSloppyModel.Getstate  
 PyDSTool.Toolbox.makeSloppyModel.Hypot  
 PyDSTool.Toolbox.makeSloppyModel.Infinity  
 PyDSTool.Toolbox.makeSloppyModel.Jumpahead  
 PyDSTool.Toolbox.makeSloppyModel.Ldexp  
 PyDSTool.Toolbox.makeSloppyModel.Log  
 PyDSTool.Toolbox.makeSloppyModel.Log10  
 PyDSTool.Toolbox.makeSloppyModel.Lognormvariate  
 PyDSTool.Toolbox.makeSloppyModel.MAXDIMS  
 PyDSTool.Toolbox.makeSloppyModel.Max  
 PyDSTool.Toolbox.makeSloppyModel.Min  
 PyDSTool.Toolbox.makeSloppyModel.Modf  
 PyDSTool.Toolbox.makeSloppyModel.NAN  
 PyDSTool.Toolbox.makeSloppyModel.NINF  
 PyDSTool.Toolbox.makeSloppyModel.NZERO  
 PyDSTool.Toolbox.makeSloppyModel.Normalvariate  
 PyDSTool.Toolbox.makeSloppyModel.PINF  
 PyDSTool.Toolbox.makeSloppyModel.PZERO  
 PyDSTool.Toolbox.makeSloppyModel.Paretovariate  
 PyDSTool.Toolbox.makeSloppyModel.Pi  
 PyDSTool.Toolbox.makeSloppyModel.Pow  
 PyDSTool.Toolbox.makeSloppyModel.RAISE  
 PyDSTool.Toolbox.makeSloppyModel.Radians  
 PyDSTool.Toolbox.makeSloppyModel.Randint  
 PyDSTool.Toolbox.makeSloppyModel.Random  
 PyDSTool.Toolbox.makeSloppyModel.Randrange  
 PyDSTool.Toolbox.makeSloppyModel.SHIFT\_DIVIDEBYZERO  
 PyDSTool.Toolbox.makeSloppyModel.SHIFT\_INVALID  
 PyDSTool.Toolbox.makeSloppyModel.SHIFT\_OVERFLOW  
 PyDSTool.Toolbox.makeSloppyModel.SHIFT\_UNDERFLOW  
 PyDSTool.Toolbox.makeSloppyModel.Sample  
 PyDSTool.Toolbox.makeSloppyModel.ScalarType  
 PyDSTool.Toolbox.makeSloppyModel.Seed  
 PyDSTool.Toolbox.makeSloppyModel.Setstate  
 PyDSTool.Toolbox.makeSloppyModel.Shuffle  
 PyDSTool.Toolbox.makeSloppyModel.Sin  
 PyDSTool.Toolbox.makeSloppyModel.Sinh  
 PyDSTool.Toolbox.makeSloppyModel.Sqrt  
 PyDSTool.Toolbox.makeSloppyModel.Sum  
 PyDSTool.Toolbox.makeSloppyModel.Systemrandom  
 PyDSTool.Toolbox.makeSloppyModel.Tan  
 PyDSTool.Toolbox.makeSloppyModel.Tanh  
 PyDSTool.Toolbox.makeSloppyModel.True\_  
 PyDSTool.Toolbox.makeSloppyModel.UFUNC\_BUFSIZE\_DEFAULT  
 PyDSTool.Toolbox.makeSloppyModel.UFUNC\_PYVALS\_NAME  
 PyDSTool.Toolbox.makeSloppyModel.Uniform  
 PyDSTool.Toolbox.makeSloppyModel.Vonmisesvariate  
 PyDSTool.Toolbox.makeSloppyModel.WRAP  
 PyDSTool.Toolbox.makeSloppyModel.Weibullvariate  
 PyDSTool.Toolbox.makeSloppyModel.Wichmannhill  
 PyDSTool.Toolbox.makeSloppyModel.absolute  
 PyDSTool.Toolbox.makeSloppyModel.add  
 PyDSTool.Toolbox.makeSloppyModel.allODEgens  
 PyDSTool.Toolbox.makeSloppyModel.bitwise\_and  
 PyDSTool.Toolbox.makeSloppyModel.bitwise\_not  
 PyDSTool.Toolbox.makeSloppyModel.bitwise\_or  
 PyDSTool.Toolbox.makeSloppyModel.bitwise\_xor  
 PyDSTool.Toolbox.makeSloppyModel.c\_  
 PyDSTool.Toolbox.makeSloppyModel.cast  
 PyDSTool.Toolbox.makeSloppyModel.conj  
 PyDSTool.Toolbox.makeSloppyModel.conjugate  
 PyDSTool.Toolbox.makeSloppyModel.copysign  
 PyDSTool.Toolbox.makeSloppyModel.deg2rad  
 PyDSTool.Toolbox.makeSloppyModel.divide  
 PyDSTool.Toolbox.makeSloppyModel.equal  
 PyDSTool.Toolbox.makeSloppyModel.exp2  
 PyDSTool.Toolbox.makeSloppyModel.expm1  
 PyDSTool.Toolbox.makeSloppyModel.floor\_divide  
 PyDSTool.Toolbox.makeSloppyModel.fmax  
 PyDSTool.Toolbox.makeSloppyModel.fmin  
 PyDSTool.Toolbox.makeSloppyModel.greater\_equal  
 PyDSTool.Toolbox.makeSloppyModel.index\_exp  
 PyDSTool.Toolbox.makeSloppyModel.inf  
 PyDSTool.Toolbox.makeSloppyModel.infty  
 PyDSTool.Toolbox.makeSloppyModel.invert  
 PyDSTool.Toolbox.makeSloppyModel.isinf  
 PyDSTool.Toolbox.makeSloppyModel.left\_shift  
 PyDSTool.Toolbox.makeSloppyModel.little\_endian  
 PyDSTool.Toolbox.makeSloppyModel.log1p  
 PyDSTool.Toolbox.makeSloppyModel.logaddexp  
 PyDSTool.Toolbox.makeSloppyModel.logaddexp2  
 PyDSTool.Toolbox.makeSloppyModel.logical\_and  
 PyDSTool.Toolbox.makeSloppyModel.logical\_not  
 PyDSTool.Toolbox.makeSloppyModel.logical\_xor  
 PyDSTool.Toolbox.makeSloppyModel.maximum  
 PyDSTool.Toolbox.makeSloppyModel.mgrid  
 PyDSTool.Toolbox.makeSloppyModel.minimum  
 PyDSTool.Toolbox.makeSloppyModel.multiply  
 PyDSTool.Toolbox.makeSloppyModel.n  
 PyDSTool.Toolbox.makeSloppyModel.nan  
 PyDSTool.Toolbox.makeSloppyModel.nbytes  
 PyDSTool.Toolbox.makeSloppyModel.negative  
 PyDSTool.Toolbox.makeSloppyModel.newaxis  
 PyDSTool.Toolbox.makeSloppyModel.nextafter  
 PyDSTool.Toolbox.makeSloppyModel.not\_equal  
 PyDSTool.Toolbox.makeSloppyModel.ogrid  
 PyDSTool.Toolbox.makeSloppyModel.ones\_like  
 PyDSTool.Toolbox.makeSloppyModel.r\_  
 PyDSTool.Toolbox.makeSloppyModel.rad2deg  
 PyDSTool.Toolbox.makeSloppyModel.reciprocal  
 PyDSTool.Toolbox.makeSloppyModel.remainder  
 PyDSTool.Toolbox.makeSloppyModel.right\_shift  
 PyDSTool.Toolbox.makeSloppyModel.rint  
 PyDSTool.Toolbox.makeSloppyModel.s\_  
 PyDSTool.Toolbox.makeSloppyModel.sctypeDict  
 PyDSTool.Toolbox.makeSloppyModel.sctypeNA  
 PyDSTool.Toolbox.makeSloppyModel.sctypes  
 PyDSTool.Toolbox.makeSloppyModel.signbit  
 PyDSTool.Toolbox.makeSloppyModel.spacing  
 PyDSTool.Toolbox.makeSloppyModel.square  
 PyDSTool.Toolbox.makeSloppyModel.subtract  
 PyDSTool.Toolbox.makeSloppyModel.t  
 PyDSTool.Toolbox.makeSloppyModel.true\_divide  
 PyDSTool.Toolbox.makeSloppyModel.trunc  
 PyDSTool.Toolbox.makeSloppyModel.typeDict  
 PyDSTool.Toolbox.makeSloppyModel.typeNA  
 PyDSTool.Toolbox.makeSloppyModel.typecodes  
 PyDSTool.Toolbox.mechmatlib.Id  
 PyDSTool.Toolbox.mechmatlib.ZeroPos  

PyDSTool.Toolbox.model\_primitives.alg\_spec

PyDSTool.Toolbox.model\_primitives.linear\_decay  

PyDSTool.Toolbox.model\_primitives.logistic\_spec

PyDSTool.Toolbox.model\_primitives.tanh\_spec

PyDSTool.Toolbox.model\_primitives.threshold  
 PyDSTool.Toolbox.neuralcomp.ALLOW\_THREADS  
 PyDSTool.Toolbox.neuralcomp.Abs  
 PyDSTool.Toolbox.neuralcomp.Acos  
 PyDSTool.Toolbox.neuralcomp.Asin  
 PyDSTool.Toolbox.neuralcomp.Atan  
 PyDSTool.Toolbox.neuralcomp.Atan2  
 PyDSTool.Toolbox.neuralcomp.BUFSIZE  
 PyDSTool.Toolbox.neuralcomp.Betavariate  
 PyDSTool.Toolbox.neuralcomp.CLIP  
 PyDSTool.Toolbox.neuralcomp.Ceil  
 PyDSTool.Toolbox.neuralcomp.Choice  
 PyDSTool.Toolbox.neuralcomp.Cos  
 PyDSTool.Toolbox.neuralcomp.Cosh  
 PyDSTool.Toolbox.neuralcomp.Degrees  
 PyDSTool.Toolbox.neuralcomp.E  
 PyDSTool.Toolbox.neuralcomp.ERR\_CALL  
 PyDSTool.Toolbox.neuralcomp.ERR\_DEFAULT  
 PyDSTool.Toolbox.neuralcomp.ERR\_DEFAULT2  
 PyDSTool.Toolbox.neuralcomp.ERR\_IGNORE  
 PyDSTool.Toolbox.neuralcomp.ERR\_LOG  
 PyDSTool.Toolbox.neuralcomp.ERR\_PRINT  
 PyDSTool.Toolbox.neuralcomp.ERR\_RAISE  
 PyDSTool.Toolbox.neuralcomp.ERR\_WARN  
 PyDSTool.Toolbox.neuralcomp.Exp  
 PyDSTool.Toolbox.neuralcomp.Expovariate  
 PyDSTool.Toolbox.neuralcomp.FLOATING\_POINT\_SUPPORT  
 PyDSTool.Toolbox.neuralcomp.FPE\_DIVIDEBYZERO  
 PyDSTool.Toolbox.neuralcomp.FPE\_INVALID  
 PyDSTool.Toolbox.neuralcomp.FPE\_OVERFLOW  
 PyDSTool.Toolbox.neuralcomp.FPE\_UNDERFLOW  
 PyDSTool.Toolbox.neuralcomp.Fabs  
 PyDSTool.Toolbox.neuralcomp.False\_  
 PyDSTool.Toolbox.neuralcomp.Floor  
 PyDSTool.Toolbox.neuralcomp.Fmod  
 PyDSTool.Toolbox.neuralcomp.Frexp  
 PyDSTool.Toolbox.neuralcomp.Gammavariate  
 PyDSTool.Toolbox.neuralcomp.Gauss  
 PyDSTool.Toolbox.neuralcomp.Getrandbits  
 PyDSTool.Toolbox.neuralcomp.Getstate  
 PyDSTool.Toolbox.neuralcomp.Hypot  
 PyDSTool.Toolbox.neuralcomp.Infinity  
 PyDSTool.Toolbox.neuralcomp.Jumpahead  
 PyDSTool.Toolbox.neuralcomp.Ldexp  
 PyDSTool.Toolbox.neuralcomp.Log  
 PyDSTool.Toolbox.neuralcomp.Log10  
 PyDSTool.Toolbox.neuralcomp.Lognormvariate  
 PyDSTool.Toolbox.neuralcomp.MAXDIMS  
 PyDSTool.Toolbox.neuralcomp.Max  
 PyDSTool.Toolbox.neuralcomp.Min  
 PyDSTool.Toolbox.neuralcomp.Modf  
 PyDSTool.Toolbox.neuralcomp.NAN  
 PyDSTool.Toolbox.neuralcomp.NINF  
 PyDSTool.Toolbox.neuralcomp.NZERO  
 PyDSTool.Toolbox.neuralcomp.Normalvariate  
 PyDSTool.Toolbox.neuralcomp.PINF  
 PyDSTool.Toolbox.neuralcomp.PZERO  
 PyDSTool.Toolbox.neuralcomp.Paretovariate  
 PyDSTool.Toolbox.neuralcomp.Pi  
 PyDSTool.Toolbox.neuralcomp.Pow  
 PyDSTool.Toolbox.neuralcomp.RAISE  
 PyDSTool.Toolbox.neuralcomp.Radians  
 PyDSTool.Toolbox.neuralcomp.Randint  
 PyDSTool.Toolbox.neuralcomp.Random  
 PyDSTool.Toolbox.neuralcomp.Randrange  
 PyDSTool.Toolbox.neuralcomp.SHIFT\_DIVIDEBYZERO  
 PyDSTool.Toolbox.neuralcomp.SHIFT\_INVALID  
 PyDSTool.Toolbox.neuralcomp.SHIFT\_OVERFLOW  
 PyDSTool.Toolbox.neuralcomp.SHIFT\_UNDERFLOW  
 PyDSTool.Toolbox.neuralcomp.Sample  
 PyDSTool.Toolbox.neuralcomp.ScalarType  
 PyDSTool.Toolbox.neuralcomp.Seed  
 PyDSTool.Toolbox.neuralcomp.Setstate  
 PyDSTool.Toolbox.neuralcomp.Shuffle  
 PyDSTool.Toolbox.neuralcomp.Sin  
 PyDSTool.Toolbox.neuralcomp.Sinh  
 PyDSTool.Toolbox.neuralcomp.Sqrt  
 PyDSTool.Toolbox.neuralcomp.Sum  
 PyDSTool.Toolbox.neuralcomp.Systemrandom  
 PyDSTool.Toolbox.neuralcomp.Tan  
 PyDSTool.Toolbox.neuralcomp.Tanh  
 PyDSTool.Toolbox.neuralcomp.True\_  
 PyDSTool.Toolbox.neuralcomp.UFUNC\_BUFSIZE\_DEFAULT  
 PyDSTool.Toolbox.neuralcomp.UFUNC\_PYVALS\_NAME  
 PyDSTool.Toolbox.neuralcomp.Uniform  
 PyDSTool.Toolbox.neuralcomp.V  
 PyDSTool.Toolbox.neuralcomp.Vonmisesvariate  
 PyDSTool.Toolbox.neuralcomp.WRAP  
 PyDSTool.Toolbox.neuralcomp.Weibullvariate  
 PyDSTool.Toolbox.neuralcomp.Wichmannhill  
 PyDSTool.Toolbox.neuralcomp.absolute  
 PyDSTool.Toolbox.neuralcomp.add  
 PyDSTool.Toolbox.neuralcomp.bitwise\_and  
 PyDSTool.Toolbox.neuralcomp.bitwise\_not  
 PyDSTool.Toolbox.neuralcomp.bitwise\_or  
 PyDSTool.Toolbox.neuralcomp.bitwise\_xor  
 PyDSTool.Toolbox.neuralcomp.c\_  
 PyDSTool.Toolbox.neuralcomp.cast  
 PyDSTool.Toolbox.neuralcomp.compatGens  
 PyDSTool.Toolbox.neuralcomp.conj  
 PyDSTool.Toolbox.neuralcomp.conjugate  
 PyDSTool.Toolbox.neuralcomp.copysign  
 PyDSTool.Toolbox.neuralcomp.deg2rad  
 PyDSTool.Toolbox.neuralcomp.divide  
 PyDSTool.Toolbox.neuralcomp.equal  
 PyDSTool.Toolbox.neuralcomp.exp2  
 PyDSTool.Toolbox.neuralcomp.expm1  
 PyDSTool.Toolbox.neuralcomp.floor\_divide  
 PyDSTool.Toolbox.neuralcomp.fmax  
 PyDSTool.Toolbox.neuralcomp.fmin  
 PyDSTool.Toolbox.neuralcomp.greater\_equal  
 PyDSTool.Toolbox.neuralcomp.index\_exp  
 PyDSTool.Toolbox.neuralcomp.inf  
 PyDSTool.Toolbox.neuralcomp.infty  
 PyDSTool.Toolbox.neuralcomp.invert  
 PyDSTool.Toolbox.neuralcomp.isinf  
 PyDSTool.Toolbox.neuralcomp.left\_shift  
 PyDSTool.Toolbox.neuralcomp.little\_endian  
 PyDSTool.Toolbox.neuralcomp.log1p  
 PyDSTool.Toolbox.neuralcomp.logaddexp  
 PyDSTool.Toolbox.neuralcomp.logaddexp2  
 PyDSTool.Toolbox.neuralcomp.logical\_and  
 PyDSTool.Toolbox.neuralcomp.logical\_not  
 PyDSTool.Toolbox.neuralcomp.logical\_xor  
 PyDSTool.Toolbox.neuralcomp.maximum  
 PyDSTool.Toolbox.neuralcomp.mgrid  
 PyDSTool.Toolbox.neuralcomp.minimum  
 PyDSTool.Toolbox.neuralcomp.multiply  
 PyDSTool.Toolbox.neuralcomp.n  
 PyDSTool.Toolbox.neuralcomp.nan  
 PyDSTool.Toolbox.neuralcomp.nbytes  
 PyDSTool.Toolbox.neuralcomp.negative  
 PyDSTool.Toolbox.neuralcomp.newaxis  
 PyDSTool.Toolbox.neuralcomp.nextafter  
 PyDSTool.Toolbox.neuralcomp.not\_equal  
 PyDSTool.Toolbox.neuralcomp.ogrid  
 PyDSTool.Toolbox.neuralcomp.ones\_like  
 PyDSTool.Toolbox.neuralcomp.r\_  
 PyDSTool.Toolbox.neuralcomp.rad2deg  
 PyDSTool.Toolbox.neuralcomp.reciprocal  
 PyDSTool.Toolbox.neuralcomp.remainder  
 PyDSTool.Toolbox.neuralcomp.right\_shift  
 PyDSTool.Toolbox.neuralcomp.rint  
 PyDSTool.Toolbox.neuralcomp.s\_  
 PyDSTool.Toolbox.neuralcomp.sctypeDict  
 PyDSTool.Toolbox.neuralcomp.sctypeNA  
 PyDSTool.Toolbox.neuralcomp.sctypes  
 PyDSTool.Toolbox.neuralcomp.signbit  
 PyDSTool.Toolbox.neuralcomp.spacing  
 PyDSTool.Toolbox.neuralcomp.square  
 PyDSTool.Toolbox.neuralcomp.subtract  
 PyDSTool.Toolbox.neuralcomp.t  
 PyDSTool.Toolbox.neuralcomp.true\_divide  
 PyDSTool.Toolbox.neuralcomp.trunc  
 PyDSTool.Toolbox.neuralcomp.typeDict  
 PyDSTool.Toolbox.neuralcomp.typeNA  
 PyDSTool.Toolbox.neuralcomp.typecodes  
 PyDSTool.Toolbox.neuralcomp.voltage  

PyDSTool.Toolbox.optimizers.criterion.criterion\_\_all\_\_

PyDSTool.Toolbox.optimizers.defaults.FAILED\_WITH\_UNIMPLEMENTED\_OR\_UNKNOWN\_REASON

PyDSTool.Toolbox.optimizers.defaults.FVAL\_IS\_ENOUGH

PyDSTool.Toolbox.optimizers.defaults.IS\_ALL\_VARS\_FIXED

PyDSTool.Toolbox.optimizers.defaults.IS\_LINE\_SEARCH\_FAILED

PyDSTool.Toolbox.optimizers.defaults.IS\_MAX\_CPU\_TIME\_REACHED

PyDSTool.Toolbox.optimizers.defaults.IS\_MAX\_FUN\_EVALS\_REACHED

PyDSTool.Toolbox.optimizers.defaults.IS\_MAX\_ITER\_REACHED

PyDSTool.Toolbox.optimizers.defaults.IS\_MAX\_TIME\_REACHED

PyDSTool.Toolbox.optimizers.defaults.IS\_NAN\_IN\_X

PyDSTool.Toolbox.optimizers.defaults.SMALL\_DELTA\_F

PyDSTool.Toolbox.optimizers.defaults.SMALL\_DELTA\_F\_F

PyDSTool.Toolbox.optimizers.defaults.SMALL\_DELTA\_X

PyDSTool.Toolbox.optimizers.defaults.SMALL\_DELTA\_X\_X

PyDSTool.Toolbox.optimizers.defaults.SMALL\_DF

PyDSTool.Toolbox.optimizers.defaults.SOLVED\_WITH\_UNIMPLEMENTED\_OR\_UNKNOWN\_REASON

PyDSTool.Toolbox.optimizers.defaults.errors  
 PyDSTool.Toolbox.optimizers.defaults.parameters  

PyDSTool.Toolbox.optimizers.helpers.helpers\_\_all\_\_

PyDSTool.Toolbox.optimizers.line\_search.line\_search\_\_all\_\_

PyDSTool.Toolbox.optimizers.optimizer.optimizer\_\_all\_\_

PyDSTool.Toolbox.optimizers.step.step\_\_all\_\_

PyDSTool.Toolbox.phaseplane.ALLOW\_THREADS

PyDSTool.Toolbox.phaseplane.API

PyDSTool.Toolbox.phaseplane.API\_class  

PyDSTool.Toolbox.phaseplane.Abs

PyDSTool.Toolbox.phaseplane.Acos

PyDSTool.Toolbox.phaseplane.Asin

PyDSTool.Toolbox.phaseplane.Atan

PyDSTool.Toolbox.phaseplane.Atan2

PyDSTool.Toolbox.phaseplane.BUFSIZE

PyDSTool.Toolbox.phaseplane.Betavariate

PyDSTool.Toolbox.phaseplane.CLIP

PyDSTool.Toolbox.phaseplane.Ceil

PyDSTool.Toolbox.phaseplane.Choice

PyDSTool.Toolbox.phaseplane.Continuous

PyDSTool.Toolbox.phaseplane.Cos

PyDSTool.Toolbox.phaseplane.Cosh

PyDSTool.Toolbox.phaseplane.DO\_DEC  
 PyDSTool.Toolbox.phaseplane.DO\_POW  

PyDSTool.Toolbox.phaseplane.Degrees

PyDSTool.Toolbox.phaseplane.Discrete

PyDSTool.Toolbox.phaseplane.DomainType  

PyDSTool.Toolbox.phaseplane.E

PyDSTool.Toolbox.phaseplane.ERR\_CALL

PyDSTool.Toolbox.phaseplane.ERR\_DEFAULT

PyDSTool.Toolbox.phaseplane.ERR\_DEFAULT2

PyDSTool.Toolbox.phaseplane.ERR\_IGNORE

PyDSTool.Toolbox.phaseplane.ERR\_LOG

PyDSTool.Toolbox.phaseplane.ERR\_PRINT

PyDSTool.Toolbox.phaseplane.ERR\_RAISE

PyDSTool.Toolbox.phaseplane.ERR\_WARN

PyDSTool.Toolbox.phaseplane.E\_COMPUTFAIL

PyDSTool.Toolbox.phaseplane.E\_NONUNIQUETERM

PyDSTool.Toolbox.phaseplane.Exp

PyDSTool.Toolbox.phaseplane.Expovariate

PyDSTool.Toolbox.phaseplane.FLOATING\_POINT\_SUPPORT

PyDSTool.Toolbox.phaseplane.FPE\_DIVIDEBYZERO

PyDSTool.Toolbox.phaseplane.FPE\_INVALID

PyDSTool.Toolbox.phaseplane.FPE\_OVERFLOW

PyDSTool.Toolbox.phaseplane.FPE\_UNDERFLOW

PyDSTool.Toolbox.phaseplane.Fabs

PyDSTool.Toolbox.phaseplane.False\_

PyDSTool.Toolbox.phaseplane.Floor

PyDSTool.Toolbox.phaseplane.Fmod

PyDSTool.Toolbox.phaseplane.Frexp

PyDSTool.Toolbox.phaseplane.Gammavariate

PyDSTool.Toolbox.phaseplane.Gauss

PyDSTool.Toolbox.phaseplane.Getrandbits

PyDSTool.Toolbox.phaseplane.Getstate

PyDSTool.Toolbox.phaseplane.HAVE\_PSYCO

PyDSTool.Toolbox.phaseplane.Hypot

PyDSTool.Toolbox.phaseplane.Inf

PyDSTool.Toolbox.phaseplane.Infinity

PyDSTool.Toolbox.phaseplane.Jumpahead

PyDSTool.Toolbox.phaseplane.LargestInt32

PyDSTool.Toolbox.phaseplane.Ldexp

PyDSTool.Toolbox.phaseplane.Log

PyDSTool.Toolbox.phaseplane.Log10

PyDSTool.Toolbox.phaseplane.Lognormvariate

PyDSTool.Toolbox.phaseplane.MAXDIMS

PyDSTool.Toolbox.phaseplane.MReg  
 PyDSTool.Toolbox.phaseplane.Macheps  

PyDSTool.Toolbox.phaseplane.Max

PyDSTool.Toolbox.phaseplane.Min

PyDSTool.Toolbox.phaseplane.Modf

PyDSTool.Toolbox.phaseplane.NAMESEP  

PyDSTool.Toolbox.phaseplane.NAN

PyDSTool.Toolbox.phaseplane.NINF

PyDSTool.Toolbox.phaseplane.NZERO

PyDSTool.Toolbox.phaseplane.NaN

PyDSTool.Toolbox.phaseplane.Normalvariate

PyDSTool.Toolbox.phaseplane.ONES  

PyDSTool.Toolbox.phaseplane.PINF

PyDSTool.Toolbox.phaseplane.POW\_STR  

PyDSTool.Toolbox.phaseplane.PZERO

PyDSTool.Toolbox.phaseplane.Paretovariate

PyDSTool.Toolbox.phaseplane.Pi

PyDSTool.Toolbox.phaseplane.Pow

PyDSTool.Toolbox.phaseplane.RAISE

PyDSTool.Toolbox.phaseplane.Radians

PyDSTool.Toolbox.phaseplane.Randint

PyDSTool.Toolbox.phaseplane.Random

PyDSTool.Toolbox.phaseplane.Randrange

PyDSTool.Toolbox.phaseplane.SHIFT\_DIVIDEBYZERO

PyDSTool.Toolbox.phaseplane.SHIFT\_INVALID

PyDSTool.Toolbox.phaseplane.SHIFT\_OVERFLOW

PyDSTool.Toolbox.phaseplane.SHIFT\_UNDERFLOW

PyDSTool.Toolbox.phaseplane.Sample

PyDSTool.Toolbox.phaseplane.ScalarType

PyDSTool.Toolbox.phaseplane.Seed

PyDSTool.Toolbox.phaseplane.Setstate

PyDSTool.Toolbox.phaseplane.Shuffle

PyDSTool.Toolbox.phaseplane.Sin

PyDSTool.Toolbox.phaseplane.Sinh

PyDSTool.Toolbox.phaseplane.Sqrt

PyDSTool.Toolbox.phaseplane.Sum

PyDSTool.Toolbox.phaseplane.Systemrandom

PyDSTool.Toolbox.phaseplane.TENS  

PyDSTool.Toolbox.phaseplane.Tan

PyDSTool.Toolbox.phaseplane.Tanh

PyDSTool.Toolbox.phaseplane.True\_

PyDSTool.Toolbox.phaseplane.UFUNC\_BUFSIZE\_DEFAULT

PyDSTool.Toolbox.phaseplane.UFUNC\_PYVALS\_NAME

PyDSTool.Toolbox.phaseplane.Uniform

PyDSTool.Toolbox.phaseplane.Vonmisesvariate

PyDSTool.Toolbox.phaseplane.WRAP

PyDSTool.Toolbox.phaseplane.W\_BISECTLIMIT

PyDSTool.Toolbox.phaseplane.W\_NONTERMEVENT

PyDSTool.Toolbox.phaseplane.W\_NONTERMSTATEBD

PyDSTool.Toolbox.phaseplane.W\_TERMEVENT

PyDSTool.Toolbox.phaseplane.W\_TERMSTATEBD

PyDSTool.Toolbox.phaseplane.W\_UNCERTVAL

PyDSTool.Toolbox.phaseplane.Weibullvariate

PyDSTool.Toolbox.phaseplane.Wichmannhill

PyDSTool.Toolbox.phaseplane.ZEROS  

PyDSTool.Toolbox.phaseplane.\_classes

PyDSTool.Toolbox.phaseplane.\_features

PyDSTool.Toolbox.phaseplane.\_functions

PyDSTool.Toolbox.phaseplane.\_num\_inf

PyDSTool.Toolbox.phaseplane.\_num\_types

PyDSTool.Toolbox.phaseplane.\_seq\_types

PyDSTool.Toolbox.phaseplane.absolute

PyDSTool.Toolbox.phaseplane.accrueCompTypes  
 PyDSTool.Toolbox.phaseplane.acosh  

PyDSTool.Toolbox.phaseplane.add

PyDSTool.Toolbox.phaseplane.allCompTypes  

PyDSTool.Toolbox.phaseplane.allmathnames

PyDSTool.Toolbox.phaseplane.allmathnames\_symbolic

PyDSTool.Toolbox.phaseplane.alphabet\_chars\_RE  

PyDSTool.Toolbox.phaseplane.arccos

PyDSTool.Toolbox.phaseplane.arccosh

PyDSTool.Toolbox.phaseplane.arcsin

PyDSTool.Toolbox.phaseplane.arcsinh

PyDSTool.Toolbox.phaseplane.arctan

PyDSTool.Toolbox.phaseplane.arctan2

PyDSTool.Toolbox.phaseplane.arctanh

PyDSTool.Toolbox.phaseplane.asinh  
 PyDSTool.Toolbox.phaseplane.ast2shortlist  
 PyDSTool.Toolbox.phaseplane.ast2string  
 PyDSTool.Toolbox.phaseplane.atanh  

PyDSTool.Toolbox.phaseplane.bitwise\_and

PyDSTool.Toolbox.phaseplane.bitwise\_not

PyDSTool.Toolbox.phaseplane.bitwise\_or

PyDSTool.Toolbox.phaseplane.bitwise\_xor

PyDSTool.Toolbox.phaseplane.builtinFnSigInfo  
 PyDSTool.Toolbox.phaseplane.builtin\_auxnames  
 PyDSTool.Toolbox.phaseplane.builtinlookup  
 PyDSTool.Toolbox.phaseplane.cPickle  

PyDSTool.Toolbox.phaseplane.c\_

PyDSTool.Toolbox.phaseplane.cast

PyDSTool.Toolbox.phaseplane.ceil

PyDSTool.Toolbox.phaseplane.collect\_numbers  
 PyDSTool.Toolbox.phaseplane.complex  
 PyDSTool.Toolbox.phaseplane.complex192  

PyDSTool.Toolbox.phaseplane.conj

PyDSTool.Toolbox.phaseplane.conjugate

PyDSTool.Toolbox.phaseplane.contained

PyDSTool.Toolbox.phaseplane.convert\_power\_reserved\_keywords  

PyDSTool.Toolbox.phaseplane.copysign

PyDSTool.Toolbox.phaseplane.cos

PyDSTool.Toolbox.phaseplane.cosh

PyDSTool.Toolbox.phaseplane.count\_sep  

PyDSTool.Toolbox.phaseplane.deg2rad

PyDSTool.Toolbox.phaseplane.degrees

PyDSTool.Toolbox.phaseplane.divide

PyDSTool.Toolbox.phaseplane.doadd  
 PyDSTool.Toolbox.phaseplane.dodiv  
 PyDSTool.Toolbox.phaseplane.dofun  
 PyDSTool.Toolbox.phaseplane.domul  
 PyDSTool.Toolbox.phaseplane.doneg  
 PyDSTool.Toolbox.phaseplane.dopower  
 PyDSTool.Toolbox.phaseplane.dosub  
 PyDSTool.Toolbox.phaseplane.dsInterface  

PyDSTool.Toolbox.phaseplane.e

PyDSTool.Toolbox.phaseplane.ensurebare  
 PyDSTool.Toolbox.phaseplane.ensuredecimalconst  
 PyDSTool.Toolbox.phaseplane.ensureints  
 PyDSTool.Toolbox.phaseplane.ensureparen  
 PyDSTool.Toolbox.phaseplane.ensureparen\_div  

PyDSTool.Toolbox.phaseplane.equal

PyDSTool.Toolbox.phaseplane.errmessages

PyDSTool.Toolbox.phaseplane.errorfields

PyDSTool.Toolbox.phaseplane.exp

PyDSTool.Toolbox.phaseplane.exp2

PyDSTool.Toolbox.phaseplane.expm1

PyDSTool.Toolbox.phaseplane.fabs

PyDSTool.Toolbox.phaseplane.feature\_leaf  
 PyDSTool.Toolbox.phaseplane.feature\_node  
 PyDSTool.Toolbox.phaseplane.feval\_map\_const  
 PyDSTool.Toolbox.phaseplane.feval\_map\_symb  
 PyDSTool.Toolbox.phaseplane.findEndBrace  
 PyDSTool.Toolbox.phaseplane.findNumTailPos  
 PyDSTool.Toolbox.phaseplane.fit\_function  
 PyDSTool.Toolbox.phaseplane.float96  

PyDSTool.Toolbox.phaseplane.floor

PyDSTool.Toolbox.phaseplane.floor\_divide

PyDSTool.Toolbox.phaseplane.fmax

PyDSTool.Toolbox.phaseplane.fmin

PyDSTool.Toolbox.phaseplane.fmod

PyDSTool.Toolbox.phaseplane.frexp

PyDSTool.Toolbox.phaseplane.funcnames  

PyDSTool.Toolbox.phaseplane.genDB

PyDSTool.Toolbox.phaseplane.getdim  
 PyDSTool.Toolbox.phaseplane.getlftrtD  

PyDSTool.Toolbox.phaseplane.greater

PyDSTool.Toolbox.phaseplane.greater\_equal

PyDSTool.Toolbox.phaseplane.hypot

PyDSTool.Toolbox.phaseplane.index\_exp

PyDSTool.Toolbox.phaseplane.inf

PyDSTool.Toolbox.phaseplane.infty

PyDSTool.Toolbox.phaseplane.inverseMathNameMap

PyDSTool.Toolbox.phaseplane.invert

PyDSTool.Toolbox.phaseplane.isHierarchicalName  
 PyDSTool.Toolbox.phaseplane.isIntegerToken  
 PyDSTool.Toolbox.phaseplane.isNameToken  
 PyDSTool.Toolbox.phaseplane.isNumericToken  
 PyDSTool.Toolbox.phaseplane.isToken  
 PyDSTool.Toolbox.phaseplane.isVectorClause  

PyDSTool.Toolbox.phaseplane.isfinite

PyDSTool.Toolbox.phaseplane.isinf

PyDSTool.Toolbox.phaseplane.isnan

PyDSTool.Toolbox.phaseplane.joinAsStrs  
 PyDSTool.Toolbox.phaseplane.joinStrs  

PyDSTool.Toolbox.phaseplane.ldexp

PyDSTool.Toolbox.phaseplane.left\_shift

PyDSTool.Toolbox.phaseplane.less

PyDSTool.Toolbox.phaseplane.less\_equal

PyDSTool.Toolbox.phaseplane.little\_endian

PyDSTool.Toolbox.phaseplane.local\_fndef  

PyDSTool.Toolbox.phaseplane.log

PyDSTool.Toolbox.phaseplane.log10

PyDSTool.Toolbox.phaseplane.log1p

PyDSTool.Toolbox.phaseplane.logaddexp

PyDSTool.Toolbox.phaseplane.logaddexp2

PyDSTool.Toolbox.phaseplane.logical\_and

PyDSTool.Toolbox.phaseplane.logical\_not

PyDSTool.Toolbox.phaseplane.logical\_or

PyDSTool.Toolbox.phaseplane.logical\_xor

PyDSTool.Toolbox.phaseplane.makeParList  
 PyDSTool.Toolbox.phaseplane.mapNames  
 PyDSTool.Toolbox.phaseplane.mapPowStr  

PyDSTool.Toolbox.phaseplane.mathNameMap

PyDSTool.Toolbox.phaseplane.math\_dir  
 PyDSTool.Toolbox.phaseplane.math\_globals  
 PyDSTool.Toolbox.phaseplane.mathlookup  

PyDSTool.Toolbox.phaseplane.maximum

PyDSTool.Toolbox.phaseplane.mgrid

PyDSTool.Toolbox.phaseplane.minimum

PyDSTool.Toolbox.phaseplane.mod

PyDSTool.Toolbox.phaseplane.modf

PyDSTool.Toolbox.phaseplane.modlookup  

PyDSTool.Toolbox.phaseplane.multiply

PyDSTool.Toolbox.phaseplane.n

PyDSTool.Toolbox.phaseplane.nameResolver

PyDSTool.Toolbox.phaseplane.name\_chars\_RE  

PyDSTool.Toolbox.phaseplane.nan

PyDSTool.Toolbox.phaseplane.nbytes

PyDSTool.Toolbox.phaseplane.negative

PyDSTool.Toolbox.phaseplane.newaxis

PyDSTool.Toolbox.phaseplane.nextafter

PyDSTool.Toolbox.phaseplane.not\_equal

PyDSTool.Toolbox.phaseplane.notcontained

PyDSTool.Toolbox.phaseplane.null\_predicate

PyDSTool.Toolbox.phaseplane.null\_predicate\_class  
 PyDSTool.Toolbox.phaseplane.num\_chars  

PyDSTool.Toolbox.phaseplane.ogrid

PyDSTool.Toolbox.phaseplane.ones\_like

PyDSTool.Toolbox.phaseplane.parseMatrixStrToDictStr  
 PyDSTool.Toolbox.phaseplane.parser  
 PyDSTool.Toolbox.phaseplane.parserObject  

PyDSTool.Toolbox.phaseplane.pi

PyDSTool.Toolbox.phaseplane.plotter  

PyDSTool.Toolbox.phaseplane.power

PyDSTool.Toolbox.phaseplane.predicate\_op  
 PyDSTool.Toolbox.phaseplane.processMultiDef  
 PyDSTool.Toolbox.phaseplane.process\_raw\_residual  
 PyDSTool.Toolbox.phaseplane.proper\_match  

PyDSTool.Toolbox.phaseplane.protected\_allnames

PyDSTool.Toolbox.phaseplane.protected\_auxnamesDB

PyDSTool.Toolbox.phaseplane.protected\_macronames  
 PyDSTool.Toolbox.phaseplane.protected\_mathnames  
 PyDSTool.Toolbox.phaseplane.protected\_randomnames  
 PyDSTool.Toolbox.phaseplane.protected\_scipynames  
 PyDSTool.Toolbox.phaseplane.protected\_specialfns  
 PyDSTool.Toolbox.phaseplane.qtypes  

PyDSTool.Toolbox.phaseplane.r\_

PyDSTool.Toolbox.phaseplane.rad2deg

PyDSTool.Toolbox.phaseplane.radians

PyDSTool.Toolbox.phaseplane.randomlookup  
 PyDSTool.Toolbox.phaseplane.re  
 PyDSTool.Toolbox.phaseplane.readArgs  

PyDSTool.Toolbox.phaseplane.reciprocal

PyDSTool.Toolbox.phaseplane.regObject  

PyDSTool.Toolbox.phaseplane.remainder

PyDSTool.Toolbox.phaseplane.remove\_indices\_from\_range  
 PyDSTool.Toolbox.phaseplane.replaceCallsWithDummies  
 PyDSTool.Toolbox.phaseplane.replaceSep  
 PyDSTool.Toolbox.phaseplane.replaceSepInv  
 PyDSTool.Toolbox.phaseplane.replaceSepList  
 PyDSTool.Toolbox.phaseplane.replaceSepListInv  
 PyDSTool.Toolbox.phaseplane.replaceSepQSpec  
 PyDSTool.Toolbox.phaseplane.replaceSepStr  

PyDSTool.Toolbox.phaseplane.rerr

PyDSTool.Toolbox.phaseplane.reserved\_keywords  
 PyDSTool.Toolbox.phaseplane.resolveSpecTypeCombos  

PyDSTool.Toolbox.phaseplane.right\_shift

PyDSTool.Toolbox.phaseplane.rint

PyDSTool.Toolbox.phaseplane.rout

PyDSTool.Toolbox.phaseplane.s\_

PyDSTool.Toolbox.phaseplane.sctypeDict

PyDSTool.Toolbox.phaseplane.sctypeNA

PyDSTool.Toolbox.phaseplane.sctypes

PyDSTool.Toolbox.phaseplane.sign

PyDSTool.Toolbox.phaseplane.signbit

PyDSTool.Toolbox.phaseplane.simplify  
 PyDSTool.Toolbox.phaseplane.simplify\_str  

PyDSTool.Toolbox.phaseplane.sin

PyDSTool.Toolbox.phaseplane.sinh

PyDSTool.Toolbox.phaseplane.spacing

PyDSTool.Toolbox.phaseplane.specTypes

PyDSTool.Toolbox.phaseplane.specialfns  
 PyDSTool.Toolbox.phaseplane.splitargs  
 PyDSTool.Toolbox.phaseplane.splitastLR  

PyDSTool.Toolbox.phaseplane.sqrt

PyDSTool.Toolbox.phaseplane.square

PyDSTool.Toolbox.phaseplane.strIfSeq  
 PyDSTool.Toolbox.phaseplane.string2ast  

PyDSTool.Toolbox.phaseplane.subtract

PyDSTool.Toolbox.phaseplane.sym2name  
 PyDSTool.Toolbox.phaseplane.symbol  
 PyDSTool.Toolbox.phaseplane.symbolMapClass  

PyDSTool.Toolbox.phaseplane.symbolMapDict

PyDSTool.Toolbox.phaseplane.syms  

PyDSTool.Toolbox.phaseplane.t

PyDSTool.Toolbox.phaseplane.tan

PyDSTool.Toolbox.phaseplane.tanh

PyDSTool.Toolbox.phaseplane.targetLangs

PyDSTool.Toolbox.phaseplane.temp\_macro\_names  
 PyDSTool.Toolbox.phaseplane.temp\_macro\_names\_inv  

PyDSTool.Toolbox.phaseplane.theGenSpecHelper

PyDSTool.Toolbox.phaseplane.toCircumflexSyntax  
 PyDSTool.Toolbox.phaseplane.toDoubleStarSyntax  
 PyDSTool.Toolbox.phaseplane.toPowSyntax  
 PyDSTool.Toolbox.phaseplane.token  
 PyDSTool.Toolbox.phaseplane.traceback  

PyDSTool.Toolbox.phaseplane.true\_divide

PyDSTool.Toolbox.phaseplane.trunc

PyDSTool.Toolbox.phaseplane.trysimple  
 PyDSTool.Toolbox.phaseplane.typeCounter  

PyDSTool.Toolbox.phaseplane.typeDict

PyDSTool.Toolbox.phaseplane.typeNA

PyDSTool.Toolbox.phaseplane.typecodes

PyDSTool.Toolbox.phaseplane.uncertain

PyDSTool.Toolbox.phaseplane.utils\_info  

PyDSTool.Toolbox.phaseplane.verstr\_parts

PyDSTool.Toolbox.phaseplane.warnfields

PyDSTool.Toolbox.phaseplane.warnmessages

PyDSTool.Toolbox.phaseplane.whoQ  
 PyDSTool.Toolbox.prep\_boxplot.symbol\_map  

PyDSTool.Toolbox.synthetic\_data.ALLOW\_THREADS

PyDSTool.Toolbox.synthetic\_data.API

PyDSTool.Toolbox.synthetic\_data.API\_class  

PyDSTool.Toolbox.synthetic\_data.Abs

PyDSTool.Toolbox.synthetic\_data.Acos

PyDSTool.Toolbox.synthetic\_data.Asin

PyDSTool.Toolbox.synthetic\_data.Atan

PyDSTool.Toolbox.synthetic\_data.Atan2

PyDSTool.Toolbox.synthetic\_data.BUFSIZE

PyDSTool.Toolbox.synthetic\_data.Betavariate

PyDSTool.Toolbox.synthetic\_data.CLIP

PyDSTool.Toolbox.synthetic\_data.Ceil

PyDSTool.Toolbox.synthetic\_data.Choice

PyDSTool.Toolbox.synthetic\_data.Continuous

PyDSTool.Toolbox.synthetic\_data.Cos

PyDSTool.Toolbox.synthetic\_data.Cosh

PyDSTool.Toolbox.synthetic\_data.DO\_DEC  
 PyDSTool.Toolbox.synthetic\_data.DO\_POW  

PyDSTool.Toolbox.synthetic\_data.Degrees

PyDSTool.Toolbox.synthetic\_data.Discrete

PyDSTool.Toolbox.synthetic\_data.DomainType  

PyDSTool.Toolbox.synthetic\_data.E

PyDSTool.Toolbox.synthetic\_data.ERR\_CALL

PyDSTool.Toolbox.synthetic\_data.ERR\_DEFAULT

PyDSTool.Toolbox.synthetic\_data.ERR\_DEFAULT2

PyDSTool.Toolbox.synthetic\_data.ERR\_IGNORE

PyDSTool.Toolbox.synthetic\_data.ERR\_LOG

PyDSTool.Toolbox.synthetic\_data.ERR\_PRINT

PyDSTool.Toolbox.synthetic\_data.ERR\_RAISE

PyDSTool.Toolbox.synthetic\_data.ERR\_WARN

PyDSTool.Toolbox.synthetic\_data.E\_COMPUTFAIL

PyDSTool.Toolbox.synthetic\_data.E\_NONUNIQUETERM

PyDSTool.Toolbox.synthetic\_data.Exp

PyDSTool.Toolbox.synthetic\_data.Expovariate

PyDSTool.Toolbox.synthetic\_data.FLOATING\_POINT\_SUPPORT

PyDSTool.Toolbox.synthetic\_data.FPE\_DIVIDEBYZERO

PyDSTool.Toolbox.synthetic\_data.FPE\_INVALID

PyDSTool.Toolbox.synthetic\_data.FPE\_OVERFLOW

PyDSTool.Toolbox.synthetic\_data.FPE\_UNDERFLOW

PyDSTool.Toolbox.synthetic\_data.Fabs

PyDSTool.Toolbox.synthetic\_data.False\_

PyDSTool.Toolbox.synthetic\_data.Floor

PyDSTool.Toolbox.synthetic\_data.Fmod

PyDSTool.Toolbox.synthetic\_data.Frexp

PyDSTool.Toolbox.synthetic\_data.Gammavariate

PyDSTool.Toolbox.synthetic\_data.Gauss

PyDSTool.Toolbox.synthetic\_data.Getrandbits

PyDSTool.Toolbox.synthetic\_data.Getstate

PyDSTool.Toolbox.synthetic\_data.HAVE\_PSYCO

PyDSTool.Toolbox.synthetic\_data.Hypot

PyDSTool.Toolbox.synthetic\_data.Inf

PyDSTool.Toolbox.synthetic\_data.Infinity

PyDSTool.Toolbox.synthetic\_data.Jumpahead

PyDSTool.Toolbox.synthetic\_data.LargestInt32

PyDSTool.Toolbox.synthetic\_data.Ldexp

PyDSTool.Toolbox.synthetic\_data.Log

PyDSTool.Toolbox.synthetic\_data.Log10

PyDSTool.Toolbox.synthetic\_data.Lognormvariate

PyDSTool.Toolbox.synthetic\_data.MAXDIMS

PyDSTool.Toolbox.synthetic\_data.MReg  
 PyDSTool.Toolbox.synthetic\_data.Macheps  

PyDSTool.Toolbox.synthetic\_data.Max

PyDSTool.Toolbox.synthetic\_data.Min

PyDSTool.Toolbox.synthetic\_data.Modf

PyDSTool.Toolbox.synthetic\_data.NAMESEP  

PyDSTool.Toolbox.synthetic\_data.NAN

PyDSTool.Toolbox.synthetic\_data.NINF

PyDSTool.Toolbox.synthetic\_data.NZERO

PyDSTool.Toolbox.synthetic\_data.NaN

PyDSTool.Toolbox.synthetic\_data.Normalvariate

PyDSTool.Toolbox.synthetic\_data.ONES  

PyDSTool.Toolbox.synthetic\_data.PINF

PyDSTool.Toolbox.synthetic\_data.POW\_STR  

PyDSTool.Toolbox.synthetic\_data.PZERO

PyDSTool.Toolbox.synthetic\_data.Paretovariate

PyDSTool.Toolbox.synthetic\_data.Pi

PyDSTool.Toolbox.synthetic\_data.Pow

PyDSTool.Toolbox.synthetic\_data.RAISE

PyDSTool.Toolbox.synthetic\_data.Radians

PyDSTool.Toolbox.synthetic\_data.Randint

PyDSTool.Toolbox.synthetic\_data.Random

PyDSTool.Toolbox.synthetic\_data.Randrange

PyDSTool.Toolbox.synthetic\_data.SHIFT\_DIVIDEBYZERO

PyDSTool.Toolbox.synthetic\_data.SHIFT\_INVALID

PyDSTool.Toolbox.synthetic\_data.SHIFT\_OVERFLOW

PyDSTool.Toolbox.synthetic\_data.SHIFT\_UNDERFLOW

PyDSTool.Toolbox.synthetic\_data.Sample

PyDSTool.Toolbox.synthetic\_data.ScalarType

PyDSTool.Toolbox.synthetic\_data.Seed

PyDSTool.Toolbox.synthetic\_data.Setstate

PyDSTool.Toolbox.synthetic\_data.Shuffle

PyDSTool.Toolbox.synthetic\_data.Sin

PyDSTool.Toolbox.synthetic\_data.Sinh

PyDSTool.Toolbox.synthetic\_data.Sqrt

PyDSTool.Toolbox.synthetic\_data.Sum

PyDSTool.Toolbox.synthetic\_data.Systemrandom

PyDSTool.Toolbox.synthetic\_data.TENS  

PyDSTool.Toolbox.synthetic\_data.Tan

PyDSTool.Toolbox.synthetic\_data.Tanh

PyDSTool.Toolbox.synthetic\_data.True\_

PyDSTool.Toolbox.synthetic\_data.UFUNC\_BUFSIZE\_DEFAULT

PyDSTool.Toolbox.synthetic\_data.UFUNC\_PYVALS\_NAME

PyDSTool.Toolbox.synthetic\_data.Uniform

PyDSTool.Toolbox.synthetic\_data.Vonmisesvariate

PyDSTool.Toolbox.synthetic\_data.WRAP

PyDSTool.Toolbox.synthetic\_data.W\_BISECTLIMIT

PyDSTool.Toolbox.synthetic\_data.W\_NONTERMEVENT

PyDSTool.Toolbox.synthetic\_data.W\_NONTERMSTATEBD

PyDSTool.Toolbox.synthetic\_data.W\_TERMEVENT

PyDSTool.Toolbox.synthetic\_data.W\_TERMSTATEBD

PyDSTool.Toolbox.synthetic\_data.W\_UNCERTVAL

PyDSTool.Toolbox.synthetic\_data.Weibullvariate

PyDSTool.Toolbox.synthetic\_data.Wichmannhill

PyDSTool.Toolbox.synthetic\_data.ZEROS  

PyDSTool.Toolbox.synthetic\_data.\_generators

PyDSTool.Toolbox.synthetic\_data.\_hypercuboid\_utils

PyDSTool.Toolbox.synthetic\_data.absolute

PyDSTool.Toolbox.synthetic\_data.accrueCompTypes  
 PyDSTool.Toolbox.synthetic\_data.acosh  

PyDSTool.Toolbox.synthetic\_data.add

PyDSTool.Toolbox.synthetic\_data.allCompTypes  

PyDSTool.Toolbox.synthetic\_data.allmathnames

PyDSTool.Toolbox.synthetic\_data.allmathnames\_symbolic

PyDSTool.Toolbox.synthetic\_data.alphabet\_chars\_RE  

PyDSTool.Toolbox.synthetic\_data.arccos

PyDSTool.Toolbox.synthetic\_data.arccosh

PyDSTool.Toolbox.synthetic\_data.arcsin

PyDSTool.Toolbox.synthetic\_data.arcsinh

PyDSTool.Toolbox.synthetic\_data.arctan

PyDSTool.Toolbox.synthetic\_data.arctan2

PyDSTool.Toolbox.synthetic\_data.arctanh

PyDSTool.Toolbox.synthetic\_data.asinh  
 PyDSTool.Toolbox.synthetic\_data.ast2shortlist  
 PyDSTool.Toolbox.synthetic\_data.ast2string  
 PyDSTool.Toolbox.synthetic\_data.atanh  

PyDSTool.Toolbox.synthetic\_data.bitwise\_and

PyDSTool.Toolbox.synthetic\_data.bitwise\_not

PyDSTool.Toolbox.synthetic\_data.bitwise\_or

PyDSTool.Toolbox.synthetic\_data.bitwise\_xor

PyDSTool.Toolbox.synthetic\_data.builtinFnSigInfo  
 PyDSTool.Toolbox.synthetic\_data.builtin\_auxnames  
 PyDSTool.Toolbox.synthetic\_data.builtinlookup  
 PyDSTool.Toolbox.synthetic\_data.cPickle  

PyDSTool.Toolbox.synthetic\_data.c\_

PyDSTool.Toolbox.synthetic\_data.cast

PyDSTool.Toolbox.synthetic\_data.ceil

PyDSTool.Toolbox.synthetic\_data.collect\_numbers  
 PyDSTool.Toolbox.synthetic\_data.complex  
 PyDSTool.Toolbox.synthetic\_data.complex192  

PyDSTool.Toolbox.synthetic\_data.conj

PyDSTool.Toolbox.synthetic\_data.conjugate

PyDSTool.Toolbox.synthetic\_data.contained

PyDSTool.Toolbox.synthetic\_data.convert\_power\_reserved\_keywords  

PyDSTool.Toolbox.synthetic\_data.copysign

PyDSTool.Toolbox.synthetic\_data.cos

PyDSTool.Toolbox.synthetic\_data.cosh

PyDSTool.Toolbox.synthetic\_data.count\_sep  

PyDSTool.Toolbox.synthetic\_data.deg2rad

PyDSTool.Toolbox.synthetic\_data.degrees

PyDSTool.Toolbox.synthetic\_data.divide

PyDSTool.Toolbox.synthetic\_data.doadd  
 PyDSTool.Toolbox.synthetic\_data.dodiv  
 PyDSTool.Toolbox.synthetic\_data.dofun  
 PyDSTool.Toolbox.synthetic\_data.domul  
 PyDSTool.Toolbox.synthetic\_data.doneg  
 PyDSTool.Toolbox.synthetic\_data.dopower  
 PyDSTool.Toolbox.synthetic\_data.dosub  
 PyDSTool.Toolbox.synthetic\_data.dsInterface  

PyDSTool.Toolbox.synthetic\_data.e

PyDSTool.Toolbox.synthetic\_data.ensurebare  
 PyDSTool.Toolbox.synthetic\_data.ensuredecimalconst  
 PyDSTool.Toolbox.synthetic\_data.ensureints  
 PyDSTool.Toolbox.synthetic\_data.ensureparen  
 PyDSTool.Toolbox.synthetic\_data.ensureparen\_div  

PyDSTool.Toolbox.synthetic\_data.equal

PyDSTool.Toolbox.synthetic\_data.errmessages

PyDSTool.Toolbox.synthetic\_data.errorfields

PyDSTool.Toolbox.synthetic\_data.exp

PyDSTool.Toolbox.synthetic\_data.exp2

PyDSTool.Toolbox.synthetic\_data.expm1

PyDSTool.Toolbox.synthetic\_data.fabs

PyDSTool.Toolbox.synthetic\_data.feature\_leaf  
 PyDSTool.Toolbox.synthetic\_data.feature\_node  
 PyDSTool.Toolbox.synthetic\_data.feval\_map\_const  
 PyDSTool.Toolbox.synthetic\_data.feval\_map\_symb  
 PyDSTool.Toolbox.synthetic\_data.findEndBrace  
 PyDSTool.Toolbox.synthetic\_data.findNumTailPos  
 PyDSTool.Toolbox.synthetic\_data.fit\_function  
 PyDSTool.Toolbox.synthetic\_data.float96  

PyDSTool.Toolbox.synthetic\_data.floor

PyDSTool.Toolbox.synthetic\_data.floor\_divide

PyDSTool.Toolbox.synthetic\_data.fmax

PyDSTool.Toolbox.synthetic\_data.fmin

PyDSTool.Toolbox.synthetic\_data.fmod

PyDSTool.Toolbox.synthetic\_data.frexp

PyDSTool.Toolbox.synthetic\_data.funcnames  

PyDSTool.Toolbox.synthetic\_data.genDB

PyDSTool.Toolbox.synthetic\_data.getdim  
 PyDSTool.Toolbox.synthetic\_data.getlftrtD  

PyDSTool.Toolbox.synthetic\_data.greater

PyDSTool.Toolbox.synthetic\_data.greater\_equal

PyDSTool.Toolbox.synthetic\_data.hypot

PyDSTool.Toolbox.synthetic\_data.index\_exp

PyDSTool.Toolbox.synthetic\_data.inf

PyDSTool.Toolbox.synthetic\_data.infty

PyDSTool.Toolbox.synthetic\_data.inverseMathNameMap

PyDSTool.Toolbox.synthetic\_data.invert

PyDSTool.Toolbox.synthetic\_data.isHierarchicalName  
 PyDSTool.Toolbox.synthetic\_data.isIntegerToken  
 PyDSTool.Toolbox.synthetic\_data.isNameToken  
 PyDSTool.Toolbox.synthetic\_data.isNumericToken  
 PyDSTool.Toolbox.synthetic\_data.isToken  
 PyDSTool.Toolbox.synthetic\_data.isVectorClause  

PyDSTool.Toolbox.synthetic\_data.isfinite

PyDSTool.Toolbox.synthetic\_data.isinf

PyDSTool.Toolbox.synthetic\_data.isnan

PyDSTool.Toolbox.synthetic\_data.joinAsStrs  
 PyDSTool.Toolbox.synthetic\_data.joinStrs  

PyDSTool.Toolbox.synthetic\_data.ldexp

PyDSTool.Toolbox.synthetic\_data.left\_shift

PyDSTool.Toolbox.synthetic\_data.less

PyDSTool.Toolbox.synthetic\_data.less\_equal

PyDSTool.Toolbox.synthetic\_data.little\_endian

PyDSTool.Toolbox.synthetic\_data.local\_fndef  

PyDSTool.Toolbox.synthetic\_data.log

PyDSTool.Toolbox.synthetic\_data.log10

PyDSTool.Toolbox.synthetic\_data.log1p

PyDSTool.Toolbox.synthetic\_data.logaddexp

PyDSTool.Toolbox.synthetic\_data.logaddexp2

PyDSTool.Toolbox.synthetic\_data.logical\_and

PyDSTool.Toolbox.synthetic\_data.logical\_not

PyDSTool.Toolbox.synthetic\_data.logical\_or

PyDSTool.Toolbox.synthetic\_data.logical\_xor

PyDSTool.Toolbox.synthetic\_data.makeParList  
 PyDSTool.Toolbox.synthetic\_data.mapNames  
 PyDSTool.Toolbox.synthetic\_data.mapPowStr  

PyDSTool.Toolbox.synthetic\_data.mathNameMap

PyDSTool.Toolbox.synthetic\_data.math\_dir  
 PyDSTool.Toolbox.synthetic\_data.math\_globals  
 PyDSTool.Toolbox.synthetic\_data.mathlookup  

PyDSTool.Toolbox.synthetic\_data.maximum

PyDSTool.Toolbox.synthetic\_data.mgrid

PyDSTool.Toolbox.synthetic\_data.minimum

PyDSTool.Toolbox.synthetic\_data.minpack  

PyDSTool.Toolbox.synthetic\_data.mod

PyDSTool.Toolbox.synthetic\_data.modf

PyDSTool.Toolbox.synthetic\_data.modlookup  

PyDSTool.Toolbox.synthetic\_data.multiply

PyDSTool.Toolbox.synthetic\_data.n

PyDSTool.Toolbox.synthetic\_data.nameResolver

PyDSTool.Toolbox.synthetic\_data.name\_chars\_RE  

PyDSTool.Toolbox.synthetic\_data.nan

PyDSTool.Toolbox.synthetic\_data.nbytes

PyDSTool.Toolbox.synthetic\_data.negative

PyDSTool.Toolbox.synthetic\_data.newaxis

PyDSTool.Toolbox.synthetic\_data.newton\_meth  

PyDSTool.Toolbox.synthetic\_data.nextafter

PyDSTool.Toolbox.synthetic\_data.not\_equal

PyDSTool.Toolbox.synthetic\_data.notcontained

PyDSTool.Toolbox.synthetic\_data.null\_predicate

PyDSTool.Toolbox.synthetic\_data.null\_predicate\_class  
 PyDSTool.Toolbox.synthetic\_data.num\_chars  

PyDSTool.Toolbox.synthetic\_data.ogrid

PyDSTool.Toolbox.synthetic\_data.ones\_like

PyDSTool.Toolbox.synthetic\_data.parseMatrixStrToDictStr  
 PyDSTool.Toolbox.synthetic\_data.parser  
 PyDSTool.Toolbox.synthetic\_data.parserObject  

PyDSTool.Toolbox.synthetic\_data.pi

PyDSTool.Toolbox.synthetic\_data.power

PyDSTool.Toolbox.synthetic\_data.predicate\_op  
 PyDSTool.Toolbox.synthetic\_data.processMultiDef  
 PyDSTool.Toolbox.synthetic\_data.process\_raw\_residual  
 PyDSTool.Toolbox.synthetic\_data.proper\_match  

PyDSTool.Toolbox.synthetic\_data.protected\_allnames

PyDSTool.Toolbox.synthetic\_data.protected\_auxnamesDB

PyDSTool.Toolbox.synthetic\_data.protected\_macronames  
 PyDSTool.Toolbox.synthetic\_data.protected\_mathnames  
 PyDSTool.Toolbox.synthetic\_data.protected\_randomnames  
 PyDSTool.Toolbox.synthetic\_data.protected\_scipynames  
 PyDSTool.Toolbox.synthetic\_data.protected\_specialfns  
 PyDSTool.Toolbox.synthetic\_data.qtypes  

PyDSTool.Toolbox.synthetic\_data.r\_

PyDSTool.Toolbox.synthetic\_data.rad2deg

PyDSTool.Toolbox.synthetic\_data.radians

PyDSTool.Toolbox.synthetic\_data.randomlookup  
 PyDSTool.Toolbox.synthetic\_data.re  
 PyDSTool.Toolbox.synthetic\_data.readArgs  

PyDSTool.Toolbox.synthetic\_data.reciprocal

PyDSTool.Toolbox.synthetic\_data.regObject  

PyDSTool.Toolbox.synthetic\_data.remainder

PyDSTool.Toolbox.synthetic\_data.remove\_indices\_from\_range  
 PyDSTool.Toolbox.synthetic\_data.replaceCallsWithDummies  
 PyDSTool.Toolbox.synthetic\_data.replaceSep  
 PyDSTool.Toolbox.synthetic\_data.replaceSepInv  
 PyDSTool.Toolbox.synthetic\_data.replaceSepList  
 PyDSTool.Toolbox.synthetic\_data.replaceSepListInv  
 PyDSTool.Toolbox.synthetic\_data.replaceSepQSpec  
 PyDSTool.Toolbox.synthetic\_data.replaceSepStr  

PyDSTool.Toolbox.synthetic\_data.rerr

PyDSTool.Toolbox.synthetic\_data.reserved\_keywords  
 PyDSTool.Toolbox.synthetic\_data.resolveSpecTypeCombos  

PyDSTool.Toolbox.synthetic\_data.right\_shift

PyDSTool.Toolbox.synthetic\_data.rint

PyDSTool.Toolbox.synthetic\_data.rout

PyDSTool.Toolbox.synthetic\_data.s\_

PyDSTool.Toolbox.synthetic\_data.sctypeDict

PyDSTool.Toolbox.synthetic\_data.sctypeNA

PyDSTool.Toolbox.synthetic\_data.sctypes

PyDSTool.Toolbox.synthetic\_data.sign

PyDSTool.Toolbox.synthetic\_data.signbit

PyDSTool.Toolbox.synthetic\_data.simplify  
 PyDSTool.Toolbox.synthetic\_data.simplify\_str  

PyDSTool.Toolbox.synthetic\_data.sin

PyDSTool.Toolbox.synthetic\_data.sinh

PyDSTool.Toolbox.synthetic\_data.spacing

PyDSTool.Toolbox.synthetic\_data.specTypes

PyDSTool.Toolbox.synthetic\_data.specialfns  
 PyDSTool.Toolbox.synthetic\_data.splitargs  
 PyDSTool.Toolbox.synthetic\_data.splitastLR  

PyDSTool.Toolbox.synthetic\_data.sqrt

PyDSTool.Toolbox.synthetic\_data.square

PyDSTool.Toolbox.synthetic\_data.strIfSeq  
 PyDSTool.Toolbox.synthetic\_data.string2ast  

PyDSTool.Toolbox.synthetic\_data.subtract

PyDSTool.Toolbox.synthetic\_data.sym2name  
 PyDSTool.Toolbox.synthetic\_data.symbol  
 PyDSTool.Toolbox.synthetic\_data.symbolMapClass  

PyDSTool.Toolbox.synthetic\_data.symbolMapDict

PyDSTool.Toolbox.synthetic\_data.syms  

PyDSTool.Toolbox.synthetic\_data.t

PyDSTool.Toolbox.synthetic\_data.tan

PyDSTool.Toolbox.synthetic\_data.tanh

PyDSTool.Toolbox.synthetic\_data.targetLangs

PyDSTool.Toolbox.synthetic\_data.temp\_macro\_names  
 PyDSTool.Toolbox.synthetic\_data.temp\_macro\_names\_inv  

PyDSTool.Toolbox.synthetic\_data.theGenSpecHelper

PyDSTool.Toolbox.synthetic\_data.toCircumflexSyntax  
 PyDSTool.Toolbox.synthetic\_data.toDoubleStarSyntax  
 PyDSTool.Toolbox.synthetic\_data.toPowSyntax  
 PyDSTool.Toolbox.synthetic\_data.token  
 PyDSTool.Toolbox.synthetic\_data.traceback  

PyDSTool.Toolbox.synthetic\_data.true\_divide

PyDSTool.Toolbox.synthetic\_data.trunc

PyDSTool.Toolbox.synthetic\_data.trysimple  
 PyDSTool.Toolbox.synthetic\_data.typeCounter  

PyDSTool.Toolbox.synthetic\_data.typeDict

PyDSTool.Toolbox.synthetic\_data.typeNA

PyDSTool.Toolbox.synthetic\_data.typecodes

PyDSTool.Toolbox.synthetic\_data.uncertain

PyDSTool.Toolbox.synthetic\_data.utils\_info  

PyDSTool.Toolbox.synthetic\_data.verstr\_parts

PyDSTool.Toolbox.synthetic\_data.warnfields

PyDSTool.Toolbox.synthetic\_data.warnmessages

PyDSTool.Toolbox.synthetic\_data.whoQ  

PyDSTool.Toolbox.syntheticdata.ALLOW\_THREADS

PyDSTool.Toolbox.syntheticdata.API

PyDSTool.Toolbox.syntheticdata.API\_class  

PyDSTool.Toolbox.syntheticdata.Abs

PyDSTool.Toolbox.syntheticdata.Acos

PyDSTool.Toolbox.syntheticdata.Asin

PyDSTool.Toolbox.syntheticdata.Atan

PyDSTool.Toolbox.syntheticdata.Atan2

PyDSTool.Toolbox.syntheticdata.BUFSIZE

PyDSTool.Toolbox.syntheticdata.Betavariate

PyDSTool.Toolbox.syntheticdata.CLIP

PyDSTool.Toolbox.syntheticdata.Ceil

PyDSTool.Toolbox.syntheticdata.Choice

PyDSTool.Toolbox.syntheticdata.Continuous

PyDSTool.Toolbox.syntheticdata.Cos

PyDSTool.Toolbox.syntheticdata.Cosh

PyDSTool.Toolbox.syntheticdata.DO\_DEC  
 PyDSTool.Toolbox.syntheticdata.DO\_POW  

PyDSTool.Toolbox.syntheticdata.Degrees

PyDSTool.Toolbox.syntheticdata.Discrete

PyDSTool.Toolbox.syntheticdata.DomainType  

PyDSTool.Toolbox.syntheticdata.E

PyDSTool.Toolbox.syntheticdata.ERR\_CALL

PyDSTool.Toolbox.syntheticdata.ERR\_DEFAULT

PyDSTool.Toolbox.syntheticdata.ERR\_DEFAULT2

PyDSTool.Toolbox.syntheticdata.ERR\_IGNORE

PyDSTool.Toolbox.syntheticdata.ERR\_LOG

PyDSTool.Toolbox.syntheticdata.ERR\_PRINT

PyDSTool.Toolbox.syntheticdata.ERR\_RAISE

PyDSTool.Toolbox.syntheticdata.ERR\_WARN

PyDSTool.Toolbox.syntheticdata.E\_COMPUTFAIL

PyDSTool.Toolbox.syntheticdata.E\_NONUNIQUETERM

PyDSTool.Toolbox.syntheticdata.Exp

PyDSTool.Toolbox.syntheticdata.Expovariate

PyDSTool.Toolbox.syntheticdata.FLOATING\_POINT\_SUPPORT

PyDSTool.Toolbox.syntheticdata.FPE\_DIVIDEBYZERO

PyDSTool.Toolbox.syntheticdata.FPE\_INVALID

PyDSTool.Toolbox.syntheticdata.FPE\_OVERFLOW

PyDSTool.Toolbox.syntheticdata.FPE\_UNDERFLOW

PyDSTool.Toolbox.syntheticdata.Fabs

PyDSTool.Toolbox.syntheticdata.False\_

PyDSTool.Toolbox.syntheticdata.Floor

PyDSTool.Toolbox.syntheticdata.Fmod

PyDSTool.Toolbox.syntheticdata.Frexp

PyDSTool.Toolbox.syntheticdata.Gammavariate

PyDSTool.Toolbox.syntheticdata.Gauss

PyDSTool.Toolbox.syntheticdata.Getrandbits

PyDSTool.Toolbox.syntheticdata.Getstate

PyDSTool.Toolbox.syntheticdata.HAVE\_PSYCO

PyDSTool.Toolbox.syntheticdata.Hypot

PyDSTool.Toolbox.syntheticdata.Inf

PyDSTool.Toolbox.syntheticdata.Infinity

PyDSTool.Toolbox.syntheticdata.Jumpahead

PyDSTool.Toolbox.syntheticdata.LargestInt32

PyDSTool.Toolbox.syntheticdata.Ldexp

PyDSTool.Toolbox.syntheticdata.Log

PyDSTool.Toolbox.syntheticdata.Log10

PyDSTool.Toolbox.syntheticdata.Lognormvariate

PyDSTool.Toolbox.syntheticdata.MAXDIMS

PyDSTool.Toolbox.syntheticdata.MReg  
 PyDSTool.Toolbox.syntheticdata.Macheps  

PyDSTool.Toolbox.syntheticdata.Max

PyDSTool.Toolbox.syntheticdata.Min

PyDSTool.Toolbox.syntheticdata.Modf

PyDSTool.Toolbox.syntheticdata.NAMESEP  

PyDSTool.Toolbox.syntheticdata.NAN

PyDSTool.Toolbox.syntheticdata.NINF

PyDSTool.Toolbox.syntheticdata.NZERO

PyDSTool.Toolbox.syntheticdata.NaN

PyDSTool.Toolbox.syntheticdata.Normalvariate

PyDSTool.Toolbox.syntheticdata.ONES  

PyDSTool.Toolbox.syntheticdata.PINF

PyDSTool.Toolbox.syntheticdata.POW\_STR  

PyDSTool.Toolbox.syntheticdata.PZERO

PyDSTool.Toolbox.syntheticdata.Paretovariate

PyDSTool.Toolbox.syntheticdata.Pi

PyDSTool.Toolbox.syntheticdata.Pow

PyDSTool.Toolbox.syntheticdata.RAISE

PyDSTool.Toolbox.syntheticdata.Radians

PyDSTool.Toolbox.syntheticdata.Randint

PyDSTool.Toolbox.syntheticdata.Random

PyDSTool.Toolbox.syntheticdata.Randrange

PyDSTool.Toolbox.syntheticdata.SHIFT\_DIVIDEBYZERO

PyDSTool.Toolbox.syntheticdata.SHIFT\_INVALID

PyDSTool.Toolbox.syntheticdata.SHIFT\_OVERFLOW

PyDSTool.Toolbox.syntheticdata.SHIFT\_UNDERFLOW

PyDSTool.Toolbox.syntheticdata.Sample

PyDSTool.Toolbox.syntheticdata.ScalarType

PyDSTool.Toolbox.syntheticdata.Seed

PyDSTool.Toolbox.syntheticdata.Setstate

PyDSTool.Toolbox.syntheticdata.Shuffle

PyDSTool.Toolbox.syntheticdata.Sin

PyDSTool.Toolbox.syntheticdata.Sinh

PyDSTool.Toolbox.syntheticdata.Sqrt

PyDSTool.Toolbox.syntheticdata.Sum

PyDSTool.Toolbox.syntheticdata.Systemrandom

PyDSTool.Toolbox.syntheticdata.TENS  

PyDSTool.Toolbox.syntheticdata.Tan

PyDSTool.Toolbox.syntheticdata.Tanh

PyDSTool.Toolbox.syntheticdata.True\_

PyDSTool.Toolbox.syntheticdata.UFUNC\_BUFSIZE\_DEFAULT

PyDSTool.Toolbox.syntheticdata.UFUNC\_PYVALS\_NAME

PyDSTool.Toolbox.syntheticdata.Uniform

PyDSTool.Toolbox.syntheticdata.Vonmisesvariate

PyDSTool.Toolbox.syntheticdata.WRAP

PyDSTool.Toolbox.syntheticdata.W\_BISECTLIMIT

PyDSTool.Toolbox.syntheticdata.W\_NONTERMEVENT

PyDSTool.Toolbox.syntheticdata.W\_NONTERMSTATEBD

PyDSTool.Toolbox.syntheticdata.W\_TERMEVENT

PyDSTool.Toolbox.syntheticdata.W\_TERMSTATEBD

PyDSTool.Toolbox.syntheticdata.W\_UNCERTVAL

PyDSTool.Toolbox.syntheticdata.Weibullvariate

PyDSTool.Toolbox.syntheticdata.Wichmannhill

PyDSTool.Toolbox.syntheticdata.ZEROS  

PyDSTool.Toolbox.syntheticdata.\_generators

PyDSTool.Toolbox.syntheticdata.\_hypercuboid\_utils

PyDSTool.Toolbox.syntheticdata.absolute

PyDSTool.Toolbox.syntheticdata.accrueCompTypes  
 PyDSTool.Toolbox.syntheticdata.acosh  

PyDSTool.Toolbox.syntheticdata.add

PyDSTool.Toolbox.syntheticdata.allCompTypes  

PyDSTool.Toolbox.syntheticdata.allmathnames

PyDSTool.Toolbox.syntheticdata.allmathnames\_symbolic

PyDSTool.Toolbox.syntheticdata.alphabet\_chars\_RE  

PyDSTool.Toolbox.syntheticdata.arccos

PyDSTool.Toolbox.syntheticdata.arccosh

PyDSTool.Toolbox.syntheticdata.arcsin

PyDSTool.Toolbox.syntheticdata.arcsinh

PyDSTool.Toolbox.syntheticdata.arctan

PyDSTool.Toolbox.syntheticdata.arctan2

PyDSTool.Toolbox.syntheticdata.arctanh

PyDSTool.Toolbox.syntheticdata.asinh  
 PyDSTool.Toolbox.syntheticdata.ast2shortlist  
 PyDSTool.Toolbox.syntheticdata.ast2string  
 PyDSTool.Toolbox.syntheticdata.atanh  

PyDSTool.Toolbox.syntheticdata.bitwise\_and

PyDSTool.Toolbox.syntheticdata.bitwise\_not

PyDSTool.Toolbox.syntheticdata.bitwise\_or

PyDSTool.Toolbox.syntheticdata.bitwise\_xor

PyDSTool.Toolbox.syntheticdata.builtinFnSigInfo  
 PyDSTool.Toolbox.syntheticdata.builtin\_auxnames  
 PyDSTool.Toolbox.syntheticdata.builtinlookup  
 PyDSTool.Toolbox.syntheticdata.cPickle  

PyDSTool.Toolbox.syntheticdata.c\_

PyDSTool.Toolbox.syntheticdata.cast

PyDSTool.Toolbox.syntheticdata.ceil

PyDSTool.Toolbox.syntheticdata.collect\_numbers  
 PyDSTool.Toolbox.syntheticdata.complex  
 PyDSTool.Toolbox.syntheticdata.complex192  

PyDSTool.Toolbox.syntheticdata.conj

PyDSTool.Toolbox.syntheticdata.conjugate

PyDSTool.Toolbox.syntheticdata.contained

PyDSTool.Toolbox.syntheticdata.convert\_power\_reserved\_keywords  

PyDSTool.Toolbox.syntheticdata.copysign

PyDSTool.Toolbox.syntheticdata.cos

PyDSTool.Toolbox.syntheticdata.cosh

PyDSTool.Toolbox.syntheticdata.count\_sep  

PyDSTool.Toolbox.syntheticdata.deg2rad

PyDSTool.Toolbox.syntheticdata.degrees

PyDSTool.Toolbox.syntheticdata.divide

PyDSTool.Toolbox.syntheticdata.doadd  
 PyDSTool.Toolbox.syntheticdata.dodiv  
 PyDSTool.Toolbox.syntheticdata.dofun  
 PyDSTool.Toolbox.syntheticdata.domul  
 PyDSTool.Toolbox.syntheticdata.doneg  
 PyDSTool.Toolbox.syntheticdata.dopower  
 PyDSTool.Toolbox.syntheticdata.dosub  
 PyDSTool.Toolbox.syntheticdata.dsInterface  

PyDSTool.Toolbox.syntheticdata.e

PyDSTool.Toolbox.syntheticdata.ensurebare  
 PyDSTool.Toolbox.syntheticdata.ensuredecimalconst  
 PyDSTool.Toolbox.syntheticdata.ensureints  
 PyDSTool.Toolbox.syntheticdata.ensureparen  
 PyDSTool.Toolbox.syntheticdata.ensureparen\_div  

PyDSTool.Toolbox.syntheticdata.equal

PyDSTool.Toolbox.syntheticdata.errmessages

PyDSTool.Toolbox.syntheticdata.errorfields

PyDSTool.Toolbox.syntheticdata.exp

PyDSTool.Toolbox.syntheticdata.exp2

PyDSTool.Toolbox.syntheticdata.expm1

PyDSTool.Toolbox.syntheticdata.fabs

PyDSTool.Toolbox.syntheticdata.feature\_leaf  
 PyDSTool.Toolbox.syntheticdata.feature\_node  
 PyDSTool.Toolbox.syntheticdata.feval\_map\_const  
 PyDSTool.Toolbox.syntheticdata.feval\_map\_symb  
 PyDSTool.Toolbox.syntheticdata.findEndBrace  
 PyDSTool.Toolbox.syntheticdata.findNumTailPos  
 PyDSTool.Toolbox.syntheticdata.fit\_function  
 PyDSTool.Toolbox.syntheticdata.float96  

PyDSTool.Toolbox.syntheticdata.floor

PyDSTool.Toolbox.syntheticdata.floor\_divide

PyDSTool.Toolbox.syntheticdata.fmax

PyDSTool.Toolbox.syntheticdata.fmin

PyDSTool.Toolbox.syntheticdata.fmod

PyDSTool.Toolbox.syntheticdata.frexp

PyDSTool.Toolbox.syntheticdata.funcnames  

PyDSTool.Toolbox.syntheticdata.genDB

PyDSTool.Toolbox.syntheticdata.getdim  
 PyDSTool.Toolbox.syntheticdata.getlftrtD  

PyDSTool.Toolbox.syntheticdata.greater

PyDSTool.Toolbox.syntheticdata.greater\_equal

PyDSTool.Toolbox.syntheticdata.hypot

PyDSTool.Toolbox.syntheticdata.index\_exp

PyDSTool.Toolbox.syntheticdata.inf

PyDSTool.Toolbox.syntheticdata.infty

PyDSTool.Toolbox.syntheticdata.inverseMathNameMap

PyDSTool.Toolbox.syntheticdata.invert

PyDSTool.Toolbox.syntheticdata.isHierarchicalName  
 PyDSTool.Toolbox.syntheticdata.isIntegerToken  
 PyDSTool.Toolbox.syntheticdata.isNameToken  
 PyDSTool.Toolbox.syntheticdata.isNumericToken  
 PyDSTool.Toolbox.syntheticdata.isToken  
 PyDSTool.Toolbox.syntheticdata.isVectorClause  

PyDSTool.Toolbox.syntheticdata.isfinite

PyDSTool.Toolbox.syntheticdata.isinf

PyDSTool.Toolbox.syntheticdata.isnan

PyDSTool.Toolbox.syntheticdata.joinAsStrs  
 PyDSTool.Toolbox.syntheticdata.joinStrs  

PyDSTool.Toolbox.syntheticdata.ldexp

PyDSTool.Toolbox.syntheticdata.left\_shift

PyDSTool.Toolbox.syntheticdata.less

PyDSTool.Toolbox.syntheticdata.less\_equal

PyDSTool.Toolbox.syntheticdata.little\_endian

PyDSTool.Toolbox.syntheticdata.local\_fndef  

PyDSTool.Toolbox.syntheticdata.log

PyDSTool.Toolbox.syntheticdata.log10

PyDSTool.Toolbox.syntheticdata.log1p

PyDSTool.Toolbox.syntheticdata.logaddexp

PyDSTool.Toolbox.syntheticdata.logaddexp2

PyDSTool.Toolbox.syntheticdata.logical\_and

PyDSTool.Toolbox.syntheticdata.logical\_not

PyDSTool.Toolbox.syntheticdata.logical\_or

PyDSTool.Toolbox.syntheticdata.logical\_xor

PyDSTool.Toolbox.syntheticdata.makeParList  
 PyDSTool.Toolbox.syntheticdata.mapNames  
 PyDSTool.Toolbox.syntheticdata.mapPowStr  

PyDSTool.Toolbox.syntheticdata.mathNameMap

PyDSTool.Toolbox.syntheticdata.math\_dir  
 PyDSTool.Toolbox.syntheticdata.math\_globals  
 PyDSTool.Toolbox.syntheticdata.mathlookup  

PyDSTool.Toolbox.syntheticdata.maximum

PyDSTool.Toolbox.syntheticdata.mgrid

PyDSTool.Toolbox.syntheticdata.minimum

PyDSTool.Toolbox.syntheticdata.minpack  

PyDSTool.Toolbox.syntheticdata.mod

PyDSTool.Toolbox.syntheticdata.modf

PyDSTool.Toolbox.syntheticdata.modlookup  

PyDSTool.Toolbox.syntheticdata.multiply

PyDSTool.Toolbox.syntheticdata.n

PyDSTool.Toolbox.syntheticdata.nameResolver

PyDSTool.Toolbox.syntheticdata.name\_chars\_RE  

PyDSTool.Toolbox.syntheticdata.nan

PyDSTool.Toolbox.syntheticdata.nbytes

PyDSTool.Toolbox.syntheticdata.negative

PyDSTool.Toolbox.syntheticdata.newaxis

PyDSTool.Toolbox.syntheticdata.newton\_meth  

PyDSTool.Toolbox.syntheticdata.nextafter

PyDSTool.Toolbox.syntheticdata.not\_equal

PyDSTool.Toolbox.syntheticdata.notcontained

PyDSTool.Toolbox.syntheticdata.null\_predicate

PyDSTool.Toolbox.syntheticdata.null\_predicate\_class  
 PyDSTool.Toolbox.syntheticdata.num\_chars  

PyDSTool.Toolbox.syntheticdata.ogrid

PyDSTool.Toolbox.syntheticdata.ones\_like

PyDSTool.Toolbox.syntheticdata.parseMatrixStrToDictStr  
 PyDSTool.Toolbox.syntheticdata.parser  
 PyDSTool.Toolbox.syntheticdata.parserObject  

PyDSTool.Toolbox.syntheticdata.pi

PyDSTool.Toolbox.syntheticdata.power

PyDSTool.Toolbox.syntheticdata.predicate\_op  
 PyDSTool.Toolbox.syntheticdata.processMultiDef  
 PyDSTool.Toolbox.syntheticdata.process\_raw\_residual  
 PyDSTool.Toolbox.syntheticdata.proper\_match  

PyDSTool.Toolbox.syntheticdata.protected\_allnames

PyDSTool.Toolbox.syntheticdata.protected\_auxnamesDB

PyDSTool.Toolbox.syntheticdata.protected\_macronames  
 PyDSTool.Toolbox.syntheticdata.protected\_mathnames  
 PyDSTool.Toolbox.syntheticdata.protected\_randomnames  
 PyDSTool.Toolbox.syntheticdata.protected\_scipynames  
 PyDSTool.Toolbox.syntheticdata.protected\_specialfns  
 PyDSTool.Toolbox.syntheticdata.qtypes  

PyDSTool.Toolbox.syntheticdata.r\_

PyDSTool.Toolbox.syntheticdata.rad2deg

PyDSTool.Toolbox.syntheticdata.radians

PyDSTool.Toolbox.syntheticdata.randomlookup  
 PyDSTool.Toolbox.syntheticdata.re  
 PyDSTool.Toolbox.syntheticdata.readArgs  

PyDSTool.Toolbox.syntheticdata.reciprocal

PyDSTool.Toolbox.syntheticdata.regObject  

PyDSTool.Toolbox.syntheticdata.remainder

PyDSTool.Toolbox.syntheticdata.remove\_indices\_from\_range  
 PyDSTool.Toolbox.syntheticdata.replaceCallsWithDummies  
 PyDSTool.Toolbox.syntheticdata.replaceSep  
 PyDSTool.Toolbox.syntheticdata.replaceSepInv  
 PyDSTool.Toolbox.syntheticdata.replaceSepList  
 PyDSTool.Toolbox.syntheticdata.replaceSepListInv  
 PyDSTool.Toolbox.syntheticdata.replaceSepQSpec  
 PyDSTool.Toolbox.syntheticdata.replaceSepStr  

PyDSTool.Toolbox.syntheticdata.rerr

PyDSTool.Toolbox.syntheticdata.reserved\_keywords  
 PyDSTool.Toolbox.syntheticdata.resolveSpecTypeCombos  

PyDSTool.Toolbox.syntheticdata.right\_shift

PyDSTool.Toolbox.syntheticdata.rint

PyDSTool.Toolbox.syntheticdata.rout

PyDSTool.Toolbox.syntheticdata.s\_

PyDSTool.Toolbox.syntheticdata.sctypeDict

PyDSTool.Toolbox.syntheticdata.sctypeNA

PyDSTool.Toolbox.syntheticdata.sctypes

PyDSTool.Toolbox.syntheticdata.sign

PyDSTool.Toolbox.syntheticdata.signbit

PyDSTool.Toolbox.syntheticdata.simplify  
 PyDSTool.Toolbox.syntheticdata.simplify\_str  

PyDSTool.Toolbox.syntheticdata.sin

PyDSTool.Toolbox.syntheticdata.sinh

PyDSTool.Toolbox.syntheticdata.spacing

PyDSTool.Toolbox.syntheticdata.specTypes

PyDSTool.Toolbox.syntheticdata.specialfns  
 PyDSTool.Toolbox.syntheticdata.splitargs  
 PyDSTool.Toolbox.syntheticdata.splitastLR  

PyDSTool.Toolbox.syntheticdata.sqrt

PyDSTool.Toolbox.syntheticdata.square

PyDSTool.Toolbox.syntheticdata.strIfSeq  
 PyDSTool.Toolbox.syntheticdata.string2ast  

PyDSTool.Toolbox.syntheticdata.subtract

PyDSTool.Toolbox.syntheticdata.sym2name  
 PyDSTool.Toolbox.syntheticdata.symbol  
 PyDSTool.Toolbox.syntheticdata.symbolMapClass  

PyDSTool.Toolbox.syntheticdata.symbolMapDict

PyDSTool.Toolbox.syntheticdata.syms  

PyDSTool.Toolbox.syntheticdata.t

PyDSTool.Toolbox.syntheticdata.tan

PyDSTool.Toolbox.syntheticdata.tanh

PyDSTool.Toolbox.syntheticdata.targetLangs

PyDSTool.Toolbox.syntheticdata.temp\_macro\_names  
 PyDSTool.Toolbox.syntheticdata.temp\_macro\_names\_inv  

PyDSTool.Toolbox.syntheticdata.theGenSpecHelper

PyDSTool.Toolbox.syntheticdata.toCircumflexSyntax  
 PyDSTool.Toolbox.syntheticdata.toDoubleStarSyntax  
 PyDSTool.Toolbox.syntheticdata.toPowSyntax  
 PyDSTool.Toolbox.syntheticdata.token  
 PyDSTool.Toolbox.syntheticdata.traceback  

PyDSTool.Toolbox.syntheticdata.true\_divide

PyDSTool.Toolbox.syntheticdata.trunc

PyDSTool.Toolbox.syntheticdata.trysimple  
 PyDSTool.Toolbox.syntheticdata.typeCounter  

PyDSTool.Toolbox.syntheticdata.typeDict

PyDSTool.Toolbox.syntheticdata.typeNA

PyDSTool.Toolbox.syntheticdata.typecodes

PyDSTool.Toolbox.syntheticdata.uncertain

PyDSTool.Toolbox.syntheticdata.utils\_info  

PyDSTool.Toolbox.syntheticdata.verstr\_parts

PyDSTool.Toolbox.syntheticdata.warnfields

PyDSTool.Toolbox.syntheticdata.warnmessages

PyDSTool.Toolbox.syntheticdata.whoQ  

PyDSTool.Trajectory'.API

PyDSTool.Trajectory'.Continuous

PyDSTool.Trajectory'.Discrete

PyDSTool.Trajectory'.Inf

PyDSTool.Trajectory'.LargestInt32

PyDSTool.Trajectory'.NAMESEP

PyDSTool.Trajectory'.ONES

PyDSTool.Trajectory'.ZEROS

PyDSTool.Trajectory'.\_1DimplicitSolveMethods

PyDSTool.Trajectory'.\_all\_complex

PyDSTool.Trajectory'.\_all\_float

PyDSTool.Trajectory'.\_all\_int

PyDSTool.Trajectory'.\_all\_numpy\_complex

PyDSTool.Trajectory'.\_all\_numpy\_float

PyDSTool.Trajectory'.\_all\_numpy\_int

PyDSTool.Trajectory'.\_complex\_types

PyDSTool.Trajectory'.\_float\_types

PyDSTool.Trajectory'.\_implicitSolveMethods

PyDSTool.Trajectory'.\_indentstr

PyDSTool.Trajectory'.\_int\_types

PyDSTool.Trajectory'.\_num\_equivtype

PyDSTool.Trajectory'.\_num\_maxmin

PyDSTool.Trajectory'.\_num\_name2equivtypes

PyDSTool.Trajectory'.\_num\_name2type

PyDSTool.Trajectory'.\_num\_type2name

PyDSTool.Trajectory'.\_num\_types

PyDSTool.Trajectory'.\_pytypefromtype

PyDSTool.Trajectory'.\_real\_types

PyDSTool.Trajectory'.\_seq\_types

PyDSTool.Trajectory'.builtinFnSigInfo

PyDSTool.Trajectory'.builtin\_auxnames

PyDSTool.Trajectory'.contained

PyDSTool.Trajectory'.name\_chars\_RE

PyDSTool.Trajectory'.notcontained

PyDSTool.Trajectory'.null\_predicate

PyDSTool.Trajectory'.num\_chars

PyDSTool.Trajectory'.protected\_allnames

PyDSTool.Trajectory'.protected\_auxnamesDB

PyDSTool.Trajectory'.protected\_macronames

PyDSTool.Trajectory'.protected\_mathnames

PyDSTool.Trajectory'.protected\_randomnames

PyDSTool.Trajectory'.protected\_scipynames

PyDSTool.Trajectory'.protected\_specialfns

PyDSTool.Trajectory'.syms

PyDSTool.Trajectory'.targetLangs

PyDSTool.Trajectory'.uncertain

PyDSTool.True\_  
 PyDSTool.UFUNC\_BUFSIZE\_DEFAULT  
 PyDSTool.UFUNC\_PYVALS\_NAME  
 PyDSTool.Uniform  

PyDSTool.Variable'.API

PyDSTool.Variable'.Continuous

PyDSTool.Variable'.Discrete

PyDSTool.Variable'.Inf

PyDSTool.Variable'.LargestInt32

PyDSTool.Variable'.NaN

PyDSTool.Variable'.\_1DimplicitSolveMethods

PyDSTool.Variable'.\_all\_complex

PyDSTool.Variable'.\_all\_float

PyDSTool.Variable'.\_all\_int

PyDSTool.Variable'.\_all\_numpy\_complex

PyDSTool.Variable'.\_all\_numpy\_float

PyDSTool.Variable'.\_all\_numpy\_int

PyDSTool.Variable'.\_complex\_types

PyDSTool.Variable'.\_float\_types

PyDSTool.Variable'.\_implicitSolveMethods

PyDSTool.Variable'.\_int\_types

PyDSTool.Variable'.\_num\_equivtype

PyDSTool.Variable'.\_num\_maxmin

PyDSTool.Variable'.\_num\_name2equivtypes

PyDSTool.Variable'.\_num\_name2type

PyDSTool.Variable'.\_num\_type2name

PyDSTool.Variable'.\_num\_types

PyDSTool.Variable'.\_pytypefromtype

PyDSTool.Variable'.\_real\_types

PyDSTool.Variable'.\_seq\_types

PyDSTool.Variable'.contained

PyDSTool.Variable'.isfinite

PyDSTool.Variable'.notcontained

PyDSTool.Variable'.null\_predicate

PyDSTool.Variable'.targetLangs

PyDSTool.Variable'.uncertain

PyDSTool.Vonmisesvariate  
 PyDSTool.WRAP  
 PyDSTool.Weibullvariate  
 PyDSTool.Wichmannhill  

PyDSTool.\_1DimplicitSolveMethods

PyDSTool.\_\_LICENSE\_\_  
 PyDSTool.\_\_revision\_\_  

PyDSTool.\_\_session\_ext

PyDSTool.\_\_symbolic\_ext

PyDSTool.\_implicitSolveMethods

PyDSTool.\_pyDSToolTypes

PyDSTool.absolute  
 PyDSTool.add  
 PyDSTool.bitwise\_and  
 PyDSTool.bitwise\_not  
 PyDSTool.bitwise\_or  
 PyDSTool.bitwise\_xor  
 PyDSTool.c\_  
 PyDSTool.cast  
 PyDSTool.common.API  
 PyDSTool.common.Continuous  
 PyDSTool.common.Discrete  

PyDSTool.common.Inf

PyDSTool.common.LargestInt32  

PyDSTool.common.Macheps

PyDSTool.common.NaN

PyDSTool.common.\_all\_complex  
 PyDSTool.common.\_all\_float  
 PyDSTool.common.\_all\_int  
 PyDSTool.common.\_all\_numpy\_complex  
 PyDSTool.common.\_all\_numpy\_float  
 PyDSTool.common.\_all\_numpy\_int  

PyDSTool.common.\_classes

PyDSTool.common.\_complex\_types  

PyDSTool.common.\_constants

PyDSTool.common.\_float\_types  

PyDSTool.common.\_functions

PyDSTool.common.\_int\_types  

PyDSTool.common.\_mappings

PyDSTool.common.\_num\_equivtype  
 PyDSTool.common.\_num\_maxmin  
 PyDSTool.common.\_num\_name2equivtypes  
 PyDSTool.common.\_num\_name2type  
 PyDSTool.common.\_num\_type2name  
 PyDSTool.common.\_num\_types  
 PyDSTool.common.\_pytypefromtype  
 PyDSTool.common.\_real\_types  
 PyDSTool.common.\_seq\_types  

PyDSTool.common.\_typefrompytype

PyDSTool.common.\_verify\_type\_names

PyDSTool.common.complex192  

PyDSTool.common.exp

PyDSTool.common.float96  

PyDSTool.common.greater

PyDSTool.common.isfinite

PyDSTool.common.less

PyDSTool.common.less\_equal

PyDSTool.common.log

PyDSTool.common.logical\_or

PyDSTool.common.null\_predicate  

PyDSTool.common.sign

PyDSTool.common.targetLangs  
 PyDSTool.conf.copyright  
 PyDSTool.conf.exclude\_patterns  
 PyDSTool.conf.extensions  
 PyDSTool.conf.html\_static\_path  
 PyDSTool.conf.html\_theme  
 PyDSTool.conf.htmlhelp\_basename  
 PyDSTool.conf.latex\_documents  
 PyDSTool.conf.man\_pages  
 PyDSTool.conf.master\_doc  
 PyDSTool.conf.project  
 PyDSTool.conf.pygments\_style  
 PyDSTool.conf.release  
 PyDSTool.conf.source\_suffix  
 PyDSTool.conf.templates\_path  
 PyDSTool.conf.version  
 PyDSTool.conj  
 PyDSTool.conjugate  
 PyDSTool.copysign  
 PyDSTool.deg2rad  
 PyDSTool.divide  
 PyDSTool.equal  
 PyDSTool.exp2  
 PyDSTool.expm1  
 PyDSTool.fixedpickle.APPEND  
 PyDSTool.fixedpickle.APPENDS  
 PyDSTool.fixedpickle.BINFLOAT  
 PyDSTool.fixedpickle.BINGET  
 PyDSTool.fixedpickle.BININT  
 PyDSTool.fixedpickle.BININT1  
 PyDSTool.fixedpickle.BININT2  
 PyDSTool.fixedpickle.BINPERSID  
 PyDSTool.fixedpickle.BINPUT  
 PyDSTool.fixedpickle.BINSTRING  
 PyDSTool.fixedpickle.BINUNICODE  
 PyDSTool.fixedpickle.BUILD  
 PyDSTool.fixedpickle.DICT  
 PyDSTool.fixedpickle.DUP  
 PyDSTool.fixedpickle.EMPTY\_DICT  
 PyDSTool.fixedpickle.EMPTY\_LIST  
 PyDSTool.fixedpickle.EMPTY\_TUPLE  
 PyDSTool.fixedpickle.EXT1  
 PyDSTool.fixedpickle.EXT2  
 PyDSTool.fixedpickle.EXT4  
 PyDSTool.fixedpickle.FALSE  
 PyDSTool.fixedpickle.FLOAT  
 PyDSTool.fixedpickle.GET  
 PyDSTool.fixedpickle.GLOBAL  
 PyDSTool.fixedpickle.HIGHEST\_PROTOCOL  
 PyDSTool.fixedpickle.INST  
 PyDSTool.fixedpickle.INT  
 PyDSTool.fixedpickle.LIST  
 PyDSTool.fixedpickle.LONG  
 PyDSTool.fixedpickle.LONG1  
 PyDSTool.fixedpickle.LONG4  
 PyDSTool.fixedpickle.LONG\_BINGET  
 PyDSTool.fixedpickle.LONG\_BINPUT  
 PyDSTool.fixedpickle.MARK  
 PyDSTool.fixedpickle.NEWFALSE  
 PyDSTool.fixedpickle.NEWOBJ  
 PyDSTool.fixedpickle.NEWTRUE  
 PyDSTool.fixedpickle.NONE  
 PyDSTool.fixedpickle.OBJ  
 PyDSTool.fixedpickle.PERSID  
 PyDSTool.fixedpickle.POP  
 PyDSTool.fixedpickle.POP\_MARK  
 PyDSTool.fixedpickle.PROTO  
 PyDSTool.fixedpickle.PUT  

PyDSTool.fixedpickle.PyStringMap

PyDSTool.fixedpickle.REDUCE  
 PyDSTool.fixedpickle.SETITEM  
 PyDSTool.fixedpickle.SETITEMS  
 PyDSTool.fixedpickle.SHORT\_BINSTRING  
 PyDSTool.fixedpickle.STOP  
 PyDSTool.fixedpickle.STRING  
 PyDSTool.fixedpickle.TRUE  
 PyDSTool.fixedpickle.TUPLE  
 PyDSTool.fixedpickle.TUPLE1  
 PyDSTool.fixedpickle.TUPLE2  
 PyDSTool.fixedpickle.TUPLE3  
 PyDSTool.fixedpickle.UNICODE  

PyDSTool.fixedpickle.\_tuplesize2code

PyDSTool.fixedpickle.classmap

PyDSTool.fixedpickle.compatible\_formats

PyDSTool.fixedpickle.format\_version

PyDSTool.floor\_divide  
 PyDSTool.fmax  
 PyDSTool.fmin  
 PyDSTool.greater\_equal  
 PyDSTool.index\_exp  
 PyDSTool.inf  
 PyDSTool.infty  
 PyDSTool.integrator'.Inf  

PyDSTool.integrator'.\_all\_int

PyDSTool.integrator'.\_real\_types

PyDSTool.integrator'.isinf  
 PyDSTool.invert  
 PyDSTool.isinf  
 PyDSTool.left\_shift  
 PyDSTool.little\_endian  
 PyDSTool.log1p  
 PyDSTool.logaddexp  
 PyDSTool.logaddexp2  
 PyDSTool.logical\_and  
 PyDSTool.logical\_not  
 PyDSTool.logical\_xor  

PyDSTool.matplotlib\_import.failed

PyDSTool.matplotlib\_import.ver

PyDSTool.maximum  
 PyDSTool.mgrid  
 PyDSTool.minimum  
 PyDSTool.multiply  
 PyDSTool.n  
 PyDSTool.nan  
 PyDSTool.nbytes  
 PyDSTool.negative  
 PyDSTool.newaxis  
 PyDSTool.nextafter  
 PyDSTool.not\_equal  
 PyDSTool.ogrid  
 PyDSTool.ones\_like  

PyDSTool.parseUtils.API

PyDSTool.parseUtils.API\_class  

PyDSTool.parseUtils.Continuous

PyDSTool.parseUtils.DO\_DEC

PyDSTool.parseUtils.DO\_POW

PyDSTool.parseUtils.Discrete

PyDSTool.parseUtils.DomainType  
 PyDSTool.parseUtils.Inf  

PyDSTool.parseUtils.LargestInt32

PyDSTool.parseUtils.Macheps  
 PyDSTool.parseUtils.NAMESEP  
 PyDSTool.parseUtils.NaN  
 PyDSTool.parseUtils.ONES  

PyDSTool.parseUtils.POW\_STR

PyDSTool.parseUtils.TENS

PyDSTool.parseUtils.ZEROS  

PyDSTool.parseUtils.\_all\_complex

PyDSTool.parseUtils.\_all\_float

PyDSTool.parseUtils.\_all\_int

PyDSTool.parseUtils.\_all\_numpy\_complex

PyDSTool.parseUtils.\_all\_numpy\_float

PyDSTool.parseUtils.\_all\_numpy\_int

PyDSTool.parseUtils.\_classes

PyDSTool.parseUtils.\_complex\_types

PyDSTool.parseUtils.\_constants

PyDSTool.parseUtils.\_float\_types

PyDSTool.parseUtils.\_functions

PyDSTool.parseUtils.\_indentstr  

PyDSTool.parseUtils.\_int\_types

PyDSTool.parseUtils.\_num\_equivtype

PyDSTool.parseUtils.\_num\_maxmin

PyDSTool.parseUtils.\_num\_name2equivtypes

PyDSTool.parseUtils.\_num\_name2type

PyDSTool.parseUtils.\_num\_type2name

PyDSTool.parseUtils.\_num\_types

PyDSTool.parseUtils.\_objects

PyDSTool.parseUtils.\_pytypefromtype

PyDSTool.parseUtils.\_real\_types

PyDSTool.parseUtils.\_seq\_types

PyDSTool.parseUtils.\_symbconsts

PyDSTool.parseUtils.\_symbfuncs

PyDSTool.parseUtils.all  

PyDSTool.parseUtils.alphabet\_chars\_RE

PyDSTool.parseUtils.any  
 PyDSTool.parseUtils.argmax  
 PyDSTool.parseUtils.argmin  
 PyDSTool.parseUtils.array  
 PyDSTool.parseUtils.asarray  
 PyDSTool.parseUtils.atleast\_1d  
 PyDSTool.parseUtils.builtinFnSigInfo  
 PyDSTool.parseUtils.builtin\_auxnames  
 PyDSTool.parseUtils.clip  
 PyDSTool.parseUtils.complex  
 PyDSTool.parseUtils.complex128  
 PyDSTool.parseUtils.complex192  
 PyDSTool.parseUtils.complex64  
 PyDSTool.parseUtils.complex\_  
 PyDSTool.parseUtils.complexfloating  

PyDSTool.parseUtils.convert\_power\_reserved\_keywords

PyDSTool.parseUtils.double  
 PyDSTool.parseUtils.exp  
 PyDSTool.parseUtils.finfo  
 PyDSTool.parseUtils.fit\_function  
 PyDSTool.parseUtils.float  
 PyDSTool.parseUtils.float32  
 PyDSTool.parseUtils.float64  
 PyDSTool.parseUtils.float96  
 PyDSTool.parseUtils.float\_  
 PyDSTool.parseUtils.floating  
 PyDSTool.parseUtils.greater  
 PyDSTool.parseUtils.int  
 PyDSTool.parseUtils.int0  
 PyDSTool.parseUtils.int16  
 PyDSTool.parseUtils.int32  
 PyDSTool.parseUtils.int64  
 PyDSTool.parseUtils.int8  
 PyDSTool.parseUtils.int\_  
 PyDSTool.parseUtils.integer  
 PyDSTool.parseUtils.intersect  
 PyDSTool.parseUtils.isfinite  
 PyDSTool.parseUtils.less  
 PyDSTool.parseUtils.less\_equal  
 PyDSTool.parseUtils.log  
 PyDSTool.parseUtils.logical\_or  
 PyDSTool.parseUtils.mat  
 PyDSTool.parseUtils.minpack  
 PyDSTool.parseUtils.name\_chars\_RE  
 PyDSTool.parseUtils.ndarray  
 PyDSTool.parseUtils.norm  
 PyDSTool.parseUtils.npy  

PyDSTool.parseUtils.null\_predicate

PyDSTool.parseUtils.null\_predicate\_class  
 PyDSTool.parseUtils.num\_chars  
 PyDSTool.parseUtils.ones  
 PyDSTool.parseUtils.os  
 PyDSTool.parseUtils.predicate\_op  
 PyDSTool.parseUtils.protected\_allnames  
 PyDSTool.parseUtils.protected\_auxnamesDB  
 PyDSTool.parseUtils.protected\_macronames  
 PyDSTool.parseUtils.protected\_mathnames  
 PyDSTool.parseUtils.protected\_randomnames  
 PyDSTool.parseUtils.protected\_scipynames  
 PyDSTool.parseUtils.protected\_specialfns  
 PyDSTool.parseUtils.putmask  
 PyDSTool.parseUtils.remain  

PyDSTool.parseUtils.reserved\_keywords

PyDSTool.parseUtils.s

PyDSTool.parseUtils.searchsorted  
 PyDSTool.parseUtils.shape  
 PyDSTool.parseUtils.sign  

PyDSTool.parseUtils.specialfns

PyDSTool.parseUtils.spy  
 PyDSTool.parseUtils.sqrt  
 PyDSTool.parseUtils.swapaxes  
 PyDSTool.parseUtils.syms  
 PyDSTool.parseUtils.sys  
 PyDSTool.parseUtils.take  

PyDSTool.parseUtils.targetLangs

PyDSTool.parseUtils.time  
 PyDSTool.parseUtils.types  
 PyDSTool.parseUtils.zeros  
 PyDSTool.r\_  
 PyDSTool.rad2deg  
 PyDSTool.reciprocal  
 PyDSTool.remainder  
 PyDSTool.right\_shift  
 PyDSTool.rint  
 PyDSTool.s\_  
 PyDSTool.sctypeDict  
 PyDSTool.sctypeNA  
 PyDSTool.sctypes  
 PyDSTool.signbit  
 PyDSTool.spacing  
 PyDSTool.square  
 PyDSTool.subtract  
 PyDSTool.t  
 PyDSTool.true\_divide  
 PyDSTool.trunc  
 PyDSTool.typeDict  
 PyDSTool.typeNA  
 PyDSTool.typecodes  

PyDSTool.utils.API

PyDSTool.utils.API\_class  

PyDSTool.utils.Continuous

PyDSTool.utils.Discrete

PyDSTool.utils.DomainType  

PyDSTool.utils.Inf

PyDSTool.utils.LargestInt32

PyDSTool.utils.Macheps  

PyDSTool.utils.NaN

PyDSTool.utils.\_1DimplicitSolveMethods  

PyDSTool.utils.\_all\_complex

PyDSTool.utils.\_all\_float

PyDSTool.utils.\_all\_int

PyDSTool.utils.\_all\_numpy\_complex

PyDSTool.utils.\_all\_numpy\_float

PyDSTool.utils.\_all\_numpy\_int

PyDSTool.utils.\_classes

PyDSTool.utils.\_complex\_types

PyDSTool.utils.\_float\_types

PyDSTool.utils.\_functions

PyDSTool.utils.\_implicitSolveMethods  

PyDSTool.utils.\_int\_types

PyDSTool.utils.\_mappings

PyDSTool.utils.\_num\_equivtype

PyDSTool.utils.\_num\_maxmin

PyDSTool.utils.\_num\_name2equivtypes

PyDSTool.utils.\_num\_name2type

PyDSTool.utils.\_num\_type2name

PyDSTool.utils.\_num\_types

PyDSTool.utils.\_pytypefromtype

PyDSTool.utils.\_real\_types

PyDSTool.utils.\_seq\_types

PyDSTool.utils.all  
 PyDSTool.utils.any  
 PyDSTool.utils.argmax  
 PyDSTool.utils.atleast\_1d  
 PyDSTool.utils.clip  
 PyDSTool.utils.complex  
 PyDSTool.utils.complex128  
 PyDSTool.utils.complex192  
 PyDSTool.utils.complex64  
 PyDSTool.utils.complex\_  
 PyDSTool.utils.complexfloating  
 PyDSTool.utils.deepcopy  
 PyDSTool.utils.double  
 PyDSTool.utils.exp  
 PyDSTool.utils.finfo  
 PyDSTool.utils.fit\_function  
 PyDSTool.utils.float  
 PyDSTool.utils.float32  
 PyDSTool.utils.float96  
 PyDSTool.utils.float\_  
 PyDSTool.utils.floating  

PyDSTool.utils.greater

PyDSTool.utils.int  
 PyDSTool.utils.int0  
 PyDSTool.utils.int16  
 PyDSTool.utils.int64  
 PyDSTool.utils.int8  
 PyDSTool.utils.int\_  
 PyDSTool.utils.integer  

PyDSTool.utils.isfinite

PyDSTool.utils.less

PyDSTool.utils.less\_equal  
 PyDSTool.utils.log  
 PyDSTool.utils.logical\_or  
 PyDSTool.utils.mat  
 PyDSTool.utils.npy  

PyDSTool.utils.null\_predicate

PyDSTool.utils.null\_predicate\_class  
 PyDSTool.utils.ones  
 PyDSTool.utils.predicate\_op  
 PyDSTool.utils.putmask  

PyDSTool.utils.rerr

PyDSTool.utils.rout

PyDSTool.utils.shape  
 PyDSTool.utils.sign  
 PyDSTool.utils.spy  
 PyDSTool.utils.sqrt  

PyDSTool.utils.targetLangs

PyDSTool.utils.types  
 PyDSTool.verstr\_parts  
 cPickle.HIGHEST\_PROTOCOL  
 cPickle.compatible\_formats  
 cPickle.format\_version  
 matplotlib.pylab.ALLOW\_THREADS  
 matplotlib.pylab.BUFSIZE  
 matplotlib.pylab.CLIP  
 matplotlib.pylab.DAILY  
 matplotlib.pylab.ERR\_CALL  
 matplotlib.pylab.ERR\_DEFAULT  
 matplotlib.pylab.ERR\_DEFAULT2  
 matplotlib.pylab.ERR\_IGNORE  
 matplotlib.pylab.ERR\_LOG  
 matplotlib.pylab.ERR\_PRINT  
 matplotlib.pylab.ERR\_RAISE  
 matplotlib.pylab.ERR\_WARN  
 matplotlib.pylab.FLOATING\_POINT\_SUPPORT  
 matplotlib.pylab.FPE\_DIVIDEBYZERO  
 matplotlib.pylab.FPE\_INVALID  
 matplotlib.pylab.FPE\_OVERFLOW  
 matplotlib.pylab.FPE\_UNDERFLOW  
 matplotlib.pylab.FR  
 matplotlib.pylab.False\_  
 matplotlib.pylab.HOURLY  
 matplotlib.pylab.Inf  
 matplotlib.pylab.Infinity  
 matplotlib.pylab.MAXDIMS  
 matplotlib.pylab.MINUTELY  
 matplotlib.pylab.MO  
 matplotlib.pylab.MONTHLY  
 matplotlib.pylab.NAN  
 matplotlib.pylab.NINF  
 matplotlib.pylab.NZERO  
 matplotlib.pylab.NaN  
 matplotlib.pylab.PINF  
 matplotlib.pylab.PZERO  
 matplotlib.pylab.RAISE  
 matplotlib.pylab.SA  
 matplotlib.pylab.SECONDLY  
 matplotlib.pylab.SHIFT\_DIVIDEBYZERO  
 matplotlib.pylab.SHIFT\_INVALID  
 matplotlib.pylab.SHIFT\_OVERFLOW  
 matplotlib.pylab.SHIFT\_UNDERFLOW  
 matplotlib.pylab.SU  
 matplotlib.pylab.ScalarType  
 matplotlib.pylab.TH  
 matplotlib.pylab.TU  
 matplotlib.pylab.True\_  
 matplotlib.pylab.UFUNC\_BUFSIZE\_DEFAULT  
 matplotlib.pylab.UFUNC\_PYVALS\_NAME  
 matplotlib.pylab.WE  
 matplotlib.pylab.WEEKLY  
 matplotlib.pylab.WRAP  
 matplotlib.pylab.YEARLY  
 matplotlib.pylab.absolute  
 matplotlib.pylab.add  
 matplotlib.pylab.arccos  
 matplotlib.pylab.arccosh  
 matplotlib.pylab.arcsin  
 matplotlib.pylab.arcsinh  
 matplotlib.pylab.arctan  
 matplotlib.pylab.arctan2  
 matplotlib.pylab.arctanh  
 matplotlib.pylab.bitwise\_and  
 matplotlib.pylab.bitwise\_not  
 matplotlib.pylab.bitwise\_or  
 matplotlib.pylab.bitwise\_xor  
 matplotlib.pylab.c\_  
 matplotlib.pylab.cast  
 matplotlib.pylab.ceil  
 matplotlib.pylab.colorbar\_doc  
 matplotlib.pylab.conj  
 matplotlib.pylab.conjugate  
 matplotlib.pylab.copysign  
 matplotlib.pylab.cos  
 matplotlib.pylab.cosh  
 matplotlib.pylab.deg2rad  
 matplotlib.pylab.degrees  
 matplotlib.pylab.divide  
 matplotlib.pylab.e  
 matplotlib.pylab.equal  
 matplotlib.pylab.exp  
 matplotlib.pylab.exp2  
 matplotlib.pylab.expm1  
 matplotlib.pylab.fabs  
 matplotlib.pylab.floor  
 matplotlib.pylab.floor\_divide  
 matplotlib.pylab.fmax  
 matplotlib.pylab.fmin  
 matplotlib.pylab.fmod  
 matplotlib.pylab.frexp  
 matplotlib.pylab.greater  
 matplotlib.pylab.greater\_equal  
 matplotlib.pylab.hypot  
 matplotlib.pylab.index\_exp  
 matplotlib.pylab.inf  
 matplotlib.pylab.infty  
 matplotlib.pylab.invert  
 matplotlib.pylab.isfinite  
 matplotlib.pylab.isinf  
 matplotlib.pylab.isnan  
 matplotlib.pylab.ldexp  
 matplotlib.pylab.left\_shift  
 matplotlib.pylab.less  
 matplotlib.pylab.less\_equal  
 matplotlib.pylab.little\_endian  
 matplotlib.pylab.log  
 matplotlib.pylab.log10  
 matplotlib.pylab.log1p  
 matplotlib.pylab.logaddexp  
 matplotlib.pylab.logaddexp2  
 matplotlib.pylab.logical\_and  
 matplotlib.pylab.logical\_not  
 matplotlib.pylab.logical\_or  
 matplotlib.pylab.logical\_xor  
 matplotlib.pylab.maximum  
 matplotlib.pylab.mgrid  
 matplotlib.pylab.minimum  
 matplotlib.pylab.mod  
 matplotlib.pylab.modf  
 matplotlib.pylab.multiply  
 matplotlib.pylab.nan  
 matplotlib.pylab.nbytes  
 matplotlib.pylab.negative  
 matplotlib.pylab.newaxis  
 matplotlib.pylab.nextafter  
 matplotlib.pylab.not\_equal  
 matplotlib.pylab.ogrid  
 matplotlib.pylab.ones\_like  
 matplotlib.pylab.pi  
 matplotlib.pylab.r\_  
 matplotlib.pylab.rad2deg  
 matplotlib.pylab.radians  
 matplotlib.pylab.rcParams  
 matplotlib.pylab.rcParamsDefault  
 matplotlib.pylab.reciprocal  
 matplotlib.pylab.remainder  
 matplotlib.pylab.right\_shift  
 matplotlib.pylab.rint  
 matplotlib.pylab.s\_  
 matplotlib.pylab.sctypeDict  
 matplotlib.pylab.sctypeNA  
 matplotlib.pylab.sctypes  
 matplotlib.pylab.sign  
 matplotlib.pylab.signbit  
 matplotlib.pylab.sin  
 matplotlib.pylab.sinh  
 matplotlib.pylab.spacing  
 matplotlib.pylab.sqrt  
 matplotlib.pylab.square  
 matplotlib.pylab.subtract  
 matplotlib.pylab.tan  
 matplotlib.pylab.tanh  
 matplotlib.pylab.true\_divide  
 matplotlib.pylab.trunc  
 matplotlib.pylab.typeDict  
 matplotlib.pylab.typeNA  
 matplotlib.pylab.typecodes  
 scipy.integrate.vode.dvode  
 scipy.integrate.vode.zvode  

---

[hide private]
